# The Evolutionary Dynamics of Influenza A Viruses Circulating in Mallards in Duck Hunting Preserves in Maryland, USA

**DOI:** 10.3390/microorganisms9010040

**Published:** 2020-12-25

**Authors:** Nídia S. Trovão, Jacqueline M. Nolting, Richard D. Slemons, Martha I. Nelson, Andrew S. Bowman

**Affiliations:** 1 Division of International Epidemiology and Population Studies, Fogarty International Center, National Institutes of Health, Bethesda, MD 20814, USA; nidia.trovao@nih.gov (N.S.T.); nelsonma@mail.nih.gov (M.I.N.); 2 Department of Microbiology, Icahn School of Medicine at Mount Sinai, New York, NY 10029, USA; 3 Global Health and Emerging Pathogens Institute, Icahn School of Medicine at Mount Sinai, New York, NY 10029, USA; 4 Department of Veterinary Preventive Medicine, The Ohio State University, Columbus, OH 43210, USA; nolting.4@osu.edu (J.M.N.); slemons.1@osu.edu (R.D.S.)

**Keywords:** influenza A virus, avian, evolution, phylogenetic analysis, Bayesian analysis

## Abstract

Duck hunting preserves (DHP) have resident populations of farm-raised mallard ducks, which create potential foci for the evolution of novel influenza A viruses (IAVs). Through an eleven-year (2003–2013) IAV surveillance project in seven DHPs in Maryland, USA, we frequently identified IAVs in the resident, free-flying mallard ducks (5.8% of cloacal samples were IAV-positive). The IAV population had high genetic diversity, including 12 HA subtypes and 9 NA subtypes. By sequencing the complete genomes of 290 viruses, we determined that genetically diverse IAVs were introduced annually into DHP ducks, predominantly from wild birds in the *Anatidae* family that inhabit the Atlantic and Mississippi flyways. The relatively low viral gene flow observed out of DHPs suggests that raised mallards do not sustain long-term viral persistence nor do they serve as important sources of new viruses in wild birds. Overall, our findings indicate that DHPs offer reliable samples of the diversity of IAV subtypes, and could serve as regional sentinel sites that mimic the viral diversity found in local wild duck populations, which would provide a cost-efficient strategy for long-term IAV monitoring. Such monitoring could allow for early identification and characterization of viruses that threaten bird species of high economic and environmental interest.

## 1. Introduction

The high genetic diversity of avian influenza A viruses (IAVs) circulating in waterfowl (*Anatidae*) and shorebirds (*Neoaves*) [1,2] presents an ongoing threat of spillover into poultry, and potential economic losses. IAVs that circulate in poultry can also infect humans, causing severe disease outbreaks locally, as well as potentially igniting a new global pandemic [3]. Consequently, there is great interest in understanding how infected wild birds disseminate IAVs spatially along migratory flyways, which facilitates the long-distance spread of IAVs between locations [4,5]. Long distance spread creates a troublesome scenario in which IAVs that evolve in one region can quickly spread to other locations through wild bird movements and mix with local viruses to create novel reassortants [6]. The genetic diversity and evolution of IAVs in North American wild birds have been tracked for several decades, particularly in locations where wild birds are known to congregate during migration [7]. Migratory flyways have been shown to shape the population structure of IAV in wild birds in North America [8]. In 2014, the introduction of highly pathogenic H5 viruses from Asia into North American wild birds and poultry led to severe economic losses, further intensifying interest in the role of wild bird populations in maintaining and disseminating IAVs that present a threat to poultry and humans [9].

Duck hunters actively contribute to IAV research by providing academic and government researchers with access to harvested ducks for IAV testing and surveillance. Duck hunting preserves (DHPs) have been operating on the eastern shores of Maryland for decades. By feeding high densities of farm-raised mallards outdoors, DHPs potentially create conditions that are conducive to long-term transmission of IAVs. DHP birds share habitat and freely intermingle with wild birds, and potentially play a role in local IAV ecology and evolution. It is worth noting that even wild mallards have comparatively high rates of IAV infection.

We sought to understand the role of DHPs in the epidemiology and ecology of IAVs, and we hypothesized that DHPs could serve as regional sentinel sites that mimic the viral diversity found in local wild duck populations. By applying phylodynamic methods based on a Bayesian framework, we determined that the IAV populations identified in the DHPs are shaped primarily by asymmetrical inbound viral gene flow from wild birds, with little evidence that the resident mallards sustained viral transmission beyond a single season.

## 2. Materials and Methods

### 2.1. Sampling and Laboratory Diagnosis

Cloacal and/or fecal swabs (*n* = 12,714) were collected 2003–2013 from resident, free-flying mallards at seven Maryland DHPs under the animal use protocol 2007A0148 approved by the The Ohio State University Institutional Animal Care and Use Committee. DHPs included in this study either raise their mallards locally or acquire them as hatchlings or fledglings from other states located in the Atlantic or Mississippi flyway [10]. The DHPs were located within an approximate 40-mile radius of each other on the Delmarva peninsula, situated between Delaware Bay and the Chesapeake Bay (Supplementary Figure S1). All seven DHPs are located within 100 miles of Delaware Bay, an established hotspot for avian IAVs when high densities of shorebirds congregate during migration [11] (Supplementary Figure S1). Maryland’s eastern shore also hosts a high density of chicken farms. Cloacal swabs were collected from trapped mallards during summer months and from hunter-harvested ducks during autumn and winter months. The number of samples varied by year and location, but all seven of the DHPs provided samples for the majority of years (Table 1). When trapping was not feasible, swabs of fresh fecal material were used in lieu cloacal swabs. Swabs were placed in vials containing viral transport media (brain–heart infusion broth with penicillin and streptomycin) and stored at −80 °C until testing could be initiated. IAVs were isolated using 10-day-old embryonating chicken eggs, as previously described [10,12,13].

### 2.2. Viral RNA Extraction, Whole-Genome Sequencing, Assembly and Annotation

Viral RNA was extracted using Trizol Reagent (Invitrogen Corp, Carlsbad, CA, USA) or using the ZR 96 Viral RNA kit (Zymo Research Corporation, Irvine, CA, USA), as previously described [10,13]. Full-length genomic sequencing was completed as previously described using targeted multisegment reverse transcription-PCR followed by sequencing with 454 GS FLX+ and Illumina HiSeq sequencing technologies [10].

### 2.3. Phylogenetic Analysis

After removing duplicate sequences based on genetic composition, location and collection date in the PB2 dataset, we enriched the datasets of 146 IAV full-genomes sequences. This was accomplished by retrieving all IAV whole-genome sequences collected from 2000 to 2013 from GenBank on 7 March 2017 (Supplementary Table S1). We annotated all sequences with their information regarding superorder, habitat, flyway and region in the USA. The data were aligned using MAFFT v7.310 [14] and subsequently manually edited. Maximum likelihood (ML) trees were inferred using RAxML v7.2.6 [15] incorporating a general time-reversible (GTR) model of nucleotide substitution with a gamma-distributed (Γ) rate variation among sites.

We also investigated the temporal signal of the datasets using TempEst v1.5.1 [16]. Phylogenetic relationships were inferred for each of the segments’ datasets separately (711 IAV sequences for each segment) with a Bayesian phylogenetic approach using Markov chain Monte Carlo (MCMC) available via the BEAST v1.10.4 package [17] and the high-performance computational capabilities of the Biowulf Linux cluster at the National Institutes of Health, Bethesda, MD, USA (http://biowulf.nih.gov). We used an uncorrelated relaxed molecular clock with branch rates drawn form a lognormal distribution to account for evolutionary rate variation among lineages, with a Skygrid demographic prior [18], and a general-time reversible (GTR) model of nucleotide substitution with gamma-distributed rate variation among sites. For sequences with only the year of viral collection available, the lack of tip date precision was accommodated by sampling uniformly across a one-year window from 1 January to 30 December. The MCMC chain was run separately at least three times for each of the data sets and for at least 200 million iterations with sub-sampling every 20,000 iterations, using the BEAGLE [19] library to improve computational performance. All parameters reached convergence, as assessed visually using Tracer v.1.7.1, with statistical uncertainty reflected in values of the 95% highest posterior density (HPD). At least 10% of the chain was removed as burn-in. A subset of 500 trees was selected from the combined posterior distribution and used as an empirical distribution in the subsequent discrete diffusion inference. Following Pagel et al. (2004) [20], we achieved this by incorporating a proposal mechanism that randomly draws a new tree from the empirical distribution [21].

We estimated spatial diffusion dynamics among a set of flyways, habitats, avian taxonomic groups and regions using a Bayesian discrete phylogeographic approach [22]. This approach conditions on the trait information recorded at the tips of the empirical segment phylogenies and models the transition history among those states as a continuous time Markov chain (CTMC) process, allowing the inference of unobserved states at the ancestral nodes in each tree of the posterior distribution. We used a nonreversible CTMC model [23] and incorporated a Bayesian stochastic search variable selection to identify a sparse set of transition rates that adequately summarized the epidemiological connectivity [22]. As part of these analyses, we also incorporated posterior inference of the complete Markov jump history through time [24], allowing us to quantify state transitions and the time spent in a particular trait state along each phylogenetic branch. Maximum clade credibility (MCC) trees were summarized using TreeAnnotator v1.10.4 and visualized in FigTree v1.4.4. The evolutionary rates across segments (ranging from 1.84 × 10^−3^ to 3.47 × 10^−3^) are consistent with previously characterized measures for influenza A virus [25].

We evaluated the genetic diversity between viruses collected in the DHPs and those circulating in the Atlantic flyway or the USA (including the Atlantic flyway), by estimating the percentage of HA-NA subtype combinations identified per year in each of the above-mentioned populations. These were illustrated using heatmap functions available in the R statistical software [26].

## 3. Results

### 3.1. IAVs Detected in DHPs

**Figure 1:**
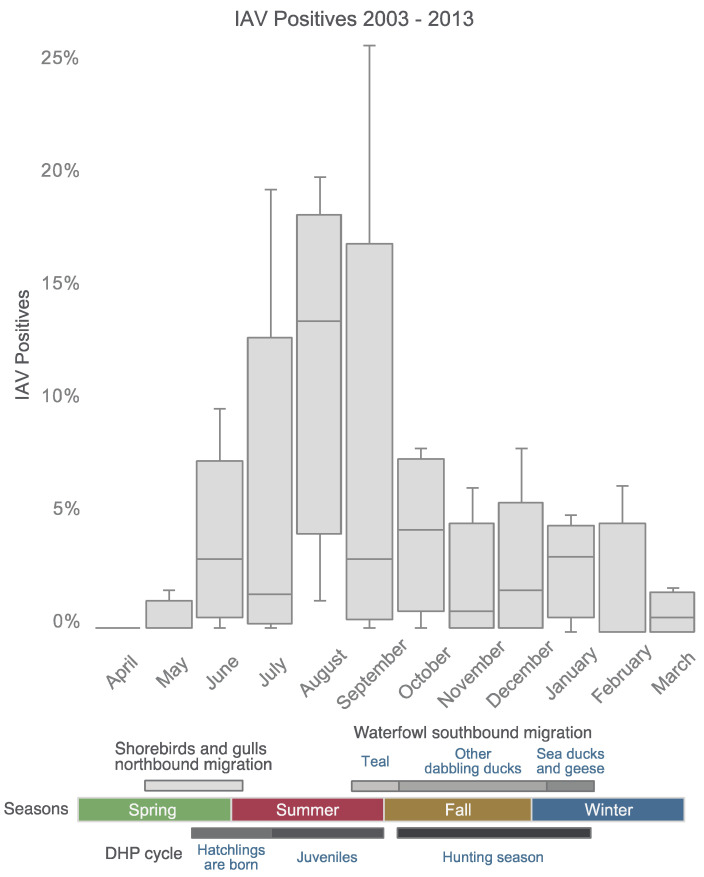
Seasonal timing of influenza A viruses (IAVs) in Maryland duck hunting preserves. The proportion of samples collected from mallards in the DHPs during each month that tested positive for IAV is indicated by the height of the column. The upper and lower whiskers represent the maximum and minimum, respectively. From top to bottom, the first, second and third lines of the rectangles represent the first, second (median) and third quartiles. Data are summarized over eleven years from 2003–2013. Below, the seasonal timing of when DHP mallards hatch and become susceptible juveniles is described, as well as the arrival to the Delmarva Peninsula of shorebirds and gulls during their northbound migration, and migratory waterfowl during their southbound migration.

In 2003–2013, 12,714 cloacal samples were collected from mallards at seven DHPs in Maryland, and 740 tested positive (~5.8%, Table 1) for IAV. Complete IAV genomes were successfully sequenced from 290 samples, which represent all 11 years of the study (Table 2). IAVs were isolated every year from DHP_1_, the largest of the DHPs in terms of acreage. As expected, the highest proportion of IAV-positive samples occurred in August and September, immediately following the emergence of large numbers of immunologically naïve hatch-year resident, free-flying mallards (Figure 1) in which the virus can rapidly spread.

### 3.2. Genetic Diversity of IAV Circulating in the DHPs

**Figure 2:**
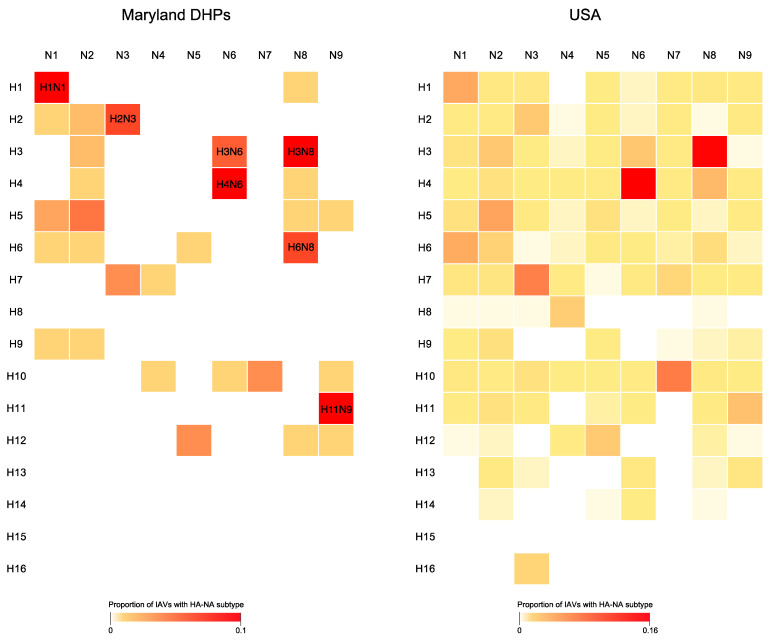
IAV subtypes in Maryland duck hunting preserves. The proportion of IAVs with different subtype combinations of the surface antigens HA and NA is depicted in heatmaps constructed for the DHPs (white = 0; red = 10%) and entire USA, excluding viruses isolated in poultry (white = 0; red = 16%), summarized over the 11 years of the study.

We identified 32 HA-NA subtype combinations of IAV in the DHPs. The most frequently observed subtypes were H1N1, H2N3, H3N8, H4N6 and H11N9 (Figure 2) [27]. The dominant HA subtype was different every year, with evidence of cycling (Supplementary Figure S2). For example, H3 was the dominant HA subtype in 2005, 2010, and 2012. H4 was never the predominant HA subtype but was identified at high frequencies during 8 of 11 years. The H5 (HA) subtype was predominant in 2007 and 2011 (22% and 36%, respectively) (Supplementary Figure S2). Note that these were low-pathogenicity IAVs and no highly pathogenic IAVs were detected at any time in the DHP mallards. In general, we observed high congruity between the frequency of IAV subtypes in the Maryland DHPs and in wild birds in the Atlantic flyway and in wild birds in the United States as a whole (Figure 2 and Supplementary Figure S2). The dominant HA subtype circulating each year in the DHPs matched the dominant (H3) subtype circulating in the US in almost a third (27%) of the years during the 11-year period (Figure 2). Of note, all HA-NA subtype combinations detected in DHPs were also detected in the Atlantic flyway or USA-wide (Supplementary Figure S3). We also compared the IAV HA subtype diversity in USA *Anatidae*, USA *Neoaves* and DHP mallards, and observed that the HA-NA subtypes recovered from mallards in the DHPs were more similar to those circulating in *Anatidae* than *Neoaves* (Supplementary Figure S4).

**Figure 3:**
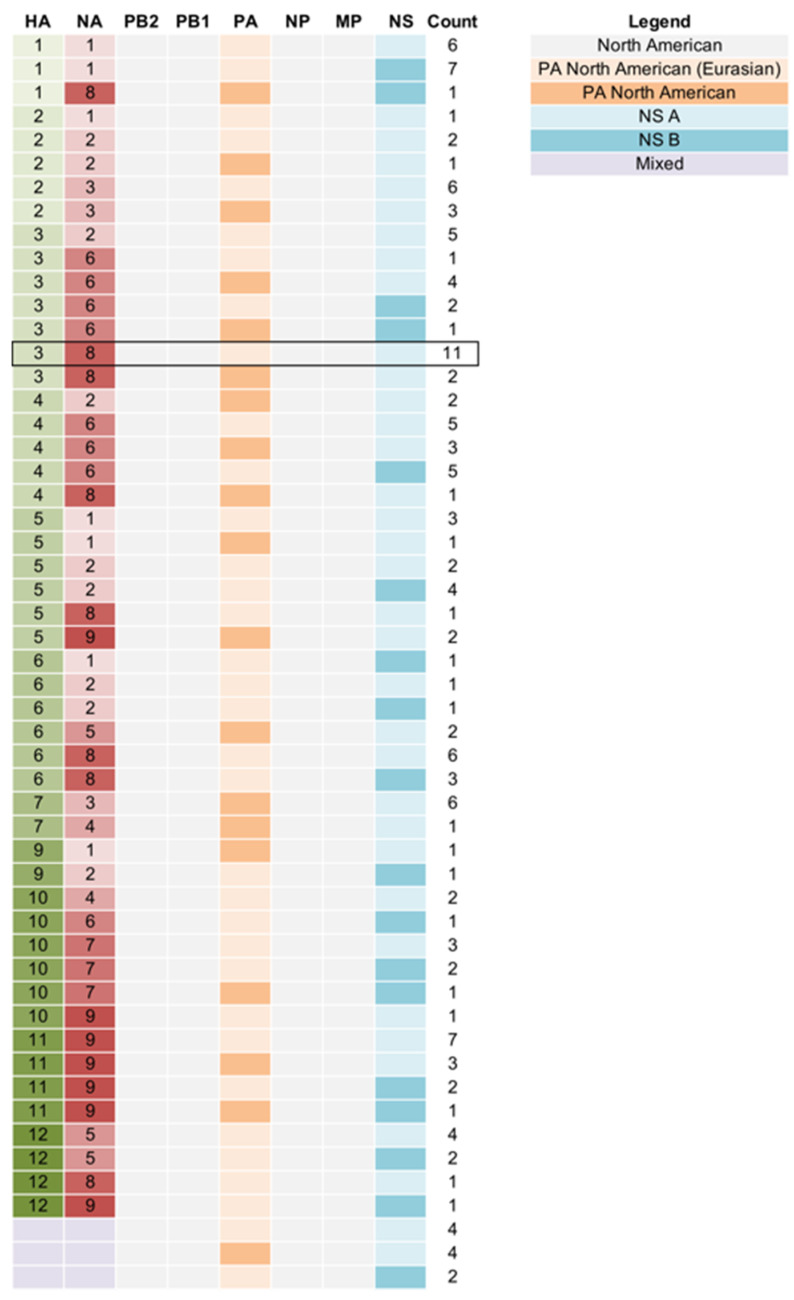
Genetic diversity of IAVs circulating in birds in Maryland. We identified 53 genotypes by surveillance of mallards in Maryland DHPs 2003–2013. Each rectangle represents 1 of the 8 segments of the virus genome. The two key surface antigens HA and NA are listed first, followed by the 6 remaining gene segments. Shading of each rectangle corresponds to major genetic lineages of (i) the HA and NA, which are sorted by subtype; (ii) the PA segment, which was classified into two North American lineages (North American and North American (Eurasian)); and (iii) the NS segment was sorted according to A and B alleles. All remaining internal segments were classified as belonging to a single North American lineage. Rectangles shaded purple represent mixed HA-NA infections. The black box indicates the most frequently observed genome constellation. The rightmost column indicates the number of viruses from each genotype identified in the DHPs.

Additional genetic diversity was evident when all eight segments of the viral genome were considered, resulting in a total of 53 individual genotype constellations [28,29] (Figure 3). All viruses had genes belonging to North American IAV lineages, with no evidence of recent incursions of viruses of Eurasian origin that have been previously described [30,31]. However, the majority (73%) of viruses characterized in this study had a polymerase (PA) segment that had been introduced from Eurasia into North America over two decades ago [32,33]. As expected, the majority (74%) of viruses had the NS A allele, compared to 26% with the NS B allele.

### 3.3. Multiple Introductions of IAV into the DHPs during the Study Period

**Figure 4:**
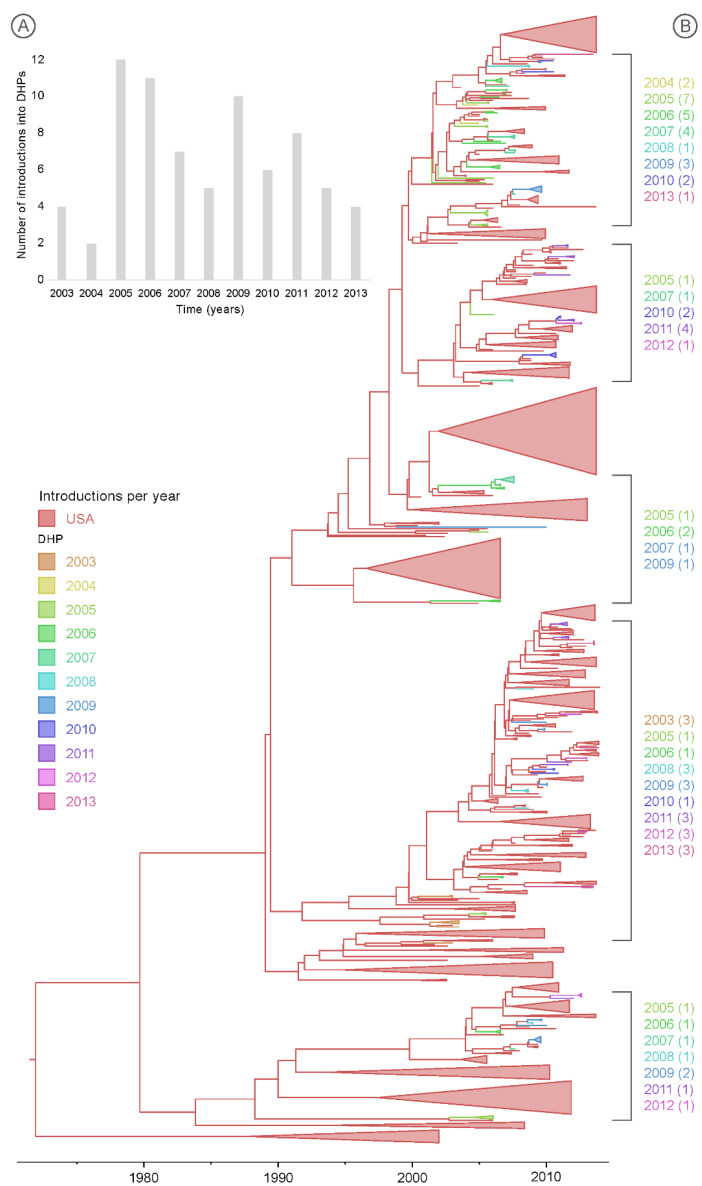
Viral introductions into Maryland duck hunting preserves. (**A**) The estimated number of discrete introductions of IAVs per year into the DHPs, inferred by “Markov jumps” observed on the PB2 phylogeny. (**B**) Maximum clade credibility (MCC) tree inferred for the PB2 segment. The shade of the branch indicates the inferred location state: red branches indicate USA birds from all years, while other colors indicate Maryland DHPs, shaded by year. IAV introductions into Maryland DHPs from 2003–2013 are labeled. Phylogenies for remaining internal gene segments are provided in Supplementary Figure S5.

The similarities in the IAV populations observed in the Maryland DHPs and wild birds across the United States appear to result from high levels of viral gene flow into the DHPs from both the Atlantic and Mississippi flyways. Each year, multiple new viruses were introduced independently into the seven DHPs, even in years with less sampling (Table 1, Figure 4B, phylogenies for other segments provided in Supplementary Figure S5). The number of discrete viral introductions per year ranged from 2 to 12 (average ≈ 7 introductions per year). The highest number of IAV introductions (>10) into the Maryland DHPs occurred in 2005, 2006 and 2009 (Figure 4A). No strong evidence of IAV persistence across multiple years was observed in any single DHP [34]. The possibility of viral persistence for about one year across two DHPs cannot be excluded in the case of four viral sequences (H2N2 and H2N1) that consistently clustered together on multiple phylogenetic trees inferred for different genome segments (Figure 4A and Supplementary Figure S5).

### 3.4. Spatial Dynamics of IAV in DHPs

**Figure 5:**
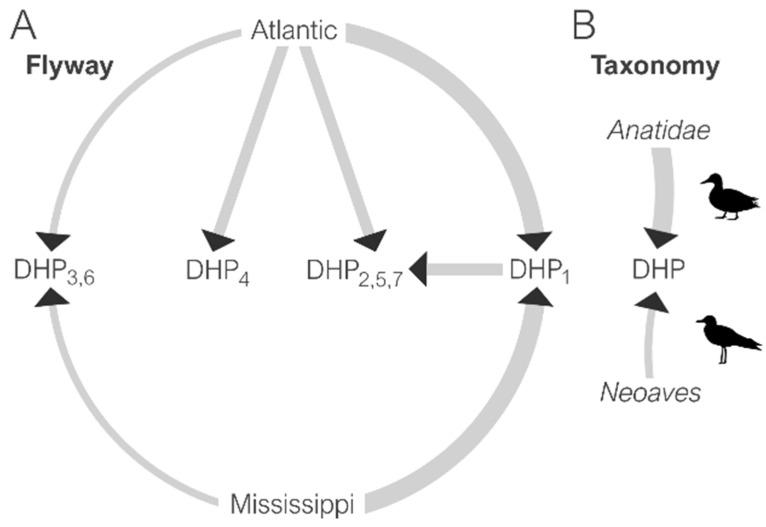
Dissemination of IAVs among flyways, taxonomic groups and duck hunting preserves. The magnitude of viral gene flow, quantified by Markov jumps (available in Supplementary Tables S2–S6), is proportional to the width of the arrow between USA flyways (**A**) and avian taxonomic group (**B**). Specifically, the arrow width represents the relative proportion of viruses seeded into a given DHP from various sources, inferred across phylogenies for all segments (thin arrow—low proportion of viral introductions attributed to this source; thick arrow—high proportion of viral introductions attributed to this source).

To determine the ecological sources of the IAVs imported into the DHPs each year, ancestral reconstructions of traits were inferred along the branches of the phylogenetic trees for each of the eight segments of the viral genome. As expected, dabbling ducks from the *Anatidae* order were identified as the most likely source of the viruses imported into the DHPs (Figure 5B, Supplementary Figure S6 and Supplementary Tables S2–S6). Geographically, both the Atlantic and Mississippi flyways were likely sources of IAVs in the DHPs (Figure 5A and Supplementary Figure S6 and Supplementary Tables S2–S6). A higher contribution of viruses from the Mississippi flyway into a particular DHP was consistent with its anecdotal practice of acquiring juveniles from the Mississippi flyway, and purchased birds could be another mechanism by which viruses are introduced into a DHP.

**Figure 6:**
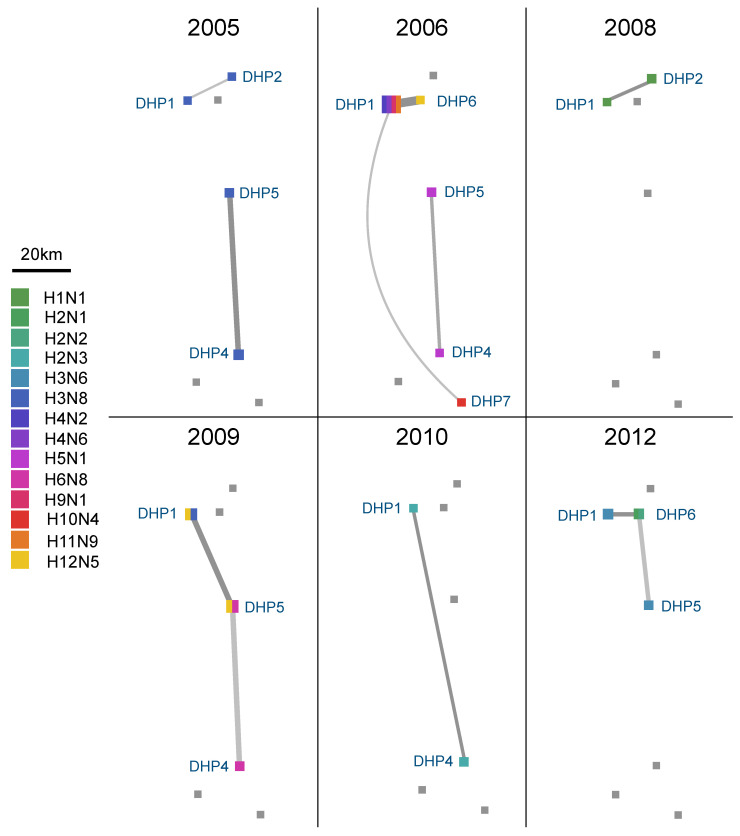
Viral connectivity map between DHPs. Lines represent inferred viral movements between genetically similar IAVs collected from DHPs that clustered together in the same monophyletic clade in the PB2 MCC tree. Line thickness is proportional to the number of viruses in the monophyletic clade. Squares represent DHPs, where size is proportional to the number of viruses for each DHP, and color represents the subtypes circulating in the DHP. Distances between DHPs on the map are proportional to real-world distances.

A limited volume of viral gene flow was observed between DHPs during six of the eleven years of the study (Figure 6). No consistent patterns of connection between any pairs of DHPs were observed across years. However, DHP_1_ tended to be a source of IAVs for other smaller DHPs (Figure 5A and Supplementary Figure S6 and Supplementary Tables S2–S6). This pattern could relate to particular ecological characteristics of the site, including its larger size, which potentially provides greater opportunities for intermingling with wild birds [35] of the same or different taxonomic group [36]. Overall, however, DHPs acquired far more IAVs from wild bird populations than from neighboring DHPs.

## 4. Discussion

The genetic diversity of IAVs maintained in wild bird populations occasionally spills over into poultry farms, domestic mammals, and humans [9], presenting an ongoing global threat to agriculture and public health. Despite decades of intensive surveillance of IAV dynamics in wild birds, there are still important outstanding questions about how viruses disseminate spatially through migrating wild bird populations, whether certain locations where high densities of birds congregate serve as key drivers of viral evolution and genomic reassortment, and how avian immune responses to different antigens might drive patterns of viral diversity such as subtype cycling. Although waterfowl hunters have been helpful partners in the study of IAV dynamics in wild aquatic birds for many decades, there has been little direct surveillance of DHPs, which have proliferated as wetland habitats have diminished in the United States. Through longitudinal study of intensively sampled duck populations in seven discrete but neighboring locations, our study characterizes the IAVs identified in DHPs and determines their contribution to the evolution of IAV in the eastern United States. Notably, high genetic diversity of IAVs was observed in the DHPs each year, resulting primarily from multiple introductions of IAVs from wild waterfowl located in the Atlantic and Mississippi flyways and reflecting high rates of intermingling between DHP-raised and wild birds [35]. The viral diversity circulating in the DHPs each year largely reflects what was circulating regionally in wild birds [27,37]. The presence of viruses related to those circulating in the Mississippi flyway could relate to a proportion of resident, free-flying mallards on the Maryland DHPs originating from locations situated in this flyway, or from the widespread movement of birds, which do not necessarily adhere to the four north-south flyways loosely defined across the United States [38] by researchers and bird enthusiasts. The IAV subtypes observed in the DHPs also mirrored those observed in wild birds regionally, including the most frequently detected H3N8 combination.

Overall, our study indicates that DHPs harbor genetically diverse IAV populations, but do not necessarily act as locations where new viral diversity is generated or maintained long term, and are not likely to be an important source of IAVs for the Maryland poultry industry (scarcity of IAV genetic sequences collected from chickens in Maryland in GenBank; Supplementary Figure S7). Importantly, resident, free-flying mallards in DHPs are not shown to be a source of new viruses among local waterfowl. Locally, however, viruses do move between DHPs. In particular, DHP_1_ was found to be a potential source of viruses that spread to other DHPs, which may relate to it having a larger waterbody that may attract birds and facilitate viral exchange. While likely related to the depletion of birds during hunting season, our study therefore invites further questions, particularly why IAVs are not being maintained long-term in DHPs. Is there a threshold population size for a particular DHP? Would a meta-population model of multiple DHPs inter-connected by more frequent movements of resident, free-flying birds between DHPs be able to sustain IAVs year-round? What is the role of the duck immune response to different HAs in inhibiting year-round IAV circulation? Furthermore, how do the seasonal movements of different species of wild birds affect the population dynamics of IAV in DHPs? The timing of IAV infection in DHPs could not be adequately explored in our study design; the absence of year-round sampling from the ducks hindered us as it has other investigators [39]. Additionally, the resolution of our study was limited by the amount public influenza surveillance data for wild birds in the region that could be used for comparison. Overall, it is useful for public health purposes to determine that DHPs are not accelerating the evolution of IAVs in USA avian populations. Additionally, DHPs may serve as convenient sentinel sites, since IAV positivity is amplified in DHPs compared to the number of positives sampled in wild birds in the Atlantic flyway. In the future, DHPs may provide a useful model system for studying IAV dynamics in a non-migratory, intensively monitored avian population.

## Figures and Tables

**Table 1:** Number of influenza A virus isolates/number of samples collected (%) annually in each duck hunting preserve (DHP).

	DHP_1_	DHP_2_	DHP_3_	DHP_4_	DHP_5_	DHP_6_	DHP_7_
2003	33/553 (6.0)	-	102/196 (78.6)	0/90 (0)	-	-	-
2004	32/452 (7.1)	-	-	6/146 (4.1)	-	-	-
2005	22/600 (3.4)	9/148 (6.1)	-	0/165 (0)	15/78 (19.2)	-	-
2006	23/711 (3.2)	0/115 (0)	-	0/282 (0)	48/169 (28.4)	2/70 (2.9)	5/103 (4.9)
2007	18/618 (2.9)	4/106 (3.8)	-	3/347 (0.9)	26/279 (9.3)	1/116 (0.8)	9/54 (16.7)
2008	18/524 (3.4)	42/100 (42)	-	0/258 (0)	2/206 (1.0)	1/71 (1.4)	8/98 (8.2)
2009	50/501 (10.0)	11/100 (11.0)	-	3/200 (1.5)	26/250 (10.4)	4/120 (3.3)	1/12 (8.3)
2010	29/660 (4.4)	20/100 (20.0)	-	8/255 (3.1)	0/100 (0)	1/75 (1.3)	-
2011	26/646 (4.0)	24/150 (16.0)	-	3/396 (0.8)	0/150 (0)	2/114 (1.8)	-
2012	33/572 (5.8)	0/100 (0)	-	7/354 (2.0)	28/130 (21.5)	4/118 (3.4)	-
2013	27/302 (8.9)	0/61 (0)	-	0/220 (0)	5/120 (4.2)	2/53 (3.77)	-
Total	311/6139(5.1)	110/980(11.2)	102/196 (78.6)	30/2713(1.1)	150/1482(10.1)	17/737(2.3)	23/267 (8.6)

**Table 2:** Number of complete viral genomes sequenced annually from each duck hunting preserve (DHP).

	DHP_1_	DHP_2_	DHP_3_	DHP_4_	DHP_5_	DHP_6_	DHP_7_
2003	5	-	2	-	-	-	-
2004	1	-	-	2	-	-	-
2005	8	6	-	3	2	-	-
2006	13	-	-	1	8	2	3
2007	11	2	-	1	2	-	2
2008	2	2	-	-	2	-	2
2009	6	2	-	2	5	3	-
2010	5	4	-	4	-	1	-
2011	7	2	-	-	-	2	-
2012	4	-	-	2	2	3	-
2003	8	-	-	-	-	2	-
Total	70	18	2	15	21	13	7

## Data Availability

The data supporting the results are available at https://figshare.com/s/fd57056937579e32dc89.

## Supplementary Materials

The following are available online at https://www.mdpi.com/2076-2607/9/1/40/s1, Supplementary Figure S1: Duck hunting preserves and poultry houses in Delmarva, Supplementary Figure S2: IAV HA subtypes circulating annually in Maryland duck hunting preserves and US, Supplementary Figure S3: Combinations of IAV HA subtypes circulating annually in Maryland duck hunting preserves, Atlantic flyway and US, Supplementary Figure S4: Genetic diversity of IAVs circulating in US Anatidae, US Neoaves and Maryland duck hunting preserves, Supplementary Figure S5: Viral introduction into Maryland duck hunting preserves, Supplementary Figure S6: Viral dissemination of IAV among US regions, habitat and duck hunting preserves, Supplementary Figure S7: Viral diversity in wild birds, poultry and Maryland duck hunting preserves. Supplementary Table S1: List of influenza A virus strains and GenBank accession numbers of the viral gene segments used in the present study, Supplementary Table S2: Percentage of Markov jump counts for the Flyway trait, Supplementary Table S3: Percentage of Markov jump counts for the Region trait, Supplementary Table S4: Percentage of Markov jump counts for the Habitat trait, Supplementary Table S5: Percentage of Markov jump counts for the Taxonomy trait, Supplementary Table S6: Percentage of Markov jump counts for the trait regarding the number of introductions per year.
