# Supplementary material for: The Evolutionary Dynamics of Influenza A Viruses Circulating in Mallards in Duck Hunting Preserves in Maryland, USA
Source: Microorganisms. 2020 Dec 25;9(1):40. doi: 10.3390/microorganisms9010040 (PMC7823399; doi:10.3390/microorganisms9010040)
Supplement: Supplementary file 1 [file microorganisms-09-00040-s001.pdf]

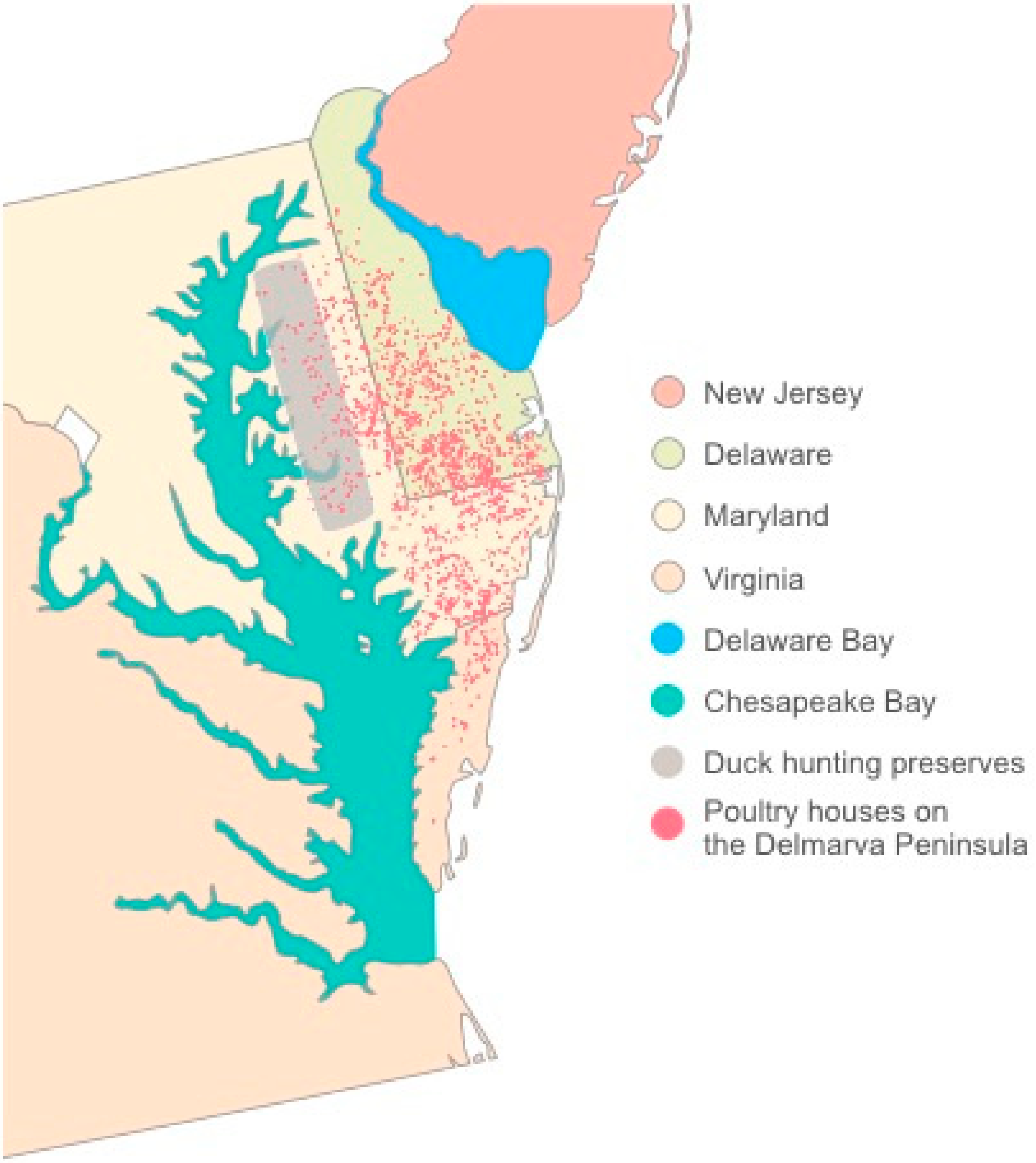

Supplementary Figure 1. Duck hunting preserves and poultry houses in Delmarva. Map depicting the Delmarva peninsula, the Chesapeake and Delaware bays (teal and blue, respectively), the area where the duck hunting preserves are located (gray), as well as poultry houses (red dots) identified using 2016 and 2017 aerial imagery from the U.S. Department of Agriculture's National Aerial Imagery Program (NAIP).

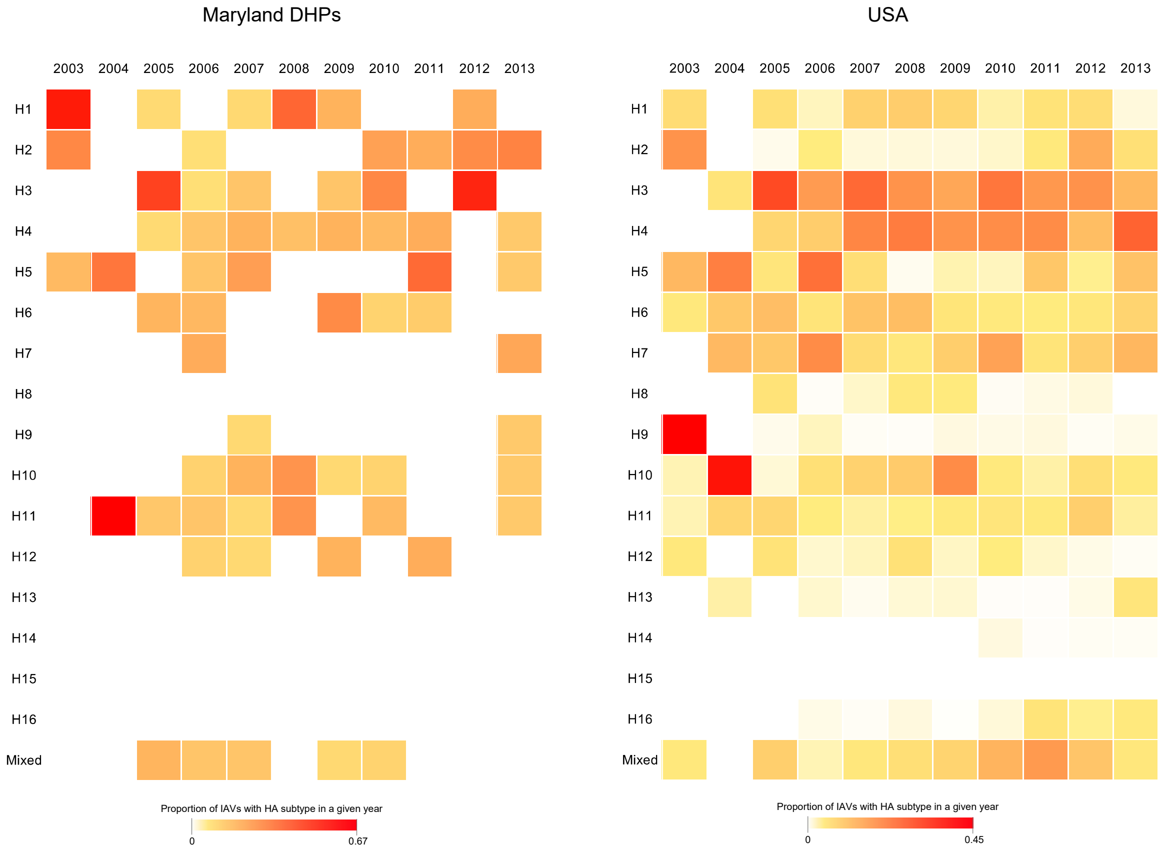

Supplementary Figure 2. IAV HA subtypes circulating annually in Maryland duck hunting preserves and US. Heatmap depicts proportion of IAVs circulating in a given year in the duck hunting preserves (white = 0; red = 67%) and entire US (white = 0; red = 45%).

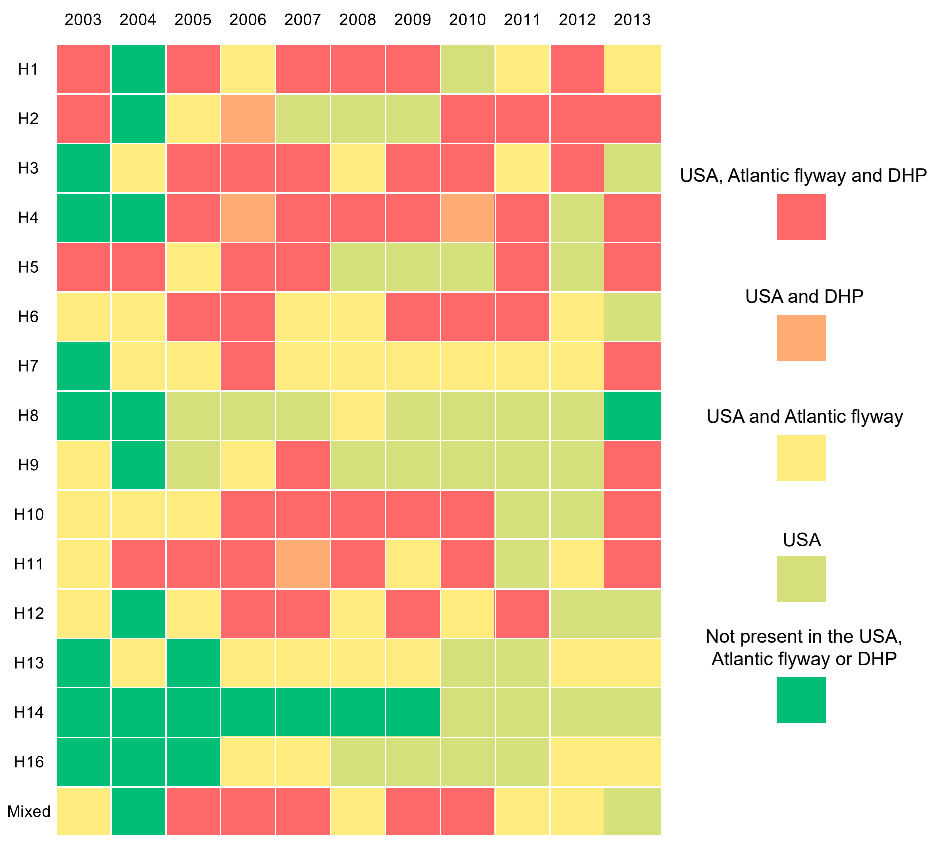

Supplementary Figure 3. Combinations of IAV HA subtypes circulating annually in Maryland duck hunting preserves, Atlantic flyway and US. Color depict whether the HA subtype was identified in the duck hunting preserves (DHPs), Atlantic flyways, entire US or a combination of these locations during the 11 years of DHPs' sampling, excluding viruses isolated in poultry. No IAV HA subtype was ever identified only in a DHP.

AIV Subtypes		
Anatidae	***Neoaves***	**DHPs**
H1N1	H1N1	H1N1
H1N2	H1N2	
H1N3		
H1N5	H1N5	
H1N6		
	H1N7	
H1N8	H1N8	H1N8
H1N9	H1N9	
H2N1		H2N1
H2N2		H2N2
H2N3		H2N3
H2N4		
H2N5		
H2N6	H2N6	
H2N7		
H2N8		
H2N9		
H3N1		
H3N2	H3N2	H3N2
H3N3		
H3N4		
H3N5		
H3N6	H3N6	H3N6
H3N7	H3N7	
H3N8	H3N8	H3N8
H3N9	H3N9	
H4N1		
H4N2		H4N2
H4N3		
H4N4		
H4N5		
H4N6	H4N6	H4N6
H4N7	H4N7	
H4N8	H4N8	H4N8
H4N9		
H5N1	H5N1	H5N1
H5N2	H5N2	H5N2
H5N3		
H5N4		
H5N5		
H5N6		
H5N7	H5N7	
H5N8		H5N8
H5N9		H5N9
H6N1	H6N1	H6N1
H6N2	H6N2	H6N2
H6N3		
H6N4		
H6N5		H6N5
H6N6	H6N6	
H6N7	H6N7	
H6N8	H6N8	H6N8
H6N9		
H7N1		
H7N2	H7N2	
H7N3	H7N3	H7N3
H7N4	H7N4	H7N4
H7N5		
H7N6		
H7N7	H7N7	
H7N8		
H7N9		
H8N1		
H8N2		
H8N3		
H8N4	H8N4	
H8N8		
	H9N1	H9N1
H9N2	H9N2	H9N2
	H9N5	
	H9N7	
	H9N8	
	H9N9	
H10N1	H10N1	
H10N2	H10N2	
H10N3	H10N3	
H10N4		H10N4
H10N5		
H10N6	H10N6	H10N6
H10N7	H10N7	H10N7
H10N8	H10N8	
H10N9		H10N9
H11N1	H11N1	
H11N2	H11N2	
H11N3		
H11N5	H11N5	
H11N6	H11N6	
H11N8	H11N8	
H11N9	H11N9	H11N9
	H12N1	
H12N2		
H12N4	H12N4	
H12N5	H12N5	H12N5
H12N8		H12N8
		H12N9
	H13N2	
	H13N3	
	H13N6	
	H13N8	
H13N9	H13N9	
H14N2		
H14N5		
H14N6		
H14N8		
	H16N3	
mixed	mixed	mixed

Legend
Anatidae
Neoaves
Anatidae and *Neoaves*

Supplementary Figure 4. Genetic diversity of IAVs circulating in US *Anatidae*, US *Neoaves* and Maryland duck hunting preserves. We identified 93, 56 and 32 HA-NA subtypes circulating in the Anatidae family, the Neoaves superorder and Maryland duck hunting preserves (DHPs), respectively, during 2003-2013. Color in the DHPs column indicates whether the subtype was also isolated in *Anatidae* (blue), *Neoaves* (purple) or both taxonomic groups (green).

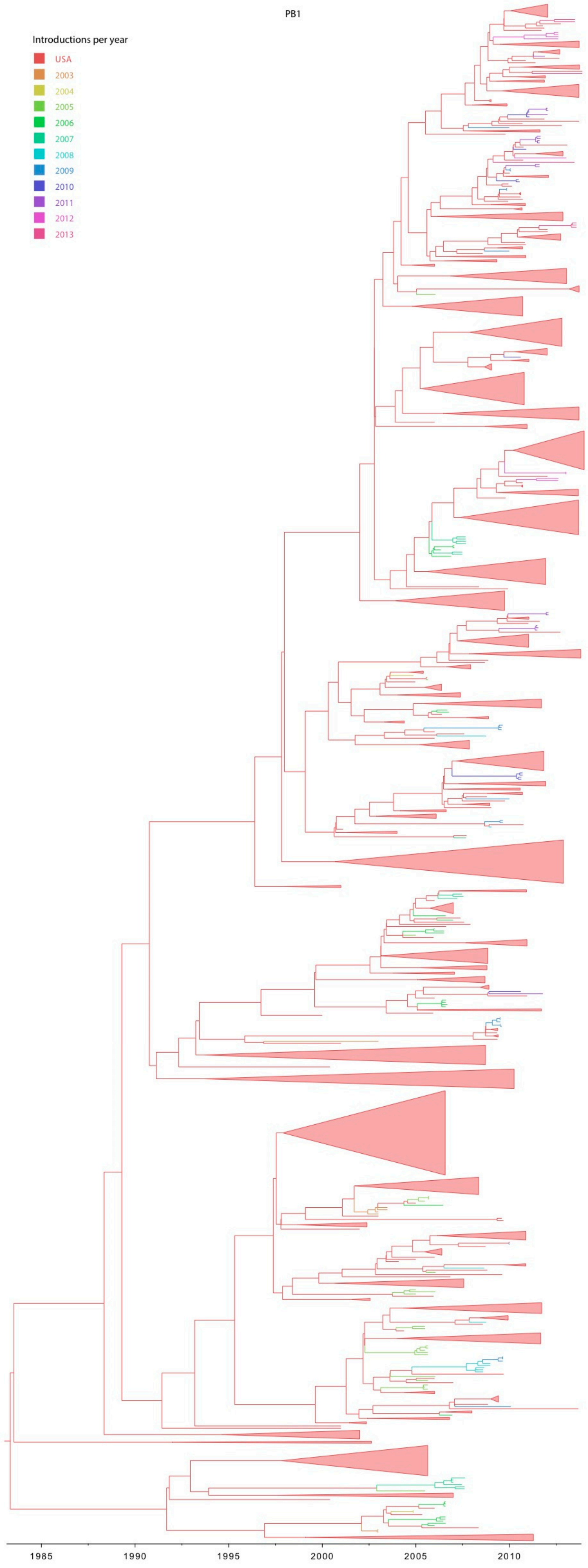
PB1
Introductions per year
USA
2003
2004
2005
2006
2007
2008
2009
2010
2011
2012
2013
1985
1990
1995
2000
2005
2010

Supplementary Figure 5. Viral introduction into Maryland duck hunting preserves. Maximum clade credibility trees depicting USA IAV introductions into Maryland duck hunting preserves from 2003-2013 for all internal segments except PB2 (Figure 5).

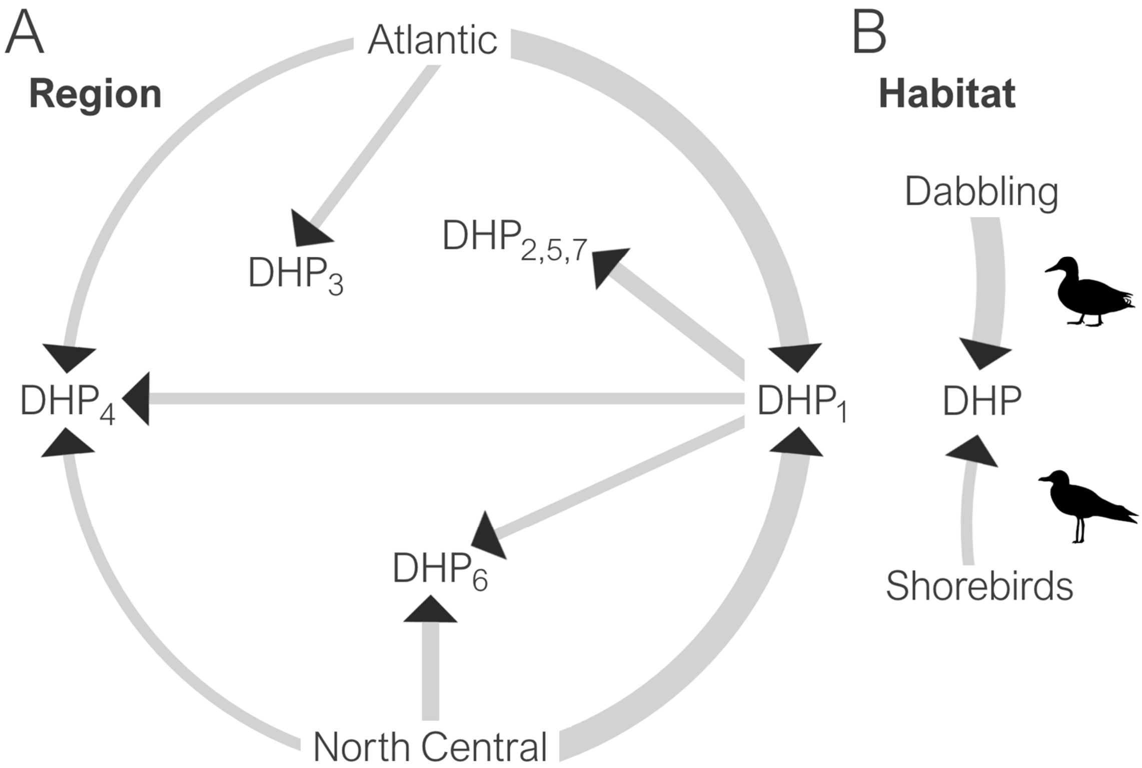

Supplementary Figure 6. Viral dissemination of IAV among US regions, habitat and duck hunting preserves. Representation of Markov Jumps (Supplementary Tables) for US regions (A) and habitat (B). Arrow width represents relative proportion of viruses seeded into the duck hunting preserves (thin – low proportion of viral jumps to thick – high proportion of viral jumps).

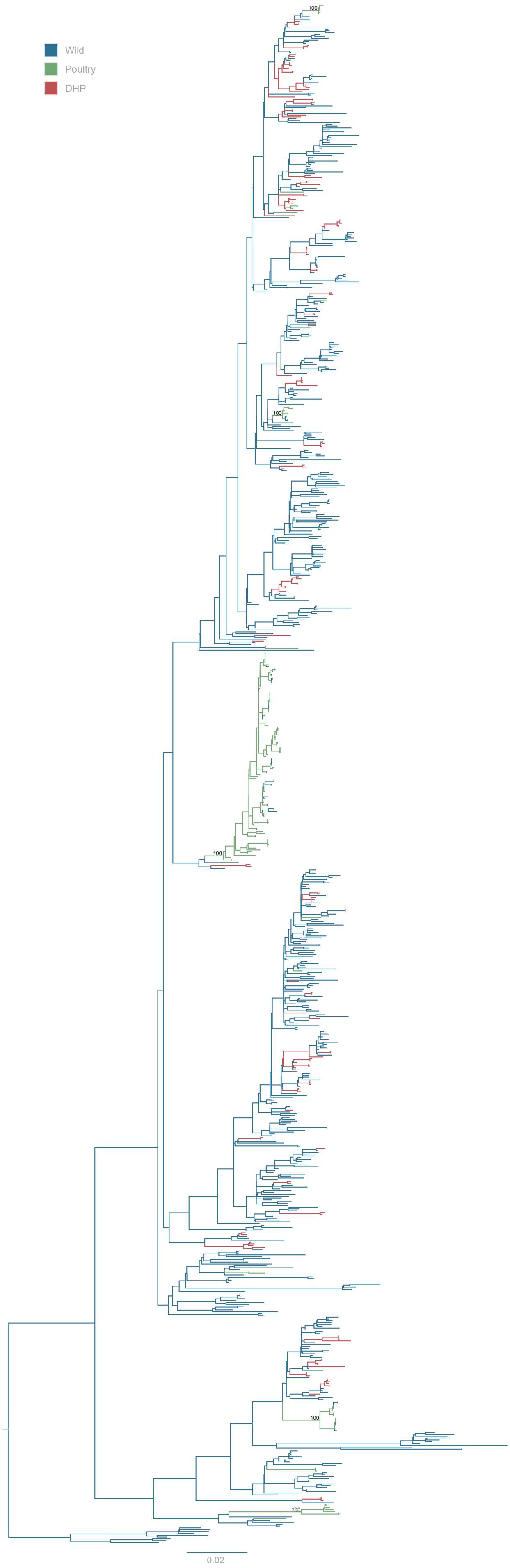
Wild
Poultry
DHP
100
100
100
100
100
0.02

Supplementary Figure 7. Viral diversity in wild birds, poultry and Maryland duck hunting preserves. Maximum likelihood tree depicts USA IAV diversity circulating in wild birds (blue), poultry (green) and Maryland duck hunting preserves (red) from 2003-2013 for the PB2 gene segment. Numbers on the nodes represent bootstrap support values from 1000 replicates for large poultry clusters.

Supplementary Table 1: List of influenza A virus strains and GenBank accession numbers of the viral gene segments used in the present study.

Strain Name	1	2	3	4	5	6	7	8
A/American black duck/Delaware/A00870108/2010(H7N3)	KU289745	KU289744	KU289743		KU289741		KU289739	KU289742
A/American black duck/Illinois/08OS2688/2008(H5N2)	CY079459	CY079458	CY079457		CY079455		CY079453	CY079456
A/American black duck/Maine/44411/532/2008(H3N8)	KP636475	KP636476	KP636477		KP636479		KP636481	KP636482
A/American black duck/Maryland/07OS2482/2007(H6N8)	CY190594	CY190593	CY190592		CY190590		CY190588	CY190591
A/American black duck/Maryland/13OS2956/2013(H11N9)	CY191282	CY191281	CY191280		CY191278		CY191276	CY191279
A/American black duck/New Brunswick/00322/2010(H3N8)	CY138655	CY138654	CY138653		CY138651		CY138649	CY138652
A/American black duck/New Brunswick/00464/2010(H4N6)	CY138052	CY138051	CY138050		CY138048		CY138046	CY138049
A/American black duck/New Brunswick/00867/2010(H5N2)	CY139334	CY139333	CY139332		CY139330		CY139328	CY139331
A/American black duck/New Brunswick/00924/2010(H10N7)	CY139428	CY139427	CY139426		CY139424		CY139422	CY139425
A/American black duck/New Brunswick/00986/2010(H4N6)	CY139512	CY139511	CY139510		CY139508		CY139506	CY139509
A/American black duck/New Brunswick/02497/2007(H3N8)	CY129260	CY129259	CY129258		CY129256		CY129254	CY129257
A/American black duck/North Carolina/1321373/2004(H3N2)	CY144578	CY144577	CY144576		CY144574		CY144572	CY144575
A/american black duck/Ohio/1823/2005(H11N9)	CY021252	CY021251	CY021250		CY021248		CY021246	CY021249
A/American black duck/Prince Edward Island/02662/2007(H4N6)	CY125645	CY125644	CY125643		CY125641		CY125639	CY125642
A/American black duck/Wisconsin/10OS3949/2010(H7N8)	CY133281	CY133280	CY133279		CY133277		CY133275	CY133278
A/American black duck/Wisconsin/11OS3191/2011(H6N1)	CY166904	CY166903	CY166902		CY166900		CY166898	CY166901
A/American black duck/Wisconsin/2542/2009(H4N2)	CY096985	CY096984	CY096983		CY096981		CY096979	CY096982
A/American coot/Illinois/3405/2009(H10N3)	CY097166	CY097165	CY097164		CY097162		CY097160	CY097163
A/American coot/Mississippi/09OS615/2009(H10N3)	CY079411	CY079410	CY079409		CY079407		CY079405	CY079408
A/American coot/Oregon/20589-007/2007(H3N8)	CY076172	CY076171	CY076170		CY076168		CY076166	CY076169
A/American green winged teal/Arizona/A00115994/2009(H7N3)	KU289753	KU289752	KU289751		KU289749		KU289747	KU289750
A/American green winged teal/Colorado/A00551331/2007(H7N3)	KU289769	KU289768	KU289767		KU289765		KU289763	KU289766
A/American green winged teal/Colorado/A00660616/2008(H7N3)	KU289777	KU289776	KU289775		KU289773		KU289771	KU289774
A/American green winged teal/Texas/A00586649/2009(H7N3)	KU289801	KU289800	KU289799		KU289797		KU289795	KU289798
A/American green winged teal/Texas/A00604814/2009(H7N3)	KU289833	KU289832	KU289831		KU289829		KU289827	KU289830
A/American green winged teal/Utah/A00461136/2009(H7N1)	KU289849	KU289848	KU289847		KU289845		KU289843	KU289846
A/American green winged teal/Utah/A00468772/2009(H7N7)	KU289857	KU289856	KU289855		KU289853		KU289851	KU289854
A/American green winged teal/Utah/A00614935/2009(H7N3)	KU289865	KU289864	KU289863		KU289861		KU289859	KU289862
A/American green winged teal/Utah/A00833077/2009(H7N3)	KU289886	KU289885	KU289884		KU289882		KU289880	KU289883
A/American green winged teal/Wyoming/A00230796/2008(H7N3)	KU289894	KU289893	KU289892		KU289890		KU289888	KU289891
A/American green-winged teal/Alaska/103773/2008(H3N8)	KX714367	KX714368	KX714369		KX714371		KX714373	KX714374
A/American green-winged teal/Alaska/103777/2008(H4N6)	KX714375	KX714376	KX714377		KX714379		KX714381	KX714382
A/American green-winged teal/Alaska/137896/2009(H3N8)	KX714439	KX714440	KX714441		KX714443		KX714445	KX714446
A/American green-winged teal/Alaska/137916/2009(H3N8)	KX714447	KX714448	KX714449		KX714451		KX714453	KX714454
A/American green-winged teal/Alaska/14164/2006(H3N6)	KX714319	KX714320	KX714321		KX714323		KX714325	KX714326
A/American green-winged teal/California/44287-066/2007(H11N9)	CY076348	CY076347	CY076346		CY076344		CY076342	CY076345
A/American green-winged teal/Illinois/10OS1551/2010(H4N6)	CY132900	CY132899	CY132898		CY132896		CY132894	CY132897
A/American green-winged teal/Illinois/10OS1598/2010(H4N8)	CY132924	CY132923	CY132922		CY132920		CY132918	CY132921
A/American green-winged teal/Illinois/10OS3329/2010(H7N7)	CY132980	CY132979	CY132978		CY132976		CY132974	CY132977
A/American green-winged teal/Illinois/10OS3343/2010(H2N3)	CY132996	CY132995	CY132994		CY132992		CY132990	CY132993
A/American green-winged teal/Illinois/10OS4014/2010(H7N3)	CY133044	CY133043	CY133042		CY133040		CY133038	CY133041

A/American green-winged teal/Illinois/11OS4814/2011(H3N8)	CY166393	CY166392	CY166391	CY166389	CY166387	CY166390
A/American green-winged teal/Illinois/12OS5103/2012(H3N8)	CY186682	CY186681	CY186680	CY186678	CY186676	CY186679
A/American green-winged teal/Illinois/2479/2009(H2N3)	CY097210	CY097209	CY097208	CY097206	CY097204	CY097207
A/American green-winged teal/Illinois/3054/2009(H1N2)	CY097202	CY097201	CY097200	CY097198	CY097196	CY097199
A/American green-winged teal/Interior Alaska/10BM08222R0/2010(H3N8)	CY143563	CY143562	CY143561	CY143559	CY143557	CY143560
A/American green-winged teal/Interior Alaska/11BM00293/2011(H10N5)	CY196024	CY196023	CY196022	CY196020	CY196018	CY196021
A/American green-winged teal/Interior Alaska/4/2007(H3N8)	CY039799	CY039800	CY039801	CY039803	CY039805	CY039806
A/American green-winged teal/Interior Alaska/7MP2225/2007(H3N8)	CY047034	CY047033	CY047032	CY047030	CY047028	CY047031
A/American green-winged teal/Interior Alaska/9BM12318R0/2009(H3N8)	CY143177	CY143176	CY143175	CY143173	CY143171	CY143174
A/American green-winged teal/Interior Alaska/9BM6410R0/2009(H3N8)	CY142633	CY142632	CY142631	CY142629	CY142627	CY142630
A/American green-winged teal/Maryland/06MD700/2006(H6N8)	CY190034	CY190033	CY190032	CY190030	CY190028	CY190031
A/American green-winged teal/Mississippi/09OS046/2009(H7N7)	CY079315	CY079314	CY079313	CY079311	CY079309	CY079312
A/American green-winged teal/Mississippi/11OS250/2011(H7N3)	CY133676	CY133675	CY133674	CY133672	CY133670	CY133673
A/American green-winged teal/Mississippi/11OS255/2011(H7N7)	CY133684	CY133683	CY133682	CY133680	CY133678	CY133681
A/American green-winged teal/Mississippi/11OS256/2011(H1N1)	CY133692	CY133691	CY133690	CY133688	CY133686	CY133689
A/American green-winged teal/Mississippi/11OS257/2011(H11N9)	CY133700	CY133699	CY133698	CY133696	CY133694	CY133697
A/American green-winged teal/Mississippi/11OS5869/2011(H4N6)	CY166775	CY166774	CY166773	CY166771	CY166769	CY166772
A/American green-winged teal/Mississippi/11OS5903/2011(H4N6)	CY166839	CY166838	CY166837	CY166835	CY166833	CY166836
A/American green-winged teal/Mississippi/11OS90/2011(H11N9)	CY133648	CY133647	CY133646	CY133644	CY133642	CY133645
A/American green-winged teal/Mississippi/12OS191/2012(H5N3)	CY167182	CY167181	CY167180	CY167178	CY167176	CY167179
A/American green-winged teal/Mississippi/12OS397/2012(H7N7)	CY167239	CY167238	CY167237	CY167235	CY167233	CY167236
A/American green-winged teal/Mississippi/12OS405/2012(H10N7)	CY167247	CY167246	CY167245	CY167243	CY167241	CY167244
A/American green-winged teal/Mississippi/12OS5061/2012(H5N2)	CY186795	CY186794	CY186793	CY186791	CY186789	CY186792
A/American green-winged teal/Mississippi/285/2010(H3N8)	CY097685	CY097684	CY097683	CY097681	CY097679	CY097682
A/American green-winged teal/Mississippi/300/2010(H11N9)	CY097693	CY097692	CY097691	CY097689	CY097687	CY097690
A/American green-winged teal/Ohio/12OS5538/2012(H7N3)	CY187036	CY187035	CY187034	CY187032	CY187030	CY187033
A/American green-winged teal/Ohio/13OS1794/2013(H6N1)	KJ568134	KJ568135	KJ568136	KJ568138	KJ568140	KJ568141
A/American green-winged teal/Ohio/13OS1840/2013(H6N8)	KJ567910	KJ567911	KJ567912	KJ567914	KJ567916	KJ567917
A/American green-winged teal/Ohio/13OS1844/2013(H3N8)	KJ568246	KJ568247	KJ568248	KJ568250	KJ568252	KJ568253
A/American green-winged teal/Ohio/13OS1861/2013(H3N8)	KJ568158	KJ568159	KJ568160	KJ568162	KJ568164	KJ568165
A/American green-winged teal/Ohio/13OS1864/2013(H4N8)	KJ567926	KJ567927	KJ567928	KJ567930	KJ567932	KJ567933
A/American green-winged teal/Ohio/13OS2070/2013(H6N7)	KJ568374	KJ568375	KJ568376	KJ568378	KJ568380	KJ568381
A/American green-winged teal/Ohio/13OS2086/2013(H6N8)	KJ568302	KJ568303	KJ568304	KJ568306	KJ568308	KJ568309
A/American green-winged teal/Ohio/13OS2090/2013(H6N2)	KJ568142	KJ568143	KJ568144	KJ568146	KJ568148	KJ568149
A/American green-winged teal/Oregon/44336-183/2007(H3N8)	CY076484	CY076483	CY076482	CY076480	CY076478	CY076481
A/American green-winged teal/Texas/AI09-4396/2009(H3N9)	CY140960	CY140959	CY140958	CY140956	CY140954	CY140957
A/American green-winged teal/Wisconsin/08OS2270/2008(H3N8)	CY079427	CY079426	CY079425	CY079423	CY079421	CY079424
A/American green-winged teal/Wisconsin/08OS2292/2008(H3N2)	CY079347	CY079346	CY079345	CY079343	CY079341	CY079344
A/American green-winged teal/Wisconsin/10OS2847/2010(H1N1)	CY133092	CY133091	CY133090	CY133088	CY133086	CY133089
A/American green-winged teal/Wisconsin/11OS3053/2011(H6N1)	CY166329	CY166328	CY166327	CY166325	CY166323	CY166326
A/American green-winged teal/Wisconsin/11OS3425/2011(H12N5)	CY166636	CY166635	CY166634	CY166632	CY166630	CY166633
A/American green-winged teal/Wisconsin/12OS2994/2012(H1N1)	CY187159	CY187158	CY187157	CY187155	CY187153	CY187156
A/American green-winged teal/Wisconsin/12OS2997/2012(H4N6)	CY187167	CY187166	CY187165	CY187163	CY187161	CY187164
A/American green-winged teal/Wisconsin/12OS3031/2012(H11N9)	CY187134	CY187133	CY187132	CY187130	CY187128	CY187131

A/American green-winged teal/Wisconsin/12OS4536/2012(H11N9)	CY187142	CY187141	CY187140	CY187138	CY187136	CY187139
A/American green-winged teal/Wisconsin/13OS3465/2013(H6N8)	KJ568118	KJ568119	KJ568120	KJ568122	KJ568124	KJ568125
A/American widgeon/Alaska/7MP1061/2007(H3N8)	CY043983	CY043982	CY043981	CY043979	CY043977	CY043980
A/American widgeon/Interior Alaska/7MP1707/2007(H3N8)	CY077363	CY077362	CY077361	CY077359	CY077357	CY077360
A/American wigeon/California/2423/2010(H6N1)	CY133884	CY133883	CY133882	CY133880	CY133878	CY133881
A/American wigeon/California/6610/2008(H12N5)	CY093805	CY093804	CY093803	CY093801	CY093799	CY093802
A/American wigeon/California/8352/2008(H12N5)	CY094388	CY094387	CY094386	CY094384	CY094382	CY094385
A/American wigeon/California/HKWF1174/2007(H6N1)	CY033353	CY033354	CY033355	CY033357	CY033359	CY033360
A/American wigeon/Interior Alaska/11CH00036/2011(H4N6)	CY196378	CY196377	CY196376	CY196374	CY196372	CY196375
A/American wigeon/Iowa/463998/2006(H5N2)	GQ923204	GQ923203	GQ923202	GQ923200	GQ923198	GQ923201
A/American wigeon/Louisiana/Sg-01032/2008(H6N2)	CY140550	CY140549	CY140548	CY140546	CY140544	CY140547
A/American wigeon/Minnesota/Sg-01067/2008(H3N8)	CY140630	CY140629	CY140628	CY140626	CY140624	CY140627
A/black duck/Maryland/415/2001(H7N3)	CY020892	CY020891	CY020890	CY020888	CY020886	CY020889
A/black duck/Maryland/834/2002(H4N8)	CY020772	CY020771	CY020770	CY020768	CY020766	CY020769
A/black scoter/Maine/276/2011(H10N7)	CY149579	CY149578	CY149577	CY149575	CY149573	CY149576
A/black scoter/New Brunswick/00002/2009(H3N8)	CY125348	CY125347	CY125346	CY125344	CY125342	CY125345
A/black-bellied whistling duck/Colombia/1/2011(H5N2)	CY207196	CY207195	CY207194	CY207192	CY207190	CY207193
A/black-necked stilt/Chile/1/2013(H11N9)	CY207212	CY207211	CY207210	CY207208	CY207206	CY207209
A/blue winged teal/Minnesota/A00137660/2009(H7N3)	KU289918	KU289917	KU289916	KU289914	KU289912	KU289915
A/blue winged teal/Missouri/A00624484/2008(H7N3)	KU289934	KU289933	KU289932	KU289930	KU289928	KU289931
A/blue winged teal/South Dakota/A00772794/2009(H7N7)	KU289942	KU289941	KU289940	KU289938	KU289936	KU289939
A/blue winged teal/TX/75/2002(H1N3)	FJ357052	FJ357051	FJ357050	FJ357048	FJ357046	FJ357049
A/blue-winged teal/Canada/3264/2011(H3N8)	CY149707	CY149706	CY149705	CY149703	CY149701	CY149704
A/blue-winged teal/Guatemala/CIP049-09/2010(H5N3)	CY096717	CY096718	CY096719	CY096721	CY096723	CY096724
A/blue-winged teal/Guatemala/CIP049H098-32/2010(H5N3)	KX365074	KX365068	KX365065	KX365056	KX365055	KX365057
A/blue-winged teal/Guatemala/CIP049H102-25/2011(H3N2)	KX960415	KX986865	KX960383	KX960389	KX960485	KX960490
A/blue-winged teal/Guatemala/CIP049H102-29/2011(H3N2)	KX960460	KX986871	KX960483	KX960492	KX960376	KX960424
A/blue-winged teal/Guatemala/CIP049H103-13/2011 (H4N3)	KX365063	KX365061	KX365077	KX365072	KX365066	KX365058
A/blue-winged teal/Guatemala/CIP049H103-30/2011(H4N3)	KX960414	KX986866	KX960471	KX960408	KX960368	KX960491
A/blue-winged teal/Guatemala/CIP049H105-32/2011(H1N3)	KX960448	KX986867	KX960454	KX960481	KX960395	KX960388
A/blue-winged teal/Guatemala/CIP049H108-53/2012(H4N2)	KX960430	KX986869	KX960371	KX960441	KX960394	KX960449
A/blue-winged teal/Guatemala/CIP049H109-86/2012(H4N2)	KX960431	KX986860	KX960419	KX960453	KX960398	KX960372
A/blue-winged teal/Iowa/10OS2624/2010(H3N2)	CY133608	CY133607	CY133606	CY133604	CY133602	CY133605
A/blue-winged teal/Iowa/10OS2649/2010(H3N6)	CY133624	CY133623	CY133622	CY133620	CY133618	CY133621
A/blue-winged teal/Iowa/13OS2316/2013(H7N3)	KJ568342	KJ568343	KJ568344	KJ568346	KJ568348	KJ568349
A/blue-winged teal/Iowa/13OS2349/2013(H4N8)	KJ568150	KJ568151	KJ568152	KJ568154	KJ568156	KJ568157
A/blue-winged teal/Iowa/44555-551/2011(H3N2)	CY166281	CY166280	CY166279	CY166277	CY166275	CY166278
A/blue-winged teal/Kansas/ND0006685/2013(H3N8)	KM374031	KM374032	KM374033	KM374035	KM374037	KM374038
A/blue-winged teal/Louisiana/AI10-3543/2010(H4N8)	CY205604	CY205603	CY205602	CY205600	CY205598	CY205601
A/blue-winged teal/Louisiana/AI10-3613/2010(H4N6)	CY205621	CY205620	CY205619	CY205617	CY205615	CY205618
A/blue-winged teal/Louisiana/AI12-4445/2012(H4N6)	CY206283	CY206282	CY206281	CY206279	CY206277	CY206280
A/blue-winged teal/Louisiana/AI13-3483/2013(H3N8)	CY206340	CY206339	CY206338	CY206336	CY206334	CY206337
A/blue-winged Teal/Minnesota/AI09-2977/2009(H4N8)	CY140768	CY140767	CY140766	CY140764	CY140762	CY140765
A/blue-winged teal/Minnesota/Sg-00029/2007(H4N6)	CY063872	CY063871	CY063870	CY063868	CY063866	CY063869

A/blue-winged teal/Minnesota/Sg-00037/2007(H3N8)	CY063936	CY063935	CY063934		CY063932		CY063930	CY063933
A/blue-winged teal/Minnesota/Sg-00799/2008(H4N6)	CY140211	CY140210	CY140209		CY140207		CY140205	CY140208
A/blue-winged teal/Missouri/10MO003/2010(H4N6)	CY133744	CY133743	CY133742		CY133740		CY133738	CY133741
A/blue-winged teal/Missouri/10MO013/2010(H3N1)	CY133760	CY133759	CY133758		CY133756		CY133754	CY133757
A/blue-winged teal/Missouri/12OS2519/2012(H4N8)	CY186763	CY186762	CY186761		CY186759		CY186757	CY186760
A/blue-winged Teal/North Dakota/AI09-2912/2009(H6N1)	CY140760	CY140759	CY140758		CY140756		CY140754	CY140757
A/blue-winged Teal/North Dakota/AI09-3131/2009(H4N6)	CY140803	CY140802	CY140801		CY140799		CY140797	CY140800
A/blue-winged teal/North Dakota/Sg-00706/2008(H4N6)	CY140119	CY140118	CY140117		CY140115		CY140113	CY140116
A/blue-winged teal/North Dakota/Sg-00719/2008(H3N8)	CY140127	CY140126	CY140125		CY140123		CY140121	CY140124
A/blue-winged teal/North Dakota/Sg-00730/2008(H10N7)	CY140135	CY140134	CY140133		CY140131		CY140129	CY140132
A/blue-winged teal/North Dakota/Sg-00740/2008(H3N8)	CY140151	CY140150	CY140149		CY140147		CY140145	CY140148
A/blue-winged teal/North Dakota/Sg-00750/2008(H4N6)	CY140171	CY140170	CY140169		CY140167		CY140165	CY140168
A/blue-winged teal/Ohio/12OS2244/2012(H4N6)	CY186902	CY186901	CY186900		CY186898		CY186896	CY186899
A/blue-winged teal/Ohio/12OS2321/2012(H4N6)	CY186803	CY186802	CY186801		CY186799		CY186797	CY186800
A/blue-winged teal/Texas/AI09-4405/2009(H3N6)	CY140968	CY140967	CY140966		CY140964		CY140962	CY140965
A/blue-winged teal/Texas/AI10-3272/2010(H4N8)	CY205522	CY205521	CY205520		CY205518		CY205516	CY205519
A/blue-winged teal/Texas/AI10-3336/2010(H4N8)	CY205556	CY205555	CY205554		CY205552		CY205550	CY205553
A/blue-winged teal/Texas/AI10-3382/2010(H4N3)	CY205572	CY205571	CY205570		CY205568		CY205566	CY205569
A/blue-winged teal/Texas/AI10-3744/2010(H3N8)	CY205637	CY205636	CY205635		CY205633		CY205631	CY205634
A/blue-winged teal/Texas/AI10-3766/2010(H3N8)	CY205645	CY205644	CY205643		CY205641		CY205639	CY205642
A/blue-winged teal/Texas/AI10-4465/2010(H4N8)	CY205767	CY205766	CY205765		CY205763		CY205761	CY205764
A/blue-winged teal/Texas/AI10-4494/2010(H4N6)	CY205783	CY205782	CY205781		CY205779		CY205777	CY205780
A/blue-winged teal/Texas/AI11-3180/2011(H4N6)	CY205886	CY205885	CY205884		CY205882		CY205880	CY205883
A/blue-winged teal/Texas/AI11-3411/2011(H4N6)	CY205994	CY205993	CY205992		CY205990		CY205988	CY205991
A/blue-winged teal/Texas/AI11-3764/2011(H3N8)	CY206058	CY206057	CY206056		CY206054		CY206052	CY206055
A/blue-winged teal/Texas/AI11-4849/2011(H4N8)	CY206146	CY206145	CY206144		CY206142		CY206140	CY206143
A/blue-winged teal/Texas/AI11-4865/2011(H3N6)	CY206170	CY206169	CY206168		CY206166		CY206164	CY206167
A/blue-winged teal/Texas/AI12-3834/2012(H3N6)	CY206259	CY206258	CY206257		CY206255		CY206253	CY206256
A/blue-winged teal/Texas/AI12-4448/2012(H3N6)	CY206291	CY206290	CY206289		CY206287		CY206285	CY206288
A/blue-winged teal/Texas/AI12-4566/2012(H11N9)	CY206315	CY206314	CY206313		CY206311		CY206309	CY206312
A/blue-winged teal/Texas/AI13-3557/2013(H4N6)	CY206364	CY206363	CY206362		CY206360		CY206358	CY206361
A/blue-winged teal/Texas/AI13-3650/2013(H4N8)	CY206453	CY206452	CY206451		CY206449		CY206447	CY206450
A/blue-winged teal/Texas/AI13-4085/2013(H4N8)	CY206543	CY206542	CY206541		CY206539		CY206537	CY206540
A/blue-winged teal/Texas/AI13-4151/2013(H4N6)	CY206578	CY206577	CY206576		CY206574		CY206572	CY206575
A/blue-winged teal/Texas/Sg-00074/2007(H4N8)	CY077708	CY077707	CY077706		CY077704		CY077702	CY077705
A/blue-winged teal/Texas/Sg-00078/2007(H3N8)	CY064224	CY064223	CY064222		CY064220		CY064218	CY064221
A/blue-winged teal/Texas/Sg-00081/2007(H4N6)	CY064248	CY064247	CY064246		CY064244		CY064242	CY064245
A/blue-winged teal/Texas/Sg-00188/2007(H4N8)	CY078312	CY078311	CY078310		CY078308		CY078306	CY078309
A/blue-winged teal/Wisconsin/10OS2862/2010(H3N2)	CY133108	CY133107	CY133106		CY133104		CY133102	CY133105
A/blue-winged teal/Wisconsin/11OS2657/2011(H4N6)	CY166484	CY166483	CY166482		CY166480		CY166478	CY166481
A/blue-winged teal/Wisconsin/2649/2009(H6N1)	CY097469	CY097468	CY097467		CY097465		CY097463	CY097466
A/bufflehead/Illinois/4016/2009(H4N8)	CY096977	CY096976	CY096975		CY096973		CY096971	CY096974
A/bufflehead/Maine/338/2011(H4N8)	CY149603	CY149602	CY149601		CY149599		CY149597	CY149600
A/bufflehead/Maine/340/2011(H6N8)	CY149611	CY149610	CY149609		CY149607		CY149605	CY149608

A/bufflehead/North Carolina/NC6412-125/2005(H4N8)	CY144666	CY144665	CY144664	CY144662	CY144660	CY144663
A/bufflehead/Virginia/A00120022/2008(H7N2)	KU289974	KU289973	KU289972	KU289970	KU289968	KU289971
A/bufflehead/Wisconsin/10OS3204/2010(H12N5)	CY133116	CY133115	CY133114	CY133112	CY133110	CY133113
A/call duck/Maryland/S698/2004(H11N3)	KJ361907	KJ361906	KJ361905	KJ361903	KJ361901	KJ361904
A/Canada goose/BC/3752/2007(H7N3)	EU500841	EU500842	EU500843	EU500845	EU500847	EU500848
A/Canada goose/Delaware Bay/34/2010(H6N1)	CY127920	CY127919	CY127918	CY127916	CY127914	CY127917
A/Canada goose/Wyoming/473197-12/2006(H5N2)	GQ923332	GQ923331	GQ923330	GQ923328	GQ923326	GQ923329
A/canvasback/Alberta/276/2005(H1N1)	CY137641	CY137640	CY137639	CY137637	CY137635	CY137638
A/chicken/Aguascalientes/IA13/2010(H5N2)	KM657856	KM657857	KM657858	KM657860	KM657862	KM657863
A/chicken/BC/CN-006/2004(H7N3)	KP055066	KP055067	KP055068	KP055070	KP055072	KP055073
A/chicken/CA/6643/2001(H6N2)	EU182258					
A/chicken/California/6643/01(H6N2)		AF457682	AF457683	AF457685	AF457687	AF457684
A/chicken/Coahuila/1433-15/2006(H5N2)	KM455942	KM455943	KM455944	KM455946	KM455948	KM455949
A/chicken/CT/260413-2/2003(H7N2)	EU743002	EU743001	EU743000	EU742998	EU742996	EU742999
A/chicken/Durango/1433-4/2005(H5N2)	KM455886	KM455887	KM455888	KM455890	KM455892	KM455893
A/chicken/Durango/1433-8/2005(H5N2)	KM455910	KM455911	KM455912	KM455914	KM455916	KM455917
A/chicken/Durango/1433-9/2006(H5N2)	KM455918	KM455919	KM455920	KM455922	KM455924	KM455925
A/chicken/FL/90348-4/2001(H7N2)	EU742895	EU742894	EU742893	EU742891	EU742889	EU742892
A/chicken/Guatemala/194573/2002(H5N2)	GU186564	GU186563	GU186562	GU186560	GU186558	GU186561
A/chicken/Hidalgo/1433-6/2005(H5N2)	KM455902	KM455903	KM455904	KM455906	KM455908	KM455909
A/chicken/Jalisco/CPA1/2012(H7N3)	JX465635	JX465634	JX465636	JX465633	JX465631	JX465632
A/chicken/MD/MinhMa/2004(H7N2)	HQ541726	HQ541725	HQ541724	HQ541723	HQ541721	HQ541722
A/chicken/Mexico/1433-13/2006(H5N2)	KM455934	KM455935	KM455936	KM455938	KM455940	KM455941
A/chicken/Mexico/1433-5/2005(H5N2)	KM455894	KM455895	KM455896	KM455898	KM455900	KM455901
A/chicken/New Jersey/251-4/2008(H5N2)	KJ018197	KJ018198	KJ018199	KJ018201	KJ018203	KJ018204
A/chicken/New Jersey/577-6/2008(H5N2)	KJ018189	KJ018190	KJ018191	KJ018193	KJ018195	KJ018196
A/chicken/New York/10196-4/2005(H7N2)	CY034269	CY034268	CY034267	CY034265	CY034263	CY034266
A/chicken/New York/10508/2005(H7N2)	CY034845	CY034844	CY034843	CY034841	CY034839	CY034842
A/chicken/New York/16326-4/2005(H7N2)	CY029840	CY029839	CY029838	CY029836	CY029834	CY029837
A/chicken/New York/16326-5/2005(H7N2)	CY022820	CY022819	CY022818	CY022816	CY022814	CY022817
A/chicken/New York/19499/2005(H7N2)	CY034253	CY034252	CY034251	CY034249	CY034247	CY034250
A/chicken/New York/19499-1/2006(H7N2)	CY036017	CY036016	CY036015	CY036013	CY036011	CY036014
A/chicken/New York/21211-2/2005(H7N2)	CY022836	CY022835	CY022834	CY022832	CY022830	CY022833
A/chicken/New York/22067-7/2005(H7N2)	CY031642	CY031641	CY031640	CY031638	CY031636	CY031639
A/chicken/New York/23165-6/2005(H7N2)	CY031083	CY031082	CY031081	CY031079	CY031077	CY031080
A/chicken/New York/29047-4/2006(H7N2)	CY028635	CY028634	CY028633	CY028631	CY028629	CY028632
A/chicken/New York/30732-6/2005(H7N2)	CY031123	CY031122	CY031121	CY031119	CY031117	CY031120
A/chicken/New York/31621-9/2005(H7N2)	CY031754	CY031753	CY031752	CY031750	CY031748	CY031751
A/chicken/New York/3181-5/2006(H7N2)	CY028547	CY028546	CY028545	CY028543	CY028541	CY028544
A/chicken/New York/42217/2005(H7N2)	CY031778	CY031777	CY031776	CY031774	CY031772	CY031775
A/chicken/New York/439235/2006(H5N2)	GQ117136	GQ117135	GQ117134	GQ117132	GQ117130	GQ117133
A/chicken/New York/63806-7/2005(H7N2)	CY095107	CY095106	CY095105	CY095103	CY095101	CY095104
A/chicken/New York/63806-8/2005(H7N2)	CY033312	CY033311	CY033310	CY033308	CY033306	CY033309
A/chicken/New York/79672/2005(H7N2)	CY095115	CY095114	CY095113	CY095111	CY095109	CY095112

A/chicken/New York/8391-2/2006(H7N2)	CY028587	CY028586	CY028585		CY028583		CY028581	CY028584
A/chicken/New York/87493-1/2005(H7N2)	CY095123	CY095122	CY095121		CY095119		CY095117	CY095120
A/chicken/New York/87493-2/2005(H7N2)	CY037102	CY037101	CY037100		CY037098		CY037096	CY037099
A/chicken/New York/88291-11/2005(H7N2)	CY034373	CY034372	CY034371		CY034369		CY034367	CY034370
A/chicken/New York/88291-8/2005(H7N2)	CY034805	CY034804	CY034803		CY034801		CY034799	CY034802
A/chicken/NJ/294508-12/2004(H7N2)	EU743260	EU743259	EU743258		EU743256		EU743254	EU743257
A/chicken/NJ/30749-3/2000(H7N2)	EU084903	EU084904	EU084905		EU084906		AY241606	AY241645
A/chicken/NY/119256-7/2001(H7N2)	EU084909	EU084910	EU084911		EU084912		AY241599	AY241638
A/chicken/PA/149092-1/2002(H7N2)	EU742973	EU742972	EU742971		EU742970		AY241609	AY241647
A/chicken/SK/HR-00011/2007(H7N3)	EU500857	EU500858	EU500859		EU500861		EU500863	EU500864
A/chicken/TX/298313/04(H5N2)	AY849787	AY849785			AY849790		AY849792	AY849788
A/chicken/Veracruz/1433-1/2006(H5N2)	KM455878	KM455879	KM455880		KM455882		KM455884	KM455885
A/chukar/New York/21211-7/2005(H7N2)	CY024849	CY024848	CY024847		CY024845		CY024843	CY024846
A/cinnamon teal/Mexico/2817/2006(H7N3)	KC669385	KC669384	KC669387		KC669390		KC669383	KC669388
A/common goldeneye/Iowa/3192/2009(H11N9)	CY097073	CY097072	CY097071		CY097069		CY097067	CY097070
A/common goldeneye/Wisconsin/10OS4202/2010(H7N6)	CY133136	CY133135	CY133134		CY133132		CY133130	CY133133
A/common murre/Oregon/20361-001/2007(H10N7)	CY076108	CY076107	CY076106		CY076104		CY076102	CY076105
A/domestic duck/West Virginia/A00140915/2008(H7N3)	KU289998	KU289997	KU289996		KU289994		KU289992	KU289995
A/duck/Minnesota/462960-2/2006(H5N2)	GQ923180	GQ923179	GQ923178		GQ923176		GQ923174	GQ923177
A/duck/New Jersey/10-001022-21/2010(H7N3)	KY551140	KY551141	KY551142		KY551144		KY551146	KY551147
A/duck/New York/21211-6/2005(H7N2)	CY022852	CY022851	CY022850		CY022848		CY022846	CY022849
A/duck/New York/445743/2006(H5N2)	GQ117144	GQ117143	GQ117142		GQ117140		GQ117138	GQ117141
A/duck/New York/469961/2006(H5N2)	GQ117200	GQ117199	GQ117198		GQ117196		GQ117194	GQ117197
A/duck/New York/489761/2007(H5N2)	GQ117248	GQ117247	GQ117246		GQ117244		GQ117242	GQ117245
A/duck/New York/492652/2007(H5N2)	GQ257440	GQ257439	GQ257438		GQ257436		GQ257434	GQ257437
A/duck/New York/494165/2007(H5N2)	GQ117272	GQ117271	GQ117270		GQ117268		GQ117266	GQ117269
A/duck/New York/504371/2007(H5N2)	GQ923564	GQ923563	GQ923562		GQ923560		GQ923558	GQ923561
A/duck/New York/504372/2007(H5N2)	GQ923540	GQ923539	GQ923538		GQ923536		GQ923534	GQ923537
A/duck/New York/53294-6/2005(H2N3)	CY095187	CY095186	CY095185		CY095183		CY095181	CY095184
A/duck/New York/88291-13/2005(H7N2)	CY034381	CY034380	CY034379		CY034377		CY034375	CY034378
A/duck/New York/98616-5/2005(H7N2)	CY034821	CY034820	CY034819		CY034817		CY034815	CY034818
A/Duck/NJ/117228-7/2001(H5N2)	EU084940	EU084941	EU084942		EU084943		AY300965	AY300989
A/Duck/NY/185502/2002(H5N2)	EU084949	EU084950	EU084951		EU084952		AY300968	AY300992
A/duck/NY/44018-1/00(H5N2)							AY300959	
A/Duck/NY/44018-1/2000(H5N2)	EU084936	EU084937	EU084938		EU084939			AY300983
A/duck/PA/143585/2001(H7N3)	EU743014	EU743013	EU743012		EU743010		AY241616	EU743011
A/duck/Pennsylvania/02099/2012(H11N9)	KR870234	KR870235	KR870236		KR870238		KR870240	KR870241
A/duck/Pennsylvania/446080-7/2006(H5N2)	GQ923436	GQ923435	GQ923434		GQ923432		GQ923430	GQ923433
A/duck/Pennsylvania/4844-6/2013(H1N8)	KF382700	KF382701	KF382702		KF382704		KF382706	KF382707
A/environment/Maryland/05OS1161/2005(H3N8)	CY189954	CY189953	CY189952	CY189947	CY189950	CY189949	CY189948	CY189951
A/environment/Maryland/05OS1166/2005(H3N8)	CY189962	CY189961	CY189960	CY189955	CY189958	CY189957	CY189956	CY189959
A/environment/Maryland/06OS1166/2006(H5N1)	CY190106	CY190105	CY190104	CY190099	CY190102	CY190101	CY190100	CY190103
A/environment/Maryland/06OS1175/2006(H5N1)	CY190138	CY190137	CY190136	CY190131	CY190134	CY190133	CY190132	CY190135
A/environment/Maryland/06OS1627/2006(H7N3)	CY190218	CY190217	CY190216	CY190211	CY190214	CY190213	CY190212	CY190215

A/environment/Maryland/06OS1628/2006(H7N3)	CY190226	CY190225	CY190224	CY190219	CY190222	CY190221	CY190220	CY190223
A/environment/Maryland/07OS1592/2007(H3N8)	CY190370	CY190369	CY190368	CY190363	CY190366	CY190365	CY190364	CY190367
A/environment/Maryland/07OS1597/2007(H3N8)	CY190378	CY190377	CY190376	CY190371	CY190374	CY190373	CY190372	CY190375
A/environment/Maryland/08OS1550/2008(H11N9)	CY190674	CY190673	CY190672	CY190667	CY190670	CY190669	CY190668	CY190671
A/environment/Maryland/08OS1579/2008(H11N9)	CY190682	CY190681	CY190680	CY190675	CY190678	CY190677	CY190676	CY190679
A/environment/Maryland/09OS1030/2009(H6N8)	CY190754	CY190753	CY190752	CY190747	CY190750	CY190749	CY190748	CY190751
A/environment/Maryland/09OS1031/2009(H6N8)	CY190762	CY190761	CY190760	CY190755	CY190758	CY190757	CY190756	CY190759
A/environment/Maryland/09OS1290/2009(H4N6)	CY190802	CY190801	CY190800	CY190795	CY190798	CY190797	CY190796	CY190799
A/environment/Maryland/09OS1296/2009(H12N5)	CY190842	CY190841	CY190840	CY190835	CY190838	CY190837	CY190836	CY190839
A/environment/Maryland/09OS1459/2009(H3N8)	CY204962	CY204961	CY204960	CY204955	CY204958	CY204957	CY204956	CY204959
A/environment/Maryland/1101/2006(H4N6)	CY025108	CY025107	CY025106	CY025101	CY025104	CY025103	CY025102	CY025105
A/environment/Maryland/1106/2006(H4N6)	CY089506	CY089505	CY089504	CY089498	CY089501	CY089500	CY089499	CY089503
A/environment/Maryland/1158/2005(H3N8)	CY024809	CY024808	CY024807	CY024802	CY024805	CY024804	CY024803	CY024806
A/environment/Maryland/1158/2006(H5N1)	JF758704	JF758703	JF758707	JF758707	JF758706	JF758709	JF758708	JF758705
A/environment/Maryland/1165/2005(H3N8)	CY021316	CY021315	CY021314	CY021309	CY021312	CY021311	CY021310	CY021313
A/environment/Maryland/1183/2005(H3N8)	CY056283	CY056282	CY056281	CY056276	CY056279	CY056278	CY056277	CY056280
A/environment/Maryland/12OS1465/2012(H3N6)	CY191050	CY191049	CY191048	CY191043	CY191046	CY191045	CY191044	CY191047
A/environment/Maryland/12OS1472/2012(H3N6)	CY191082	CY191081	CY191080	CY191075	CY191078	CY191077	CY191076	CY191079
A/environment/Maryland/12OS1539/2012(H3N6)	CY205162	CY205161	CY205160	CY205155	CY205158	CY205157	CY205156	CY205159
A/environment/Maryland/12OS1558/2012(H3N6)	CY205170	CY205169	CY205168	CY205163	CY205166	CY205165	CY205164	CY205167
A/environment/Maryland/1630/2006(H7N3)	JF758771	JF758768	JF758769	JF758766	JF758765	JF758768	JF758767	JF758766
A/environment/Maryland/1637/2006(H7N3)	JF758774	JF758773	JF758778	JF758774	JF758780	JF758776	JF758775	JF758777
A/environment/Maryland/2103/2006(mixed)	JF758798	JF758791	JF758796	JF758789	JF758794	JF758791	JF758790	JF758792
A/environment/Maryland/2115/2006(H4N6)	CY053324	CY053323	CY053322	CY053317	CY053320	CY053319	CY053318	CY053321
A/environment/Maryland/763/2006(H11N9)	CY025100	CY025099	CY025098	CY025093	CY025096	CY025095	CY025094	CY025097
A/gadwall duck/Minnesota/Sg-00575/2008(H3N8)	CY140012	CY140011	CY042626		CY140008		CY140006	CY140009
A/gadwall/California/HKWF100/2007(H6N1)	CY032661	CY032662	CY032663		CY032665		CY032667	CY032668
A/gadwall/Illinois/10OS3342/2010(H6N1)	CY132988	CY132987	CY132986		CY132984		CY132982	CY132985
A/gadwall/Illinois/10OS3384/2010(H11N2)	CY133012	CY133011	CY133010		CY133008		CY133006	CY133009
A/gadwall/Illinois/3860/2009(H6N1)	CY097525	CY097524	CY097523		CY097521		CY097519	CY097522
A/gadwall/Iowa/10OS2753/2010(H3N8)	CY133479	CY133478	CY133477		CY133475		CY133473	CY133476
A/gadwall/Mississippi/11OS6085/2011(H6N1)	CY166823	CY166822	CY166821		CY166819		CY166817	CY166820
A/gadwall/Missouri/10MO0280/2010(H5N2)	CY133816	CY133815	CY133814		CY133812		CY133810	CY133813
A/gadwall/Missouri/10MO095/2010(H6N1)	CY133800	CY133799	CY133798		CY133796		CY133794	CY133797
A/gadwall/Missouri/10OS4731/2010(H7N3)	CY133415	CY133414	CY133413		CY133411		CY133409	CY133412
A/gadwall/Ohio/12OS2222/2012(H3N8)	CY187020	CY187019	CY187018		CY187016		CY187014	CY187017
A/gadwall/Ohio/12OS5632/2012(H5N2)	CY186988	CY186987	CY186986		CY186984		CY186982	CY186985
A/gadwall/Wisconsin/11OS3415/2011(H4N6)	CY166612	CY166611	CY166610		CY166608		CY166606	CY166609
A/greater white-fronted goose/California/44358-095/2007(H6N1)	CY076668	CY076667	CY076666		CY076664		CY076662	CY076665
A/greater white-fronted goose/California/44358-112/2007(H6N1)	CY076676	CY076675	CY076674		CY076672		CY076670	CY076673
A/greater white-fronted goose/California/HKWF446C/2007(H10N7)	CY094508	CY094507	CY094506		CY094504		CY094502	CY094505
A/green winged teal/Delaware/458672-5/2006(H5N2)	GQ923508	GQ923507	GQ923506		GQ923504		GQ923502	GQ923505
A/green winged teal/Iowa/463990-3/2006(H5N2)	GQ923188	GQ923187	GQ923186		GQ923184		GQ923182	GQ923185
A/green winged teal/Louisiana/Sg-00090/2007(H1N1)	KF424087	KF424088	KF424089		KF424091		KF424093	KF424094

A/green winged teal/Ohio/464069/2006(H5N2)	GQ923212	GQ923211	GQ923210	GQ923208	GQ923206	GQ923209
A/green winged teal/Ohio/468160/2006(H5N2)	GQ923292	GQ923291	GQ923290	GQ923288	GQ923286	GQ923289
A/green-winged teal/Alberta/11383/2005(H4N6)	CY047543	CY047542	CY047541	CY047539	CY047537	CY047540
A/green-winged teal/California/8326/2008(H1N2)	CY094452	CY094451	CY094450	CY094448	CY094446	CY094449
A/green-winged teal/Louisiana/Sg-00092/2007(H3N8)	CY078272	CY078271	CY078270	CY078268	CY078266	CY078269
A/green-winged teal/Minnesota/Sg-01073/2008(H6N2)	CY140638	CY140637	CY140636	CY140634	CY140632	CY140635
A/green-winged teal/Missouri/13OS6332/2013(H10N3)	KX013048	KX013041	KX013039	KX013050	KX013070	KX544825
A/green-winged teal/Nova Scotia/00354/2010(H3N8)	CY138800	CY138799	CY138798	CY138796	CY138794	CY138797
A/green-winged teal/Ohio/1292/2005(H4N6)	CY021356	CY021355	CY021354	CY021352	CY021350	CY021353
A/green-winged teal/Ohio/1844/2005(H11N9)	CY021180	CY021179	CY021178	CY021176	CY021174	CY021177
A/green-winged teal/Texas/AI11-3608/2011(H4N6)	CY206002	CY206001	CY206000	CY205998	CY205996	CY205999
A/guinea fowl/Massachusetts/14075-3/2013(H12N5)	KM244057	KM244058	KM244059	KM244061	KM244063	KM244064
A/Guinea fowl/New York/101276-1/2005(H7N2)	CY034837	CY034836	CY034835	CY034833	CY034831	CY034834
A/guinea fowl/New York/143646-3/2005(H7N2)	CY028514	CY028513	CY028512	CY028510	CY028508	CY028511
A/guinea fowl/New York/22071/2005(H7N2)	CY031658	CY031657	CY031656	CY031654	CY031652	CY031655
A/guinea fowl/New York/23164-3-05/2005(H7N2)	CY031682	CY031681	CY031680	CY031678	CY031676	CY031679
A/guinea fowl/New York/23165-12/2005(H7N2)	CY031730	CY031729	CY031728	CY031726	CY031724	CY031727
A/guinea fowl/New York/32084/2006(H7N2)	CY034245	CY034244	CY034243	CY034241	CY034239	CY034242
A/guinea fowl/New York/8391-1/2006(H7N2)	CY028579	CY028578	CY028577	CY028575	CY028573	CY028576
A/Guinea fowl/New York/88291-9/2005(H7N2)	CY035929	CY035928	CY035927	CY035925	CY035923	CY035926
A/Guinea fowl/New York/98616-3/2005(H7N2)	CY037070	CY037069	CY037068	CY037066	CY037064	CY037067
A/guinea fowl/NJ/119063-8/2001(H7N2)	EU742919	EU742918	EU742917	EU742915	EU742913	EU742916
A/Guinea fowl/Oregon/459674-5/2006(H5N2)	GQ923140	GQ923139	GQ923138	GQ923136	GQ923134	GQ923137
A/herring gull/New Jersey/AI09-335/2009(H11N1)	CY145962	CY145961	CY145960	CY145958	CY145956	CY145959
A/laughing gull/Delaware Bay/42/2006(H7N3)	CY102974	CY102973	CY185866	CY185864	CY185862	CY102971
A/laughing gull/Delaware/42/06(H7N3)	EU030982	EU030981	EU030983	EU030985	EU030987	EU030988
A/laughing gull/New York/AI00-2455/2000(H7N3)	CY144299	CY144298	CY144297	CY144295	CY144293	CY144296
A/laughing gull/New York/AI00-470/2000(H6N1)	CY144169	CY144168	CY144167	CY144165	CY144163	CY144166
A/least sandpiper/South Central Alaska/2/2007(H4N8)	CY035780	CY035781	CY035782	CY035784	CY035786	CY035787
A/least sandpiper/South Central Alaska/7KW0434/2007(H4N8)	CY077332	CY077331	CY077330	CY077328	CY077326	CY077329
A/lesser scaup/Illinois/4115/2009(H10N7)	CY097533	CY097532	CY097531	CY097529	CY097527	CY097530
A/lesser scaup/Louisiana/AI09-5204/2009(H6N8)	CY140992	CY140991	CY140990	CY140988	CY140986	CY140989
A/lesser scaup/Wisconsin/3964/2009(H10N3)	CY097300	CY097299	CY097298	CY097296	CY097294	CY097297
A/longtail duck/Maryland/291/2005(H3N8)	CY017780	CY017779	CY017778	CY017776	CY017774	CY017777
A/longtail duck/Maryland/295/2005(H10N8)	CY020908	CY020907	CY020906	CY020904	CY020902	CY020905
A/long-tailed duck/Maine/295/2011(H3N8)	CY149595	CY149594	CY149593	CY149591	CY149589	CY149592
A/long-tailed duck/Wisconsin/10OS3915/2010(H3N6)	CY133552	CY133551	CY133550	CY133548	CY133546	CY133549
A/long-tailed duck/Wisconsin/10OS3919/2010(H10N6)	CY133568	CY133567	CY133566	CY133564	CY133562	CY133565
A/mallard/Alberta/160/2001(H3N8)	CY102695	CY102694	CY102693	CY102691	CY102689	CY102692
A/mallard/Alberta/234/2007(H12N5)	CY103339	CY077196	CY077195	CY077193	CY077191	CY077194
A/mallard/Arkansas/473507-9/2006(H5N2)	GQ923348	GQ923347	GQ923346	GQ923344	GQ923342	GQ923345
A/mallard/British Columbia/07826/2005(H5N2)	CY047503	CY047502	CY047501	CY047499	CY047497	CY047500
A/mallard/California/1289/2010(H4N6)	CY094788	CY094787	CY094786	CY094784	CY094782	CY094785
A/mallard/California/1390/2010(H7N5)	CY120562	CY120561	CY120560	CY120558	CY120556	CY120559

A/mallard/California/3188/2010(H6N8)	CY120650	CY120649	CY120648		CY120646		CY120644	CY120647
A/mallard/California/6524/2008(H12N5)	CY094124	CY094123	CY094122		CY094120		CY094118	CY094121
A/mallard/California/9573/2008(H4N6)	CY094236	CY094235	CY094234		CY094232		CY094230	CY094233
A/mallard/Idaho/ND0002644/2013(H3N8)	KM373967	KM373968	KM373969		KM373971		KM373973	KM373974
A/mallard/Illinois/10OS3786/2010(H10N7)	CY132656	CY132655	CY132654		CY132652		CY132650	CY132653
A/mallard/Illinois/10OS4078/2010(H10N7)	CY133496	CY133495	CY133494		CY133492		CY133490	CY133493
A/mallard/Illinois/10OS4111/2010(H10N7)	CY133504	CY133503	CY133502		CY133500		CY133498	CY133501
A/mallard/Illinois/11OS4420/2011(H2N3)	CY167010	CY167009	CY167008		CY167006		CY167004	CY167007
A/mallard/Illinois/11OS5564/2011(H1N8)	CY166289	CY166288	CY166287		CY166285		CY166283	CY166286
A/mallard/Illinois/11OS5576/2011(H5N3)	CY166305	CY166304	CY166303		CY166301		CY166299	CY166302
A/mallard/Illinois/4124/2009(H4N8)	CY097549	CY097548	CY097547		CY097545		CY097543	CY097546
A/mallard/Iowa/10OS2692/2010(H4N2)	CY133447	CY133446	CY133445		CY133443		CY133441	CY133444
A/mallard/Iowa/A00558620/2008(H7N3)	KU290046	KU290045	KU290044		KU290042		KU290040	KU290043
A/mallard/Maryland/05OS1142/2005(mixed)	CY202979	CY202978	CY202977	CY202971	CY202975	CY202973	CY202972	CY202976
A/mallard/Maryland/05OS1244/2005(H1N1)	CY203612	CY203611	CY203610	CY203605	CY203608	CY203607	CY203606	CY203609
A/mallard/Maryland/05OS1403/2005(H3N8)	CY202987	CY202986	CY202985	CY202980	CY202983	CY202982	CY202981	CY202984
A/mallard/Maryland/05OS1508/2005(H3N8)	CY203620	CY203619	CY203618	CY203613	CY203616	CY203615	CY203614	CY203617
A/mallard/Maryland/05OS606/2005(H6N8)	CY203548	CY203547	CY203546	CY203541	CY203544	CY203543	CY203542	CY203545
A/mallard/Maryland/05OS622/2005(H3N8)	CY203556	CY203555	CY203554	CY203549	CY203552	CY203551	CY203550	CY203553
A/mallard/Maryland/06OS101/2006(H7N4)	CY203628	CY203627	CY203626	CY203621	CY203624	CY203623	CY203622	CY203625
A/mallard/Maryland/06OS120/2006(H6N8)	CY203636	CY203635	CY203634	CY203629	CY203632	CY203631	CY203630	CY203633
A/mallard/Maryland/06OS1207/2006(H3N6)	CY203708	CY203707	CY203706	CY203701	CY203704	CY203703	CY203702	CY203705
A/mallard/Maryland/06OS1236/2006(H11N9)	CY203716	CY203715	CY203714	CY203709	CY203712	CY203711	CY203710	CY203713
A/mallard/Maryland/06OS194/2006(H10N4)	CY190306	CY190305	CY190304	CY190299	CY190302	CY190301	CY190300	CY190303
A/mallard/Maryland/06OS196/2006(H6N2)	CY190314	CY190313	CY190312	CY190307	CY190310	CY190309	CY190308	CY190311
A/mallard/Maryland/06OS2334/2006(H12N5)	CY190330	CY190329	CY190328	CY190323	CY190326	CY190325	CY190324	CY190327
A/mallard/Maryland/06OS2409/2006(H10N4)	CY190338	CY190337	CY190336	CY190331	CY190334	CY190333	CY190332	CY190335
A/mallard/Maryland/06OS2460/2006(H2N2)	CY190346	CY190345	CY190344	CY190339	CY190342	CY190341	CY190340	CY190343
A/mallard/Maryland/06OS444/2006(H12N8)	CY203644	CY203643	CY203642	CY203637	CY203640	CY203639	CY203638	CY203641
A/mallard/Maryland/06OS456/2006(mixed)	CY202619	CY202618	CY202617	CY202611	CY202615	CY202613	CY202612	CY202616
A/mallard/Maryland/06OS487/2006(H6N5)	CY203676	CY203675	CY203674	CY203669	CY203672	CY203671	CY203670	CY203673
A/mallard/Maryland/06OS488/2006(H6N5)	CY203684	CY203683	CY203682	CY203677	CY203680	CY203679	CY203678	CY203681
A/mallard/Maryland/07OS1217/2007(H5N9)	CY203756	CY203755	CY203754	CY203749	CY203752	CY203751	CY203750	CY203753
A/mallard/Maryland/07OS1309/2007(H4N2)	CY203764	CY203763	CY203762	CY203757	CY203760	CY203759	CY203758	CY203761
A/mallard/Maryland/07OS1315/2007(H4N2)	CY203772	CY203771	CY203770	CY203765	CY203768	CY203767	CY203766	CY203769
A/mallard/Maryland/07OS1544/2007(H12N9)	CY202995	CY202994	CY202993	CY202988	CY202991	CY202990	CY202989	CY202992
A/mallard/Maryland/07OS1563/2007(H11N9)	CY203003	CY203002	CY203001	CY202996	CY202999	CY202998	CY202997	CY203000
A/mallard/Maryland/07OS1669/2007(H4N6)	CY202627	CY202626	CY202625	CY202620	CY202623	CY202622	CY202621	CY202624
A/mallard/Maryland/07OS55/2007(H10N7)	CY190602	CY190601	CY190600	CY190595	CY190598	CY190597	CY190596	CY190599
A/mallard/Maryland/07OS56/2007(H10N7)	CY190610	CY190609	CY190608	CY190603	CY190606	CY190605	CY190604	CY190607
A/mallard/Maryland/07OS742/2007(H1N8)	CY202600	CY202599	CY202598	CY202593	CY202596	CY202595	CY202594	CY202597
A/mallard/Maryland/07OS832/2007(H9N1)	CY203724	CY203723	CY203722	CY203717	CY203720	CY203719	CY203718	CY203721
A/mallard/Maryland/07OS864/2007(H5N8)	CY203732	CY203731	CY203730	CY203725	CY203728	CY203727	CY203726	CY203729
A/mallard/Maryland/07OS871/2007(H10N9)	CY203740	CY203739	CY203738	CY203733	CY203736	CY203735	CY203734	CY203737

A/mallard/Maryland/08OS1331/2008(H1N1)	CY203011	CY203010	CY203009	CY203004	CY203007	CY203006	CY203005	CY203008
A/mallard/Maryland/08OS1332/2008(H1N1)	CY203019	CY203018	CY203017	CY203012	CY203015	CY203014	CY203013	CY203016
A/mallard/Maryland/08OS1414/2008(H4N6)	CY202674	CY202673	CY202672	CY202667	CY202670	CY202669	CY202668	CY202671
A/mallard/Maryland/08OS1416/2008(H1N1)	CY203852	CY203851	CY203850	CY203845	CY203848	CY203847	CY203846	CY203849
A/mallard/Maryland/08OS3074/2008(H10N7)	CY190698	CY190697	CY190696	CY190691	CY190694	CY190693	CY190692	CY190695
A/mallard/Maryland/08OS3075/2008(H10N7)	CY190706	CY190705	CY190704	CY190699	CY190702	CY190701	CY190700	CY190703
A/mallard/Maryland/09OS1002/2009(H6N8)	CY202825	CY202824	CY202823	CY202818	CY202821	CY202820	CY202819	CY202822
A/mallard/Maryland/09OS1025/2009(H6N8)	CY202833	CY202832	CY202831	CY202826	CY202829	CY202828	CY202827	CY202830
A/mallard/Maryland/09OS1083/2009(H12N5)	CY203868	CY203867	CY203866	CY203861	CY203864	CY203863	CY203862	CY203865
A/mallard/Maryland/09OS1090/2009(H12N5)	CY203876	CY203875	CY203874	CY203869	CY203872	CY203871	CY203870	CY203873
A/mallard/Maryland/09OS1226/2009(H6N8)	CY190794	CY190793	CY190792	CY190787	CY190790	CY190789	CY190788	CY190791
A/mallard/Maryland/09OS1361/2009(H4N6)	CY203203	CY203202	CY203201	CY203196	CY203199	CY203198	CY203197	CY203200
A/mallard/Maryland/09OS1362/2009(H4N6)	CY203211	CY203210	CY203209	CY203204	CY203207	CY203206	CY203205	CY203208
A/mallard/Maryland/09OS1400/2009(H3N8)	CY204946	CY204945	CY204944	CY204939	CY204942	CY204941	CY204940	CY204943
A/mallard/Maryland/09OS2002/2009(mixed)	CY202742	CY202741	CY202740	CY202734	CY202738	CY202736	CY202735	CY202739
A/mallard/Maryland/09OS2006/2009(H1N1)	CY204970	CY204969	CY204968	CY204963	CY204966	CY204965	CY204964	CY204967
A/mallard/Maryland/09OS2221/2009(H1N1)	CY190962	CY190961	CY190960	CY190955	CY190958	CY190957	CY190956	CY190959
A/mallard/Maryland/09OS2237/2009(H10N6)	CY190970	CY190969	CY190968	CY190963	CY190966	CY190965	CY190964	CY190967
A/mallard/Maryland/09OS254/2009(H1N1)	CY190978	CY190977	CY190976	CY190971	CY190974	CY190973	CY190972	CY190975
A/mallard/Maryland/10OS064/2010(H2N3)	CY204994	CY204993	CY204992	CY204987	CY204990	CY204989	CY204988	CY204991
A/mallard/Maryland/10OS0879/2010(mixed)	CY202768	CY202767	CY202766	CY202760	CY202764	CY202762	CY202761	CY202765
A/mallard/Maryland/10OS0956/2010(H4N6)	CY205378	CY205377	CY205376	CY205371	CY205374	CY205373	CY205372	CY205375
A/mallard/Maryland/10OS1040/2010(H4N6)	CY204986	CY204985	CY204984	CY204979	CY204982	CY204981	CY204980	CY204983
A/mallard/Maryland/10OS1087/2010(H3N2)	CY203268	CY203267	CY203266	CY203261	CY203264	CY203263	CY203262	CY203265
A/mallard/Maryland/10OS1094/2010(H3N2)	CY203292	CY203291	CY203290	CY203285	CY203288	CY203287	CY203286	CY203289
A/mallard/Maryland/10OS118/2010(H2N3)	CY202865	CY202864	CY202863	CY202858	CY202861	CY202860	CY202859	CY202862
A/mallard/Maryland/10OS121/2010(H2N3)	CY202873	CY202872	CY202871	CY202866	CY202869	CY202868	CY202867	CY202870
A/mallard/Maryland/10OS1907/2010(H3N6)	CY203316	CY203315	CY203314	CY203309	CY203312	CY203311	CY203310	CY203313
A/mallard/Maryland/10OS1934/2010(H3N2)	CY203332	CY203331	CY203330	CY203325	CY203328	CY203327	CY203326	CY203329
A/mallard/Maryland/10OS2170/2010(H6N2)	CY190986	CY190985	CY190984	CY190979	CY190982	CY190981	CY190980	CY190983
A/mallard/Maryland/10OS2271/2010(H11N9)	CY202897	CY202896	CY202895	CY202890	CY202893	CY202892	CY202891	CY202894
A/mallard/Maryland/10OS2293/2010(H11N9)	CY202905	CY202904	CY202903	CY202898	CY202901	CY202900	CY202899	CY202902
A/mallard/Maryland/10OS566/2010(H10N7)	CY205002	CY205001	CY205000	CY204995	CY204998	CY204997	CY204996	CY204999
A/mallard/Maryland/1111/2005(H11N9)	CY081307	CY081306	CY081305	CY081300	CY081303	CY081302	CY081301	CY081304
A/mallard/Maryland/1124/2005(H11N9)	CY021476	CY021475	CY021474	CY021469	CY021472	CY021471	CY021470	CY021473
A/mallard/Maryland/1127/2005(H3N8)	CY081315	CY081314	CY081313	CY081308	CY081311	CY081310	CY081309	CY081312
A/mallard/Maryland/1143/2005(mixed)	CY081403	CY081402	CY081401	CY081396	CY081399	CY081398	CY081397	CY081400
A/mallard/Maryland/11OS1553/2011(H5N2)	CY205042	CY205041	CY205040	CY205035	CY205038	CY205037	CY205036	CY205039
A/mallard/Maryland/11OS1704/2011(H5N2)	CY203356	CY203355	CY203354	CY203349	CY203352	CY203351	CY203350	CY203353
A/mallard/Maryland/11OS1714/2011(H5N2)	CY203364	CY203363	CY203362	CY203357	CY203360	CY203359	CY203358	CY203361
A/mallard/Maryland/11OS1804/2011(H2N3)	CY205050	CY205049	CY205048	CY205043	CY205046	CY205045	CY205044	CY205047
A/mallard/Maryland/11OS1809/2011(H2N3)	CY205058	CY205057	CY205056	CY205051	CY205054	CY205053	CY205052	CY205055
A/mallard/Maryland/11OS3603/2011(H5N2)	CY190994	CY190993	CY190992	CY190987	CY190990	CY190989	CY190988	CY190991
A/mallard/Maryland/11OS3701/2011(H6N1)	CY191002	CY191001	CY191000	CY190995	CY190998	CY190997	CY190996	CY190999

A/mallard/Maryland/11OS3829/2012(H2N2)	CY191018	CY191017	CY191016	CY191011	CY191014	CY191013	CY191012	CY191015
A/mallard/Maryland/11OS3833/2012(H2N1)	CY191026	CY191025	CY191024	CY191019	CY191022	CY191021	CY191020	CY191023
A/mallard/Maryland/11OS3856/2011(H2N2)	CY205026	CY205025	CY205024	CY205019	CY205022	CY205021	CY205020	CY205023
A/mallard/Maryland/11OS3951/2011(H2N2)	CY205034	CY205033	CY205032	CY205027	CY205030	CY205029	CY205028	CY205031
A/mallard/Maryland/11OS906/2011(H12N5)	CY205010	CY205009	CY205008	CY205003	CY205006	CY205005	CY205004	CY205007
A/mallard/Maryland/11OS925/2011(H12N5)	CY205018	CY205017	CY205016	CY205011	CY205014	CY205013	CY205012	CY205015
A/mallard/Maryland/11OS951/2011(H4N6)	CY205090	CY205089	CY205088	CY205083	CY205086	CY205085	CY205084	CY205087
A/mallard/Maryland/11OS952/2011(H4N6)	CY205098	CY205097	CY205096	CY205091	CY205094	CY205093	CY205092	CY205095
A/mallard/Maryland/1211/2006(H11N9)	CY095615	CY095614	CY095613	CY095608	CY095611	CY095610	CY095609	CY095612
A/mallard/Maryland/1241/2005(H4N6)	CY021340	CY021339	CY021338	CY021333	CY021336	CY021335	CY021334	CY021337
A/mallard/Maryland/12OS1511/2012(H3N6)	CY205114	CY205113	CY205112	CY205107	CY205110	CY205109	CY205108	CY205111
A/mallard/Maryland/12OS1518/2012(H3N6)	CY205122	CY205121	CY205120	CY205115	CY205118	CY205117	CY205116	CY205119
A/mallard/Maryland/12OS3628/2013(H7N3)	CY205178	CY205177	CY205176	CY205171	CY205174	CY205173	CY205172	CY205175
A/mallard/Maryland/12OS3629/2013(H7N3)	CY205186	CY205185	CY205184	CY205179	CY205182	CY205181	CY205180	CY205183
A/mallard/Maryland/12OS3662/2013(H11N9)	CY191258	CY191257	CY191256	CY191251	CY191254	CY191253	CY191252	CY191255
A/mallard/Maryland/12OS565/2012(H2N2)	CY191266	CY191265	CY191264	CY191259	CY191262	CY191261	CY191260	CY191263
A/mallard/Maryland/12OS606/2012(H1N1)	CY202921	CY202920	CY202919	CY202914	CY202917	CY202916	CY202915	CY202918
A/mallard/Maryland/12OS608/2012(H1N1)	CY202929	CY202928	CY202927	CY202922	CY202925	CY202924	CY202923	CY202926
A/mallard/Maryland/13OS0611/2013(H4N8)	CY205202	CY205201	CY205200	CY205195	CY205198	CY205197	CY205196	CY205199
A/mallard/Maryland/13OS0755/2013(H9N2)	CY205210	CY205209	CY205208	CY205203	CY205206	CY205205	CY205204	CY205207
A/mallard/Maryland/13OS0772/2013(H10N7)	CY205218	CY205217	CY205216	CY205211	CY205214	CY205213	CY205212	CY205215
A/mallard/Maryland/13OS0875/2013(H2N3)	CY205234	CY205233	CY205232	CY205227	CY205230	CY205229	CY205228	CY205231
A/mallard/Maryland/13OS0877/2013(H2N3)	CY205242	CY205241	CY205240	CY205235	CY205238	CY205237	CY205236	CY205239
A/mallard/Maryland/13OS0878/2013(H2N3)	CY205250	CY205249	CY205248	CY205243	CY205246	CY205245	CY205244	CY205247
A/mallard/Maryland/13OS2946/2013(H5N2)	CY191274	CY191273	CY191272	CY191267	CY191270	CY191269	CY191268	CY191271
A/mallard/Maryland/1983/2005(H6N8)	CY024801	CY024800	CY024799	CY024794	CY024797	CY024796	CY024795	CY024798
A/mallard/Maryland/2022/2005(H6N8)	CY021484	CY021483	CY021482	CY021477	CY021480	CY021479	CY021478	CY021481
A/mallard/Maryland/296/2003(H2N3)	FJ686801	FJ686800	FJ686799	FJ686794	FJ686797	FJ686796	FJ686795	FJ686798
A/mallard/Maryland/301/2003(H2N3)	FJ686705	FJ686704	FJ686703	FJ686698	FJ686701	FJ686700	FJ686699	FJ686702
A/mallard/Maryland/350/2002(H1N1)	CY032212	CY032211	CY032210	CY032205	CY032208	CY032207	CY032206	CY032209
A/mallard/Maryland/369/2002(H1N1)		EU026064					EU026059	
A/mallard/Maryland/504/2006(mixed)	CY081411	CY081410	CY081409	CY081404	CY081407	CY081406	CY081405	CY081408
A/mallard/Maryland/538/2002(H11N9)	GQ257494	GQ257493	GQ257492	GQ257487	GQ257490	GQ257489	GQ257488	GQ257491
A/mallard/Maryland/615/2005(H3N2)								CY021457
A/mallard/Maryland/631/2005(H3N2)								CY021281
A/mallard/Maryland/715/2005(mixed)	CY081395	CY081394	CY081393	CY081388	CY081391	CY081390	CY081389	CY081392
A/mallard/Maryland/792/2007(H5N9)	CY089522	CY089521	CY089520	CY089515	CY089518	CY089517	CY089516	CY089519
A/mallard/Maryland/798/2007(mixed)	CY081419	CY081418	CY081417	CY081412	CY081415	CY081414	CY081413	CY081416
A/mallard/Maryland/800/2007(mixed)	CY081427	CY081426	CY081425	CY081420	CY081423	CY081422	CY081421	CY081424
A/mallard/Maryland/802/2007(H5N1)	JF758815	JF758809	JF758814	JF758811	JF758810	JF758813	JF758812	JF758808
A/mallard/Maryland/897/2004(H5N2)	FJ686745	FJ686744	FJ686743	FJ686738	FJ686741	FJ686740	FJ686739	FJ686742
A/mallard/Maryland/950/2004(H11N9)	GU053425	GU053424	GU053423	GU053418	GU053421	GU053420	GU053419	GU053422
A/mallard/Maryland/954/2004(H11N9)	GU053433	GU053432	GU053431	GU053426	GU053429	GU053428	GU053427	GU053430
A/mallard/MD/185/2003(H5N2)	EU980508	EU980507	EU980506	EU980501	EU980504	EU980503	EU980502	EU980505

A/mallard/MD/199/2003(H1N1)	EU980523	EU980522	EU980521	EU980516	EU980519	EU980518	EU980517	EU980520
A/mallard/MD/26/2003(H1N1)	FJ357119	FJ357118	FJ357117	FJ357112	FJ357115	FJ357114	FJ357113	FJ357116
A/mallard/MD/369/2002(H1N1)	EU026065		EU026063		EU026061			EU026062
A/mallard/MD/41/2003(H1N1)	FJ357127	FJ357126	FJ357125		FJ357123		FJ357121	
A/mallard/MD/42/2003(H1N1)	FJ357135	FJ357134	FJ357133		FJ357131		FJ357129	
A/mallard/Minnesota/464334/2006(H5N2)	GQ923220	GQ923219	GQ923218		GQ923216		GQ923214	GQ923217
A/mallard/Minnesota/AI08-3881/2008(H2N2)	CY141175	CY141174	CY141173		CY141171		CY141169	CY141172
A/mallard/Minnesota/AI09-3302/2009(H11N9)	CY140827	CY140826	CY140825		CY140823		CY140821	CY140824
A/mallard/Minnesota/AI09-3770/2009(H7N9)	CY186009	CY186008	CY186007		CY186005		CY186003	CY186006
A/mallard/Minnesota/Sg-00620/2008(H1N1)	CY140052	CY140051	CY140050		CY140048		CY140046	CY140049
A/mallard/Minnesota/Sg-00782/2008(H2N3)	CY140195	CY140194	CY140193		CY140191		CY140189	CY140192
A/mallard/Mississippi/354/2010(H3N8)	CY097709	CY097708	CY097707		CY097705		CY097703	CY097706
A/mallard/Missouri/10MO0253/2010(H1N2)	CY133808	CY133807	CY133806		CY133804		CY133802	CY133805
A/mallard/Missouri/11OS4987/2011(H5N2)	CY166930	CY166929	CY166928		CY166926		CY166924	CY166927
A/mallard/Missouri/129/2009(H6N2)	CY097597	CY097596	CY097595		CY097593		CY097591	CY097594
A/mallard/Missouri/220/2009(H7N3)	CY097629	CY097628	CY097627		CY097625		CY097623	CY097626
A/mallard/Missouri/MO32/2005(H11N9)	CY021692	CY021691	CY021690		CY021688		CY021686	CY021689
A/mallard/MN/42/2000(H11N9)	EU743464	EU743463	EU743462		EU743460		EU743458	EU743461
A/mallard/New Brunswick/00979/2010(H4N8)	CY139504	CY139503	CY139502		CY139500		CY139498	CY139501
A/mallard/New Jersey/Sg-00965/2008(H4N6)	CY145492	CY145491	CY145490		CY145488		CY145486	CY145489
A/mallard/New York/A00723392/2009(H7N3)	KU290099	KU290098	KU290097		KU290095		KU290093	KU290096
A/mallard/Ohio/11OS1966/2011(H10N3)	CY132164	CY132163	CY132162		CY132160		CY132158	CY132161
A/mallard/Ohio/11OS2048/2011(H1N1)	CY132196	CY132195	CY132194		CY132192		CY132190	CY132193
A/mallard/Ohio/11OS2149/2011(H2N3)	CY132284	CY132283	CY132282		CY132280		CY132278	CY132281
A/mallard/Ohio/11OS2216/2011(H5N1)	CY132452	CY132451	CY132450		CY132448		CY132446	CY132449
A/mallard/Ohio/12OS5547/2012(H4N6)	CY187052	CY187051	CY187050		CY187048		CY187046	CY187049
A/mallard/Ohio/13OS5188/2013(H10N8)	KX013077	KX013049	KX013062		KX013038		KX013081	KX013066
A/mallard/Ohio/1501/2006(H4N6)	CY089582	CY089581	CY089580		CY089577		CY089575	CY089578
A/mallard/Ohio/363/2003(H2N1)	FJ686729	FJ686728	FJ686727		FJ686725		FJ686723	FJ686726
A/mallard/Ohio/653/2002(H6N2)	CY020844	CY020843	CY020842		CY020840		CY020838	CY020841
A/mallard/Ohio/654/2002(H3N8)	CY020748	CY020747	CY020746		CY020744		CY020742	CY020745
A/mallard/Oregon/44221-105/2006(H3N6)	CY076180	CY076179	CY076178		CY076176		CY076174	CY076177
A/mallard/Quebec/11040/2006(H3N2)	CY047599	CY047598	CY047597		CY047595		CY047593	CY047596
A/mallard/South Dakota/A00649542/2008(H7N3)	KU290147	KU290146	KU290145		KU290143		KU290141	KU290144
A/mallard/Washington/44242-144/2006(H4N6)	CY076196	CY076195	CY076194		CY076192		CY076190	CY076193
A/mallard/Washington/44338-009/2007(H6N1)	CY076492	CY076491	CY076490		CY076488		CY076486	CY076489
A/mallard/Washington/44338-052/2007(H3N1)	CY076620	CY076619	CY076618		CY076616		CY076614	CY076617
A/mallard/Washington/456277-2/2006(H5N2)	GQ923492	GQ923491	GQ923490		GQ923488		GQ923486	GQ923489
A/mallard/Washington/456277-7/2006(H5N2)	GQ923484	GQ923483	GQ923482		GQ923480		GQ923478	GQ923481
A/mallard/Wisconsin/10OS1604/2010(H5N2)	CY132696	CY132695	CY132694		CY132692		CY132690	CY132693
A/mallard/Wisconsin/10OS4193/2010(H11N9)	CY133052	CY133051	CY133050		CY133048		CY133046	CY133049
A/mallard/Wisconsin/11OS3577/2011(H6N5)	CY166217	CY166216	CY166215		CY166213		CY166211	CY166214
A/mallard/Wisconsin/13OS2767/2013(H3N8)	KJ567958	KJ567959	KJ567960		KJ567962		KJ567964	KJ567965
A/mallard/Wisconsin/2530/2009(H4N2)	CY097453	CY097452	CY097451		CY097449		CY097447	CY097450

A/mallard/Wisconsin/2543/2009(H3N2)	CY096993	CY096992	CY096991	CY096989	CY096987	CY096990
A/Muscovy duck/New York/11646-4/2005(H7N2)	CY034853	CY034852	CY034851	CY034849	CY034847	CY034850
A/Muscovy duck/New York/62095-1/2006(H5N2)	CY036041	CY036040	CY036039	CY036037	CY036035	CY036038
A/Muscovy duck/New York/87493-3/2005(H7N2)	CY034797	CY034796	CY034795	CY034793	CY034791	CY034794
A/Muscovy duck/New York/97382-2/2005(H1N1)	CY095235	CY095234	CY095233	CY095231	CY095229	CY095232
A/mute swan/MI/451072-2/2006(H5N1)	GU186530	GU186529	GU186528	GU186526	GU186525	GU186527
A/mute swan/Rhode Island/A00325136/2008(H7N3)	KU290227	KU290226	KU290225	KU290223	KU290221	KU290224
A/northern pintail/California/183/2012(H1N1)	CY134374	CY134373	CY134372	CY134370	CY134368	CY134371
A/northern pintail/California/2548/2010(H11N2)	CY134398	CY134397	CY134396	CY134394	CY134392	CY134395
A/northern pintail/California/HKWF151/2007(H6N1)	CY035846	CY035847	CY035848	CY035850	CY035852	CY035853
A/northern pintail/Florida/480645-5/2007(H5N2)	GQ923412	GQ923411	GQ923410	GQ923408	GQ923406	GQ923409
A/northern pintail/Illinois/10OS3959/2010(H7N3)	CY132884	CY132883	CY132882	CY132880	CY132878	CY132881
A/northern pintail/Illinois/11OS4796/2011(H5N3)	CY167053	CY167052	CY167051	CY167049	CY167047	CY167050
A/northern pintail/Interior Alaska/1/2007(H1N1)	CY039744	CY039745	CY039746	CY039748	CY039750	CY039751
A/northern pintail/Interior Alaska/10BM02585R0/2010(H4N6)	CY130428	CY130427	CY130426	CY130424	CY130422	CY130425
A/northern pintail/Interior Alaska/10BM07040R0/2010(H4N6)	CY143467	CY143466	CY143465	CY143463	CY143461	CY143464
A/northern pintail/Interior Alaska/10BM08899R0/2010(H4N6)	CY143675	CY143674	CY143673	CY143671	CY143669	CY143672
A/northern pintail/Interior Alaska/10CH00003R0/2010(H4N6)	CY143755	CY143754	CY143753	CY143751	CY143749	CY143752
A/northern pintail/Interior Alaska/7MP0345BR2/2007(H3N8)	CY078906	CY078905	CY078904	CY078902	CY078900	CY078903
A/northern pintail/Interior Alaska/7MP1822/2007(H3N8)	CY079010	CY079009	CY079008	CY079006	CY079004	CY079007
A/northern pintail/Interior Alaska/8BM3658/2008(H4N6)	CY079146	CY079145	CY079144	CY079142	CY079140	CY079143
A/northern pintail/Interior Alaska/8BM3669/2008(H4N6)	CY080084	CY080083	CY080082	CY080080	CY080078	CY080081
A/northern pintail/Interior Alaska/8BM3723/2008(H4N1)	CY079676	CY079675	CY079674	CY079672	CY079670	CY079673
A/northern pintail/Interior Alaska/8MP0262R2/2008(H7N3)	CY080214	CY080213	CY080212	CY080210	CY080208	CY080211
A/northern pintail/Interior Alaska/9BM11789R0/2009(H3N8)	CY142857	CY142856	CY142855	CY142853	CY142851	CY142854
A/northern pintail/Interior Alaska/9BM12700R0/2009(H3N8)	CY143347	CY143346	CY143345	CY143343	CY143341	CY143344
A/northern pintail/Interior Alaska/9BM6144R0/2009(H3N8)	CY142593	CY142592	CY142591	CY142589	CY142587	CY142590
A/northern pintail/Interior Alaska/9BM9663R0/2009(H4N6)	CY136237	CY136236	CY136235	CY136233	CY136231	CY136234
A/northern pintail/Louisiana/Sg-01033/2008(H4N8)	CY140558	CY140557	CY140556	CY140554	CY140552	CY140555
A/northern pintail/Missouri/319/2009(H12N5)	CY097645	CY097644	CY097643	CY097641	CY097639	CY097642
A/northern pintail/Oregon/44249-559/2006(H3N8)	CY076244	CY076243	CY076242	CY076240	CY076238	CY076241
A/northern pintail/Texas/421716/2001(H1N1)	CY139656	CY139655	CY139654	CY139652	CY139650	CY139653
A/northern pintail/Texas/828189/2002(H6N1)	CY139903	CY139902	CY139901	CY139899	CY139897	CY139900
A/northern pintail/Texas/828191/2002(H6N1)	CY139920	CY139919	CY139918	CY139916	CY139914	CY139917
A/northern pintail/Utah/469648/2006(H5N2)	GQ923300	GQ923299	GQ923298	GQ923296	GQ923294	GQ923297
A/northern pintail/Wisconsin/10OS2857/2010(H5N2)	CY133100	CY133099	CY133098	CY133096	CY133094	CY133097
A/northern shoveler/Alaska/7MP1606/2007(H3N8)	CY044015	CY044014	CY044013	CY044011	CY044009	CY044012
A/northern shoveler/Alaska/7MP1668/2007(H3N8)	CY044031	CY044030	CY044029	CY044027	CY044025	CY044028
A/northern shoveler/Arkansas/12OS158/2012(H7N1)	CY167150	CY167149	CY167148	CY167146	CY167144	CY167147
A/northern shoveler/Arkansas/12OS160/2012(H10N3)	CY167158	CY167157	CY167156	CY167154	CY167152	CY167155
A/northern shoveler/Arkansas/AI09-6012/2009(H10N3)	CY141048	CY141047	CY141046	CY141044	CY141042	CY141045
A/northern shoveler/California/1628V/2013(H6N8)	KX351584	KX351760	KX351576	KX351709	KX351530	KX351725
A/northern shoveler/California/27820/2007(H7N3)	CY075988	CY075987	CY075986	CY075984	CY075982	CY075985
A/northern shoveler/California/27985/2007(H7N6)	CY076396	CY076395	CY076394	CY076392	CY076390	CY076393

A/northern shoveler/California/2810/2011(H11N2)	CY133908	CY133907	CY133906		CY133904		CY133902	CY133905
A/northern shoveler/California/3483/2010(H12N5)	CY120674	CY120673	CY120672		CY120670		CY120668	CY120671
A/northern shoveler/California/4020/2011(H4N3)	CY134310	CY134309	CY134308		CY134306		CY134304	CY134307
A/northern shoveler/California/44363-082/2007(H11N9)	CY076700	CY076699	CY076698		CY076696		CY076694	CY076697
A/northern shoveler/California/8675/2008(H11N9)	CY094300	CY094299	CY094298		CY094296		CY094294	CY094297
A/northern shoveler/California/9228/2008(H4N6)	CY094324	CY094323	CY094322		CY094320		CY094318	CY094321
A/northern shoveler/California/9781/2008(H1N3)	CY094340	CY094339	CY094338		CY094336		CY094334	CY094337
A/northern shoveler/California/HKWF1046C/2007(H3N5)	CY094364	CY094363	CY094362		CY094360		CY094358	CY094361
A/northern shoveler/California/K168/2005(H1N9)	CY053804	CY053803	CY053802		CY053800		CY053798	CY053801
A/Northern shoveler/Guatemala/CIP049H112-79/2012(H4N2)	KX960463	KX986855	KX960402		KX960442		KX960405	KX960406
A/Northern shoveler/Guatemala/CIP049HT114-14/2013(H3N8)	KX960470	KX986856	KX960427		KX960407		KX960385	KX960474
A/northern shoveler/Illinois/08OS3331/2008(H4N8)	CY079515	CY079514	CY079513		CY079511		CY079509	CY079512
A/northern shoveler/Illinois/10OS3619/2010(H11N9)	CY133036	CY133035	CY133034		CY133032		CY133030	CY133033
A/northern shoveler/Illinois/11OS4404/2011(H7N3)	CY167002	CY167001	CY167000		CY166998		CY166996	CY166999
A/northern shoveler/Illinois/11OS4707/2011(H4N6)	CY167027	CY167026	CY167025		CY167023		CY167021	CY167024
A/northern shoveler/Illinois/11OS5680/2011(H11N9)	CY166994	CY166993	CY166992		CY166990		CY166988	CY166991
A/northern shoveler/Illinois/12OS4951/2012(H7N3)	CY186755	CY186754	CY186753		CY186751		CY186749	CY186752
A/northern shoveler/Illinois/12OS5128/2012(H2N5)	CY186690	CY186689	CY186688		CY186686		CY186684	CY186687
A/northern shoveler/Illinois/12OS5138/2012(H1N1)	CY186698	CY186697	CY186696		CY186694		CY186692	CY186695
A/northern shoveler/Illinois/12OS5294/2012(H10N3)	CY186731	CY186730	CY186729		CY186727		CY186725	CY186728
A/northern shoveler/Interior Alaska/1/2007(H4N6)	CY039831	CY039832	CY039833		CY039835		CY039837	CY039838
A/northern shoveler/Interior Alaska/11BM03861/2011(H3N8)	CY196277	CY196276	CY196275		CY196273		CY196271	CY196274
A/northern shoveler/Interior Alaska/4/2007(H3N8)	CY039823	CY039824	CY039825		CY039827		CY039829	CY039830
A/northern shoveler/Interior Alaska/7MP1033/2007(H3N8)	CY077395	CY077394	CY077393		CY077391		CY077389	CY077392
A/northern shoveler/Interior Alaska/7MP1081/2007(H1N1)	CY077229	CY077228	CY077227		CY077225		CY077223	CY077226
A/northern shoveler/Minnesota/Sg-00612/2008(H10N7)	CY145484	CY145483	CY145482		CY145480		CY145478	CY145481
A/northern shoveler/Mississippi/09OS025/2009(H12N5)	CY079395	CY079394	CY079393		CY079391		CY079389	CY079392
A/northern shoveler/Mississippi/09OS168/2009(H10N6)	CY079403	CY079402	CY079401		CY079399		CY079397	CY079400
A/northern shoveler/Mississippi/10OS4526/2010(H6N2)	CY133313	CY133312	CY133311		CY133309		CY133307	CY133310
A/northern shoveler/Mississippi/11OS289/2011(H7N3)	CY133736	CY133735	CY133734		CY133732		CY133730	CY133733
A/northern shoveler/Mississippi/11OS5805/2011(H5N1)	CY166743	CY166742	CY166741		CY166739		CY166737	CY166740
A/northern shoveler/Mississippi/11OS5812/2011(H11N9)	CY166751	CY166750	CY166749		CY166747		CY166745	CY166748
A/northern shoveler/Mississippi/11OS5875/2011(H5N2)	CY166783	CY166782	CY166781		CY166779		CY166777	CY166780
A/northern shoveler/Mississippi/12OS362/2012(H7N7)	CY167231	CY167230	CY167229		CY167227		CY167225	CY167228
A/northern shoveler/Mississippi/252/2010(H10N7)	CY097677	CY097676	CY097675		CY097673		CY097671	CY097674
A/northern shoveler/Mississippi/397/2010(H1N3)	CY097741	CY097740	CY097739		CY097737		CY097735	CY097738
A/northern shoveler/Mississippi/A00682947/2008(H7N7)	KU290258	KU290257	KU290256		KU290254		KU290252	KU290255
A/northern shoveler/Missouri/10OS4632/2010(H7N7)	CY133372	CY133371	CY133370		CY133368		CY133366	CY133369
A/northern shoveler/Missouri/10OS4718/2010(H10N7)	CY133407	CY133406	CY133405		CY133403		CY133401	CY133404
A/northern shoveler/Missouri/10OS4750/2010(H7N3)	CY133423	CY133422	CY133421		CY133419		CY133417	CY133420
A/northern shoveler/Missouri/12OS5755/2012(H10N3)	CY186771	CY186770	CY186769		CY186767		CY186765	CY186768
A/northern shoveler/Missouri/196/2009(H10N3)	CY097621	CY097620	CY097619		CY097617		CY097615	CY097618
A/Northern shoveler/NC/6412-052/2005(H7N6)	GU186481	GU186480	GU186479		GU186477		GU186475	GU186478
A/northern shoveler/Nevada/A00505416/2008(H7N6)	KU290266	KU290265	KU290264		KU290262		KU290260	KU290263

A/northern shoveler/Ohio/12OS3173/2012(H12N5)	CY186966	CY186965	CY186964		CY186962		CY186960	CY186963
A/northern shoveler/Ohio/12OS5647/2012(H2N3)	CY186996	CY186995	CY186994		CY186992		CY186990	CY186993
A/northern shoveler/Oregon/44336-179/2007(H4N6)	CY076476	CY076475	CY076474		CY076472		CY076470	CY076473
A/northern shoveler/Oregon/A00654616/2008(H7N3)	KU290282	KU290281	KU290280		KU290278		KU290276	KU290279
A/northern shoveler/TX/56/2000(H10N7)	EU743329	EU743328	EU743327		EU743325		EU743323	EU743326
A/northern shoveler/Washington/44249-603/2006(H6N1)	CY076252	CY076251	CY076250		CY076248		CY076246	CY076249
A/northern shoveler/Washington/44249-645/2006(H5N2)	CY076260	CY076259	CY076258		CY076256		CY076254	CY076257
A/northern shoveler/Washington/44249-664/2006(H7N3)	CY076268	CY076267	CY076266		CY076264		CY076262	CY076265
A/northern shoveler/Washington/44249-675/2006(H10N2)	CY076276	CY076275	CY076274		CY076272		CY076270	CY076273
A/northern shoveler/Washington/44249-700/2006(H10N1)	CY076284	CY076283	CY076282		CY076280		CY076278	CY076281
A/northern shoveler/Wisconsin/11OS3013/2011(H4N8)	CY166564	CY166563	CY166562		CY166560		CY166558	CY166561
A/northern shoverl/Mississippi/11OS145/2011(H7N9)	CY133656	CY133655	CY133654		CY133652		CY133650	CY133653
A/pintail duck/Alberta/210/2002(H1N1)		CY004552	CY004551		CY004549		CY004547	CY004550
A/pintail/Alaska/310/2005(H4N6)	CY017748	CY017747	CY017746		CY017744		CY017742	CY017745
A/pintail/Alaska/49/2005(H3N8)	CY020884	CY020883	CY020882		CY020880		CY020878	CY020881
A/pintail/Alaska/53/2005(H3N6)	CY013270	CY013269	CY013268		CY013266		CY013264	CY013267
A/pintail/Alberta/166/2003(H3N6)	CY102759	CY102758	CY102757		CY102755		CY102753	CY102756
A/pintail/Alberta/202/2000(H10N7)	CY005249	CY005248	CY005247		CY005245		CY005243	CY005246
A/pintail/Alberta/210/2002(H1N1)	KF424111							
A/quail/New York/30732-12/2005(H7N2)	CY031738	CY031737	CY031736		CY031734		CY031732	CY031735
A/quail/New York/501360/2007(H5N2)	GQ923556	GQ923555	GQ923554		GQ923552		GQ923550	GQ923553
A/quail/New York/63806-13/2005(H7N2)	CY033320	CY033319	CY033318		CY033316		CY033314	CY033317
A/quail/New York/89641/2005(H7N2)	CY034397	CY034396	CY034395		CY034393		CY034391	CY034394
A/red knot/DE/1587/2001(H10N7)	EU743337	EU743336	EU743335		EU743333		EU743331	EU743334
A/red knot/Delaware/650665/2002(H7N3)	CY144315	CY144314	CY144313		CY144311		CY144309	CY144312
A/red knot/Delaware/650666/2002(H11N9)	CY144339	CY144338	CY144337		CY144335		CY144333	CY144336
A/red knot/New Jersey/828235/2001(H6N5)	CY144372	CY144371	CY144370		CY144368		CY144366	CY144369
A/red knot/New Jersey/AI06-096/2006(H7N3)	CY144682	CY144681	CY144680		CY144678		CY144676	CY144679
A/red knot/New Jersey/Sg-00478/2008(H12N5)	CY038053	CY038054	CY038055		CY144952		CY144950	CY144953
A/redhead/Alberta/458/2007(H3N8)	CY103458	CY103457	CY103456		CY103454		CY103452	CY103455
A/redhead/Ohio/13OS0428/2013(H6N2)	CY187060	CY187059	CY187058		CY187056		CY187054	CY187057
A/red-necked grebe/Minnesota/AI10-2528/2010(H4N8)	CY141112	CY141111	CY141110		CY141108		CY141106	CY141109
A/ring-billed gull/Quebec/G018/2010(H1N3)	CY138104	CY138103	CY138102		CY138100		CY138098	CY138101
A/ring-necked duck/Minnesota/Sg-01066/2008(H12N8)	CY140622	CY140621	CY140620		CY140618		CY140616	CY140619
A/ring-necked duck/New Brunswick/03449/2009(H11N9)	CY125356	CY125355	CY125354		CY125352		CY125350	CY125353
A/ring-necked duck/Texas/A00766403/2009(H7N1)	KU290331	KU290330	KU290329		KU290327		KU290325	KU290328
A/ruddy turnstone/DE/97/2000(H12N5)	CY005371	CY005370	CY005369		CY005367		CY005365	CY005368
A/ruddy turnstone/Delaware Bay/108/2007(H7N3)	CY036782	CY036781	CY036780		CY036778		CY036776	CY036779
A/ruddy turnstone/Delaware Bay/136/2007(H6N1)	CY127790	CY127789	CY127788		CY127786		CY127784	CY127787
A/ruddy turnstone/Delaware Bay/173/2007(H6N2)	CY127798	CY127797	CY127796		CY127794		CY127792	CY127795
A/ruddy turnstone/Delaware Bay/262/2006(H7N3)	CY039334	CY039333	CY039332		CY039330		CY039328	CY039331
A/ruddy turnstone/Delaware/103/2007(H5N1)	CY077117	CY077116	CY077115		CY077113		CY077111	CY077114
A/ruddy turnstone/Delaware/291/2006(H6N1)	CY047026	CY047025	CY047024		CY047022		CY047020	CY047023
A/ruddy turnstone/Delaware/650612/2002(H11N3)	CY144356	CY144355	CY144354		CY144352		CY144350	CY144353

A/ruddy turnstone/Delaware/828199/2001(H12N7)	CY144259	CY144258	CY144257	CY144255	CY144253	CY144256
A/ruddy turnstone/Delaware/AI00-1538/2000(H7N9)	CY186017	CY186016	CY186015	CY186013	CY186011	CY186014
A/ruddy turnstone/Delaware/AI03-118/2003(H12N5)	CY144538	CY144537	CY144536	CY144534	CY144532	CY144535
A/ruddy turnstone/Delaware/AI03-371/2003(H5N2)	CY144514	CY144513	CY144512	CY144510	CY144508	CY144511
A/ruddy turnstone/Delaware/AI09-1268/2009(H10N7)	CY146367	CY146366	CY146365	CY146363	CY146361	CY146364
A/ruddy turnstone/Delaware/AI09-879/2009(H10N7)	CY146196	CY146195	CY146194	CY146192	CY146190	CY146193
A/ruddy turnstone/Ilha de Canelas/A08/2008(H11N9)	KT932362	KT932363	KT932364	KT932365	KT932366	KT932367
A/ruddy turnstone/Ilha de Canelas/A17/2008(H11N9)	KT932368	KT932369	KT932370	KT932371	KT932372	KT932373
A/ruddy turnstone/Ilha de Canelas/A51/2008(H11N9)	KT932374	KT932375	KT932376	KT932377	KT932378	KT932379
A/ruddy turnstone/New Jersey/1148668/2004(H10N8)	CY144859	CY144858	CY144857	CY144855	CY144853	CY144856
A/ruddy turnstone/New Jersey/1148669/2004(H10N7)	CY144867	CY144866	CY144865	CY144863	CY144861	CY144864
A/ruddy turnstone/New Jersey/1148676/2004(H5N7)	CY144586	CY144585	CY144584	CY144582	CY144580	CY144583
A/ruddy turnstone/New Jersey/1321410/2005(H11N8)	CY144618	CY144617	CY144616	CY144614	CY144612	CY144615
A/ruddy turnstone/New Jersey/650615/2002(H11N2)	CY144364	CY144363	CY144362	CY144360	CY144358	CY144361
A/ruddy turnstone/New Jersey/650663/2002(H1N9)	CY144436	CY144435	CY144434	CY144432	CY144430	CY144433
A/ruddy turnstone/New Jersey/828227/2001(H5N8)	CY144243	CY144242	CY144241	CY144239	CY144237	CY144240
A/ruddy turnstone/New Jersey/AI05-1058/2005(H11N6)	CY144602	CY144601	CY144600	CY144598	CY144596	CY144599
A/ruddy turnstone/New Jersey/AI06-582/2006(H6N7)	CY144674	CY144673	CY144672	CY144670	CY144668	CY144671
A/ruddy turnstone/New Jersey/AI07-296/2007(H6N1)	CY144762	CY144761	CY144760	CY144758	CY144756	CY144759
A/ruddy turnstone/New Jersey/AI07-697/2007(H12N5)	CY144722	CY144721	CY144720	CY144718	CY144716	CY144719
A/ruddy turnstone/New Jersey/AI07-699/2007(H5N1)	CY144754	CY144753	CY144752	CY144750	CY144748	CY144751
A/ruddy turnstone/New Jersey/AI07-839/2007(H12N1)	CY144714	CY144713	CY144712	CY144710	CY144708	CY144711
A/ruddy turnstone/New Jersey/AI09-027/2009(H11N1)	CY145865	CY145864	CY145863	CY145861	CY145859	CY145862
A/ruddy turnstone/New Jersey/AI09-1292/2009(H1N1)	CY146383	CY146382	CY146381	CY146379	CY146377	CY146380
A/ruddy turnstone/New Jersey/AI09-151/2009(H11N1)	CY145890	CY145889	CY145888	CY145886	CY145884	CY145887
A/ruddy turnstone/New Jersey/AI09-169/2009(H11N8)	CY145906	CY145905	CY145904	CY145902	CY145900	CY145903
A/ruddy turnstone/New Jersey/AI09-194/2009(H11N8)	CY145914	CY145913	CY145912	CY145910	CY145908	CY145911
A/ruddy turnstone/New Jersey/AI09-256/2009(H1N1)	CY145930	CY145929	CY145928	CY145926	CY145924	CY145927
A/ruddy turnstone/New Jersey/AI09-305/2009(H10N7)	CY145954	CY145953	CY145952	CY145950	CY145948	CY145951
A/ruddy turnstone/New Jersey/AI09-372/2009(H6N1)	CY145978	CY145977	CY145976	CY145974	CY145972	CY145975
A/ruddy turnstone/New Jersey/AI09-377/2009(H6N1)	CY145986	CY145985	CY145984	CY145982	CY145980	CY145983
A/ruddy turnstone/New Jersey/AI09-727/2009(H1N9)	CY146132	CY146131	CY146130	CY146128	CY146126	CY146129
A/ruddy turnstone/New Jersey/AI09-961/2009(H10N7)	CY146245	CY146244	CY146243	CY146241	CY146239	CY146242
A/ruddy turnstone/New Jersey/AI11-2098/2011(H7N3)	CY185968	CY185967	CY185966	CY185964	CY185962	CY185965
A/ruddy turnstone/New Jersey/Sg-00476/2008(H12N5)	CY038043	CY038044	CY038045	CY144936	CY144934	CY144937
A/ruddy turnstone/New Jersey/Sg-00482/2008(H6N8)	CY144980	CY042423	CY042424	CY144976	CY144974	CY144977
A/ruddy turnstone/New Jersey/Sg-00496/2008(H4N6)	CY038113	CY038114	CY038115	CY145065	CY145063	CY145066
A/ruddy turnstone/New Jersey/Sg-00552/2008(H4N6)	CY038213	CY038214	CY038215	CY145415	CY145413	CY145416
A/ruddy turnstone/New Jersey/Sg-00561/2008(H11N9)	CY038233	CY038234	CY145692	CY145690	CY145688	CY145691
A/ruddy turnstone/New Jersey/Sg-00563/2008(H10N7)	CY038238	CY038239	CY145708	CY145706	CY145704	CY145707
A/ruddy turnstone/NJ/238/2004(H10N7)	EU743401	EU743400	EU743399	EU743397	EU743395	EU743398
A/sanderling/NJ/1042/2005(H3N6)	EU871867	EU871866	EU871865	EU871863	EU871861	EU871864
A/snow goose/Delaware/12OS3766/2012(H1N1)	CY202776	CY202775	CY202774	CY202772	CY202770	CY202773
A/snow goose/Maryland/353/2005(H6N1)	CY018916	CY018915	CY018914	CY018912	CY018910	CY018913

A/snow goose/Maryland/410/2005(H6N1)	CY017796	CY017795	CY017794		CY017792		CY017790	CY017793
A/snow goose/Montana/466771-4/2006(H5N2)	GQ923268	GQ923267	GQ923266		GQ923264		GQ923262	GQ923265
A/thick-billed murre/Canada/1813/2011(H12N5)	CY149627	CY149626	CY149625		CY149623		CY149621	CY149624
A/thick-billed murre/Canada/1871/2011(H1N1)	CY149635	CY149634	CY149633		CY149631		CY149629	CY149632
A/tundra swan/Alaska/450959/2006(H5N2)	GQ923468	GQ923467	GQ923466		GQ923464		GQ923462	GQ923465
A/turkey/Minnesota/42782-1/2013(H6N8)	KM244065	KM244066	KM244067		KM244069		KM244071	KM244072
A/turkey/New York/23165-8/2005(H7N2)	CY095019	CY095018	CY095017		CY095015		CY095013	CY095016
A/turkey/VA/158512/2002(H7N2)	EU743265	EU743264	EU743263		EU743262		AY241622	AY241659
A/turkey/VA/505477-18/2007(H5N1)	GU186516	GU186515	GU186514		GU186512		GU186510	GU186513
A/western grebe/Washington/20569-004/2007(H1N2)	CY076164	CY076163	CY076162		CY076160		CY076158	CY076161
A/white-faced whistling duck/Colombia/1/2011(H5N2)	CY207188	CY207187	CY207186		CY207184		CY207182	CY207185
A/white-winged scoter/Maryland/301/2005(H4N6)	CY021900	CY021899	CY021898		CY021896		CY021894	CY021897
A/wild duck/Ohio/623/2004(H5N1)	GU186792	GU186791	GU186790		GU186788		GU186786	GU186789
A/wood duck/Ohio/623/2004(H5N1)	CY053332	CY053331	CY053330		CY053328		CY053326	CY053329
A/wood duck/Wisconsin/11OS2912/2011(H3N6)	CY167117	CY167116	CY167115		CY167113		CY167111	CY167114

Supplementary Tables 2-6. Viral dissemination of IAVs among flyways, taxonomic groups, US regions, habitats and DHPs. Percentage of Markov jump counts for each discrete trait for all internal segments. Shading represents relative intensity of viral flow between state pairs (green: low; red: high).

Supplementary Table 2. Percentage of Markov jump counts for the Flyway trait

**PB2**	DHP1	DHP2	DHP3	DHP4	DHP5	DHP6	DHP7
Atlantic	6.54	2.55	0.03	2.45	1.44	1.60	1.37
DHP1	0.00	0.20	0.01	0.07	0.61	0.48	0.01
DHP2	0.14	0.00	0.01	0.00	0.00	0.00	0.01
Central	0.00	0.00	0.04	0.00	0.00	0.00	0.01
DHP3	0.00	0.00	0.00	0.00	0.00	0.00	0.00
Latin America	0.00	0.00	0.01	0.00	0.00	0.00	0.00
DHP4	0.14	0.00	0.00	0.00	1.27	0.00	0.00
DHP5	0.01	0.00	0.01	0.13	0.00	0.01	0.00
Mississippi	3.20	0.00	0.08	0.04	0.01	1.29	0.01
Pacific	0.00	0.01	0.05	0.00	0.00	0.00	0.00
DHP6	0.01	0.00	0.03	0.00	0.00	0.00	0.00
DHP7	0.00	0.03	0.01	0.00	0.00	0.00	0.00

**PB1**	DHP1	DHP2	DHP3	DHP4	DHP5	DHP6	DHP7
Atlantic	4.90	0.08	0.15	1.85	0.01	0.71	0.12
DHP1	0.00	2.09	0.03	0.01	1.75	0.19	1.44
DHP2	0.08	0.00	0.00	0.00	0.05	0.01	0.01
Central	0.04	0.01	0.05	0.01	0.10	0.00	0.00
DHP3	0.00	0.00	0.00	0.00	0.00	0.00	0.00
Latin America	0.00	0.00	0.00	0.00	0.00	0.00	0.00
DHP4	0.00	0.00	0.01	0.00	1.75	0.00	0.00
DHP5	0.01	0.07	0.01	0.07	0.00	0.00	0.00
Mississippi	6.89	0.64	0.03	1.03	0.91	2.69	0.27
Pacific	0.01	0.00	0.01	0.00	0.00	0.00	0.02
DHP6	0.00	0.01	0.03	0.00	0.00	0.00	0.00
DHP7	0.04	0.00	0.01	0.00	0.00	0.01	0.00

**PA EA**	DHP1	DHP2	DHP3	DHP4	DHP5	DHP6	DHP7
Atlantic	2.57	0.30	0.03	0.91	0.45	2.46	0.30
DHP1	0.00	3.33	0.03	1.65	2.32	0.00	1.28
DHP2	0.04	0.00	0.00	0.00	0.00	0.00	0.01
Central	0.19	0.01	0.07	0.01	0.44	0.00	0.05
DHP3	0.00	0.00	0.00	0.00	0.00	0.00	0.00
Latin America	0.00	0.00	0.02	0.00	0.00	0.00	0.00
DHP4	0.02	0.00	0.01	0.00	0.74	0.00	0.01
DHP5	0.05	0.00	0.00	1.23	0.00	0.00	0.02
Mississippi	7.54	0.06	0.21	0.26	0.05	2.89	0.20
Pacific	0.01	0.00	0.04	0.00	0.00	0.01	0.00
DHP6	0.01	0.01	0.06	0.01	0.01	0.00	0.01
DHP7	0.00	0.00	0.01	0.01	0.00	0.00	0.00

**PA NA**	DHP1	DHP2		DHP4	DHP5		DHP7
Atlantic	3.00	0.21		1.13	0.61		0.48
DHP1	0.00	0.01		0.09	0.37		0.25
DHP2	0.00	0.00		0.00	0.10		0.01
Central	0.01	0.08		0.22	0.03		0.02
Latin America	0.00	0.00		0.00	0.00		0.00
DHP4	0.00	0.00		0.00	0.01		0.04
DHP5	0.01	0.82		0.01	0.00		0.02
Mississippi	8.11	0.15		0.28	1.50		0.04
Pacific	0.01	0.43		0.02	0.03		0.02
DHP7	0.01	0.00		0.00	0.00		0.00

**NS A**	DHP1	DHP2	DHP3	DHP4	DHP5	DHP6	DHP7
Atlantic	9.21	2.04	0.10	2.93	3.02	2.15	1.52
DHP1	0.00	0.24	0.04	0.00	0.51	0.00	0.09
DHP2	0.44	0.00	0.02	0.02	0.01	0.00	0.00
Central	0.19	0.00	0.01	0.00	0.00	0.00	0.00
DHP3	0.00	0.00	0.00	0.00	0.00	0.00	0.00
Latin America	0.01	0.00	0.01	0.00	0.00	0.00	0.00
DHP4	0.00	0.17	0.04	0.00	0.90	0.00	0.00
DHP5	0.01	0.08	0.03	0.54	0.00	0.00	0.01
Mississippi	0.92	0.00	0.02	0.00	0.00	0.01	0.01
Pacific	0.06	0.00	0.04	0.00	0.00	0.00	0.01
DHP6	0.00	0.00	0.06	0.00	0.00	0.00	0.00
DHP7	0.00	0.01	0.05	0.00	0.00	0.00	0.00

**NS B**	DHP1	DHP2		DHP4	DHP5	DHP6	DHP7
Atlantic	0.43	0.31		0.04	0.01	3.53	0.02
DHP1	0.00	2.70		0.02	0.71	0.98	0.03
DHP2	0.49	0.00		0.01	0.02	0.06	0.01
Central	0.10	0.08		1.15	0.02	0.02	0.06
Latin America	0.00	0.00		0.00	0.01	0.01	0.00
DHP4	0.00	0.00		0.00	0.00	0.00	0.14
DHP5	0.12	0.00		0.01	0.00	0.03	0.01
Mississippi	7.03	0.80		0.65	0.98	0.57	0.80
Pacific	1.02	0.04		0.04	1.03	0.08	0.04
DHP6	0.05	0.04		0.02	0.01	0.00	0.01
DHP7	0.00	0.00		0.78	0.00	0.01	0.00

**NP**	DHP1	DHP2	DHP3	DHP4	DHP5	DHP6	DHP7
Atlantic	5.41	1.27	0.08	1.63	1.14	2.67	1.37
DHP1	0.00	0.37	0.03	0.25	1.28	0.01	0.07
DHP2	0.38	0.00	0.01	0.00	0.02	0.00	0.00
Central	0.01	0.00	0.01	0.00	0.00	0.00	0.00
DHP3	0.00	0.00	0.00	0.00	0.00	0.00	0.00
Latin America	0.00	0.00	0.00	0.00	0.00	0.00	0.00
DHP4	0.19	0.00	0.01	0.00	1.05	0.00	0.00
DHP5	0.01	0.09	0.01	0.24	0.00	0.00	0.00
Mississippi	5.00	0.64	0.02	0.67	0.08	0.77	0.07
Pacific	0.01	0.00	0.01	0.00	0.00	0.00	0.00
DHP6	0.00	0.00	0.06	0.00	0.00	0.00	0.03
DHP7	0.01	0.00	0.03	0.00	0.00	0.01	0.00

**MP**	DHP1	DHP2	DHP3	DHP4	DHP5	DHP6	DHP7
Atlantic	8.57	1.69	0.05	1.83	2.48	1.77	0.32
DHP1	0.00	0.18	0.19	0.11	0.28	0.05	0.91
DHP2	0.69	0.00	0.00	0.00	0.02	0.01	0.00
Central	0.02	0.01	0.00	0.00	0.04	0.00	0.02
DHP3	0.00	0.00	0.00	0.00	0.00	0.00	0.00
Latin America	0.00	0.00	0.00	0.00	0.00	0.00	0.00
DHP4	0.03	0.00	0.01	0.00	0.62	0.00	0.00
DHP5	0.02	0.02	0.01	0.36	0.00	0.00	0.00
Mississippi	3.19	0.73	0.00	0.92	0.38	1.70	0.05
Pacific	0.03	0.01	0.00	0.00	0.06	0.00	0.13
DHP6	0.00	0.11	0.01	0.00	0.00	0.00	0.00
DHP7	0.01	0.00	0.01	0.00	0.00	0.00	0.00

Supplementary Table 3. Percentage of Markov jump counts for the Region trait

**PB2**	DHP1	DHP2	DHP3	DHP4	DHP5	DHP6	DHP7
DHP2	0.06	0.00	0.01	0.00	0.00	0.00	0.00
Brazil	0.00	0.00	0.00	0.00	0.00	0.00	0.00
Canada	0.01	0.00	0.01	0.00	0.00	0.00	0.00
DHP1	0.00	0.38	0.01	0.21	0.55	0.48	0.09
Chile	0.00	0.00	0.00	0.00	0.00	0.00	0.00
Colombia	0.00	0.00	0.00	0.00	0.00	0.00	0.00
East North Central	2.17	0.00	0.01	0.02	0.00	1.30	0.00
East South Central	0.11	0.00	0.01	0.00	0.00	0.00	0.00
DHP3	0.00	0.00	0.00	0.00	0.00	0.00	0.00
Guatemala	0.00	0.00	0.01	0.00	0.00	0.00	0.00
DHP4	0.08	0.00	0.01	0.00	1.00	0.00	0.00
DHP5	0.00	0.00	0.01	0.05	0.00	0.00	0.00
Mexico	0.00	0.00	0.00	0.00	0.00	0.00	0.00
Mid-Atlantic	0.01	0.00	0.01	0.02	0.00	0.00	0.00
Mountain	0.02	0.00	0.01	0.00	0.00	0.00	0.00
New England	0.00	0.00	0.00	0.00	0.00	0.00	0.00
Pacific	0.02	0.01	0.01	0.00	0.01	0.00	0.00
DHP6	0.01	0.00	0.01	0.00	0.00	0.00	0.00
South Atlantic	5.16	1.48	0.03	1.64	0.71	0.53	0.89
DHP7	0.00	0.03	0.01	0.00	0.00	0.00	0.00
West North Central	0.02	0.00	0.04	0.01	0.01	0.00	0.00
West South Central	0.00	0.01	0.01	0.00	0.00	0.00	0.00

**PB1**	DHP1	DHP2	DHP3	DHP4	DHP5	DHP6	DHP7
DHP2	0.02	0.00	0.01	0.00	0.02	0.00	0.00
Brazil	0.00	0.00	0.00	0.00	0.00	0.00	0.00
Canada	0.02	0.00	0.02	0.00	0.03	0.00	0.05
DHP1	0.00	1.80	0.05	0.56	1.64	0.91	1.06
Chile	0.00	0.00	0.00	0.00	0.00	0.00	0.00
Colombia	0.00	0.00	0.00	0.00	0.00	0.00	0.00

East North Central	0.52	0.01	0.00	0.35	0.01	0.75	0.00
East South Central	0.01	0.00	0.00	0.09	0.00	0.00	0.00
DHP3	0.02	0.00	0.00	0.00	0.00	0.00	0.00
Guatemala	0.00	0.00	0.00	0.01	0.00	0.00	0.00
DHP4	0.00	0.00	0.01	0.00	0.89	0.00	0.00
DHP5	0.00	0.02	0.01	0.27	0.00	0.00	0.00
Mexico	0.00	0.00	0.00	0.00	0.00	0.00	0.00
Mid-Atlantic	0.90	0.00	0.02	0.35	0.04	0.00	0.00
Mountain	0.01	0.00	0.01	0.00	0.00	0.00	0.00
New England	0.00	0.00	0.00	0.00	0.00	0.00	0.00
Pacific	0.05	0.00	0.00	0.00	0.01	0.00	0.00
DHP6	0.00	0.00	0.01	0.00	0.00	0.00	0.00
South Atlantic	2.53	0.00	0.02	0.28	0.01	0.42	0.03
DHP7	0.02	0.00	0.01	0.00	0.00	0.00	0.00
West North Central	3.92	0.00	0.01	0.01	0.19	0.19	0.05
West South Central	1.43	0.00	0.01	0.01	0.00	0.01	0.00

**PA EA**	DHP1	DHP2	DHP3	DHP4	DHP5	DHP6	DHP7
DHP2	0.02	0.00	0.00	0.00	0.00	0.00	0.01
Brazil	0.00	0.00	0.00	0.00	0.00	0.00	0.00
Canada	0.03	0.00	0.03	0.01	0.00	0.21	0.01
DHP1	0.00	2.38	0.01	0.87	1.74	0.00	0.98
Chile	0.00	0.00	0.00	0.00	0.00	0.00	0.00
East North Central	4.85	0.13	0.03	0.49	0.02	2.91	0.23
East South Central	0.01	0.02	0.01	0.00	0.00	0.00	0.00
DHP3	0.00	0.00	0.00	0.00	0.00	0.00	0.00
Guatemala	0.00	0.00	0.00	0.00	0.00	0.03	0.00
DHP4	0.02	0.00	0.01	0.00	0.38	0.00	0.01
DHP5	0.18	0.00	0.00	1.05	0.00	0.00	0.01
Mexico	0.00	0.00	0.00	0.00	0.00	0.00	0.00
Mid-Atlantic	0.38	0.00	0.00	0.09	0.22	0.00	0.00
Mountain	0.00	0.00	0.00	0.00	0.00	0.02	0.00

New England	0.00	0.05	0.01	0.00	0.00	0.00	0.00
Pacific	0.02	0.00	0.01	0.00	0.00	0.00	0.00
DHP6	0.01	0.00	0.02	0.00	0.00	0.00	0.01
South Atlantic	0.71	0.01	0.13	0.35	0.08	0.61	0.04
DHP7	0.00	0.00	0.00	0.00	0.00	0.00	0.00
West North Central	1.89	0.00	0.05	0.06	0.28	0.02	0.01
West South Central	0.12	0.00	0.01	0.01	0.07	0.00	0.00

**PA NA**	DHP1	DHP2	DHP4	DHP5	DHP7
DHP2	0.00	0.00	0.01	0.03	0.00
Canada	0.02	0.00	0.00	0.00	0.00
DHP1	0.00	0.00	0.04	0.29	0.15
Colombia	0.00	0.00	0.00	0.00	0.00
East North Central	5.58	0.03	0.75	0.60	0.01
East South Central	0.02	0.01	0.01	0.00	0.00
Guatemala	0.00	0.00	0.00	0.00	0.00
DHP4	0.00	0.00	0.00	0.01	0.00
DHP5	0.01	0.49	0.00	0.00	0.01
Mid-Atlantic	1.08	0.01	0.00	0.35	0.30
Mountain	0.00	0.06	0.00	0.00	0.00
New England	0.05	0.00	0.00	0.00	0.00
Pacific	0.06	0.34	0.01	0.01	0.00
South Atlantic	0.35	0.03	0.21	0.16	0.02
DHP7	0.01	0.00	0.00	0.01	0.00
West North Central	0.10	0.04	0.01	0.01	0.00
West South Central	0.01	0.01	0.00	0.00	0.00

**NS A**	DHP1	DHP2	DHP3	DHP4	DHP5	DHP6	DHP7
DHP2	0.19	0.00	0.00	0.02	0.02	0.00	0.00
Brazil	0.00	0.00	0.00	0.00	0.00	0.00	0.00
Canada	0.68	0.00	0.01	0.03	0.02	0.03	0.00
DHP1	0.00	0.82	0.01	0.18	0.74	0.04	0.17

Chile	0.00	0.00	0.00	0.00	0.00	0.00	0.00
Colombia	0.00	0.00	0.00	0.00	0.00	0.00	0.00
East North Central	1.53	0.04	0.01	0.12	0.02	0.13	0.03
East South Central	0.13	0.00	0.00	0.01	0.00	0.00	0.00
DHP3	0.00	0.00	0.00	0.00	0.00	0.00	0.00
Guatemala	0.01	0.00	0.00	0.00	0.00	0.00	0.00
DHP4	0.02	0.17	0.02	0.00	0.88	0.01	0.02
DHP5	0.03	0.09	0.01	0.55	0.00	0.00	0.03
Mexico	0.00	0.00	0.00	0.00	0.00	0.00	0.00
Mid-Atlantic	3.49	0.55	0.06	1.09	1.29	0.46	0.61
Mountain	0.00	0.00	0.00	0.00	0.00	0.00	0.00
New England	0.01	0.00	0.00	0.00	0.00	0.00	0.00
Pacific	0.02	0.00	0.00	0.01	0.01	0.00	0.00
DHP6	0.01	0.00	0.02	0.00	0.00	0.00	0.00
South Atlantic	1.73	0.12	0.09	0.47	0.16	0.77	0.24
DHP7	0.01	0.01	0.01	0.00	0.01	0.00	0.00
West North Central	0.19	0.00	0.00	0.03	0.01	0.00	0.00
West South Central	0.27	0.00	0.00	0.00	0.02	0.00	0.00

**NS B**	DHP1	DHP2		DHP4	DHP5	DHP6	DHP7
DHP2	0.31	0.00		0.01	0.01	0.04	0.00
Canada	0.05	0.04		0.02	0.01	0.21	0.00
DHP1	0.00	1.91		0.02	0.64	0.81	0.00
East North Central	0.50	0.18		0.11	0.07	1.27	0.14
East South Central	0.01	0.00		0.01	0.01	0.01	0.00
Guatemala	0.00	0.00		0.00	0.00	0.01	0.00
DHP4	0.00	0.00		0.00	0.00	0.00	0.05
DHP5	0.07	0.00		0.01	0.00	0.02	0.00
Mexico	0.00	0.00		0.00	0.00	0.00	0.00
Mid-Atlantic	0.18	0.02		0.01	0.00	0.13	0.00
Mountain	0.01	0.00		0.00	0.02	0.01	0.00
New England	0.03	0.01		0.02	0.00	0.02	0.01

Pacific	4.72	0.10		0.07	1.00	0.12	0.01
DHP6	0.04	0.01		0.02	0.01	0.00	0.01
South Atlantic	0.19	0.19		0.06	0.01	0.32	0.06
DHP7	0.00	0.00		0.59	0.00	0.00	0.00
West North Central	0.18	0.14		0.15	0.06	0.59	0.48
West South Central	0.21	0.07		0.76	0.05	0.03	0.02

**NP**	DHP1	DHP2	DHP3	DHP4	DHP5	DHP6	DHP7
DHP2	0.02	0.00	0.00	0.00	0.01	0.00	0.00
Brazil	0.00	0.00	0.01	0.00	0.00	0.00	0.00
Canada	0.01	0.00	0.01	0.00	0.00	0.00	0.00
DHP1	0.00	1.56	0.01	1.15	1.61	1.50	0.98
Chile	0.00	0.00	0.00	0.00	0.00	0.00	0.00
Colombia	0.00	0.00	0.00	0.00	0.00	0.00	0.00
East North Central	2.93	0.05	0.00	0.07	0.00	0.63	0.01
East South Central	0.01	0.00	0.00	0.32	0.00	0.00	0.00
DHP3	0.00	0.00	0.00	0.00	0.00	0.00	0.00
Guatemala	0.00	0.00	0.00	0.00	0.00	0.00	0.00
DHP4	0.02	0.00	0.01	0.00	0.78	0.00	0.00
DHP5	0.01	0.02	0.00	0.19	0.00	0.00	0.00
Mexico	0.00	0.00	0.00	0.00	0.00	0.00	0.00
Mid-Atlantic	0.32	0.01	0.04	0.11	0.03	0.00	0.00
Mountain	0.00	0.00	0.01	0.00	0.00	0.00	0.00
New England	0.01	0.00	0.01	0.00	0.00	0.00	0.00
Pacific	0.02	0.00	0.01	0.00	0.00	0.01	0.00
DHP6	0.01	0.00	0.01	0.00	0.00	0.00	0.00
South Atlantic	3.05	0.02	0.05	0.16	0.01	0.07	0.00
DHP7	0.00	0.00	0.01	0.00	0.00	0.00	0.00
West North Central	4.45	0.01	0.01	0.00	0.00	0.00	0.00
West South Central	0.01	0.00	0.01	0.00	0.00	0.00	0.00

**MP**	DHP1	DHP2	DHP3	DHP4	DHP5	DHP6	DHP7
DHP2	0.12	0.00	0.01	0.00	0.00	0.02	0.00
Brazil	0.00	0.00	0.00	0.00	0.00	0.00	0.00
Canada	2.20	0.00	0.00	0.00	0.00	0.00	0.00
DHP1	0.00	1.93	0.07	0.32	1.32	0.61	0.88
Chile	0.00	0.00	0.00	0.00	0.00	0.00	0.00
Colombia	0.00	0.00	0.00	0.00	0.00	0.00	0.00
East North Central	0.35	0.01	0.00	0.56	0.01	1.63	0.00
East South Central	0.01	0.00	0.00	0.05	0.00	0.00	0.00
DHP3	0.00	0.00	0.00	0.00	0.00	0.00	0.00
Guatemala	0.01	0.00	0.00	0.00	0.00	0.00	0.00
DHP4	0.03	0.00	0.01	0.00	0.46	0.00	0.00
DHP5	0.01	0.01	0.01	0.32	0.00	0.00	0.00
Mexico	0.00	0.00	0.00	0.00	0.00	0.00	0.00
Mid-Atlantic	0.19	0.00	0.00	0.46	0.42	0.00	0.00
Mountain	0.00	0.00	0.00	0.00	0.00	0.00	0.00
New England	0.00	0.00	0.00	0.00	0.00	0.00	0.00
Pacific	1.11	0.00	0.00	0.00	0.06	0.00	0.04
DHP6	0.04	0.04	0.00	0.00	0.00	0.00	0.00
South Atlantic	1.68	0.00	0.09	0.42	0.01	0.04	0.00
DHP7	0.00	0.00	0.01	0.00	0.00	0.00	0.00
West North Central	2.82	0.02	0.00	0.05	0.29	0.00	0.01
West South Central	0.43	0.00	0.00	0.01	0.03	0.00	0.00

Supplementary Table 4. Percentage of Markov jump counts for the Habitat trait

**PB2**	DHP1	DHP2	DHP3	DHP4	DHP5	DHP6	DHP7
DHP1	0.00	0.65	0.03	0.05	0.98	0.75	0.03
DHP2	0.17	0.00	0.01	0.00	0.00	0.00	0.02
Dabbling	15.14	3.66	0.06	3.73	2.09	4.45	2.16
Diving	0.01	0.00	0.05	0.00	0.00	0.01	0.00
Domestic	0.01	0.01	0.01	0.00	0.00	0.00	0.00
DHP3	0.00	0.00	0.00	0.00	0.00	0.00	0.00
Pha	0.00	0.00	0.00	0.00	0.00	0.00	0.00
Gull	0.00	0.00	0.01	0.00	0.00	0.00	0.00
DHP4	0.30	0.00	0.01	0.00	2.08	0.00	0.00
DHP5	0.02	0.00	0.01	0.26	0.00	0.01	0.00
Other Water Birds	0.00	0.00	0.03	0.00	0.00	0.00	0.00
Other Waterfowl	0.14	0.00	0.02	0.00	0.00	0.00	0.00
DHP6	0.01	0.00	0.03	0.00	0.01	0.00	0.00
Shorebird	0.09	0.01	0.16	0.21	0.06	0.09	0.01
DHP7	0.01	0.08	0.02	0.00	0.00	0.00	0.00

**PB1**	DHP1	DHP2	DHP3	DHP4	DHP5	DHP6	DHP7
DHP1	0.00	3.32	0.16	0.09	3.20	0.18	1.57
DHP2	0.06	0.00	0.01	0.00	0.04	0.00	0.01
Dabbling	16.31	0.55	0.06	3.10	0.57	4.68	0.95
Diving	0.01	0.01	0.04	0.00	0.01	0.00	0.00
Domestic	0.01	0.01	0.01	0.00	0.00	0.00	0.00
DHP3	0.01	0.00	0.00	0.00	0.00	0.00	0.00
Pha	0.00	0.00	0.00	0.00	0.00	0.00	0.00
Gull	0.00	0.00	0.00	0.01	0.00	0.00	0.00
DHP4	0.00	0.01	0.01	0.00	1.77	0.00	0.00
DHP5	0.00	0.06	0.01	0.81	0.00	0.00	0.00
Other Water Birds	0.00	0.00	0.01	0.00	0.00	0.00	0.00
Other Waterfowl	0.02	0.01	0.03	0.02	0.02	0.00	0.00
DHP6	0.01	0.03	0.03	0.00	0.00	0.00	0.00
Shorebird	0.02	0.00	0.02	0.31	0.43	0.01	0.00
DHP7	0.06	0.01	0.02	0.00	0.00	0.01	0.00

**PA EA**	DHP1	DHP2	DHP3	DHP4	DHP5	DHP6	DHP7
DHP1	0.00	4.74	0.07	0.69	4.26	0.00	1.76
DHP2	0.03	0.00	0.01	0.00	0.00	0.00	0.02
Dabbling	15.64	0.41	0.18	2.76	0.10	7.68	0.69
Diving	0.09	0.01	0.07	0.00	0.01	0.02	0.14
Domestic	0.02	0.00	0.02	0.01	0.00	0.00	0.00
DHP3	0.00	0.00	0.00	0.00	0.00	0.00	0.00
Pha	0.00	0.00	0.01	0.00	0.00	0.00	0.00
Gull	0.02	0.00	0.01	0.00	0.01	0.00	0.00
DHP4	0.03	0.00	0.04	0.00	0.19	0.00	0.01
DHP5	0.01	0.00	0.00	2.50	0.00	0.00	0.03
Other Water Birds	0.02	0.00	0.02	0.01	0.00	0.00	0.01
Other Waterfowl	0.10	0.14	0.08	0.00	0.01	0.00	0.01
DHP6	0.01	0.01	0.10	0.00	0.01	0.00	0.02
Shorebird	0.03	0.01	0.05	0.06	0.84	0.01	0.01
DHP7	0.00	0.00	0.02	0.00	0.01	0.00	0.00

**PA NA**	DHP1	DHP2	DHP4	DHP5	DHP7
DHP1	0.00	0.05	0.03	0.42	0.45
DHP2	0.00	0.00	0.00	0.07	0.01
Dabbling	15.85	0.31	2.57	2.95	0.08
Diving	0.06	0.03	0.00	0.01	0.03
Domestic	0.01	0.49	0.00	0.01	0.02
Pha	0.01	0.02	0.00	0.00	0.00
Gull	0.02	0.01	0.00	0.01	0.03
DHP4	0.01	0.01	0.00	0.01	0.02
DHP5	0.01	1.44	0.00	0.00	0.01
Other Water Birds	0.01	0.02	0.00	0.02	0.03
Other Waterfowl	0.17	0.04	0.00	0.02	0.04
Shorebird	0.97	0.16	0.01	0.63	0.60
DHP7	0.03	0.00	0.00	0.00	0.00

**NS A**	DHP1	DHP2	DHP3	DHP4	DHP5	DHP6	DHP7
DHP1	0.00	2.67	0.08	0.08	2.04	0.04	0.93
DHP2	0.34	0.00	0.01	0.04	0.02	0.00	0.01
Dabbling	16.84	1.05	0.11	3.33	2.38	3.43	1.48
Diving	0.00	0.01	0.05	0.00	0.00	0.00	0.03
Domestic	0.06	0.04	0.12	0.00	0.08	0.11	0.06
DHP3	0.00	0.00	0.00	0.00	0.00	0.00	0.00
Pha	0.00	0.00	0.01	0.00	0.00	0.01	0.00
Gull	0.01	0.00	0.01	0.02	0.02	0.00	0.00
DHP4	0.01	0.50	0.05	0.00	1.82	0.00	0.04
DHP5	0.03	0.34	0.02	1.58	0.00	0.00	0.14
Other Water Birds	0.00	0.00	0.02	0.00	0.01	0.00	0.00
Other Waterfowl	0.03	0.01	0.06	0.01	0.01	0.03	0.04
DHP6	0.00	0.00	0.05	0.00	0.00	0.00	0.02
Shorebird	1.97	0.02	0.09	1.06	1.21	0.02	0.03
DHP7	0.01	0.01	0.02	0.01	0.03	0.01	0.00

**NS B**	DHP1	DHP2	DHP4	DHP5	DHP6	DHP7
DHP1	0.00	4.64	0.06	1.07	0.97	0.04
DHP2	0.33	0.00	0.00	0.01	0.08	0.00
Dabbling	12.66	0.88	2.79	2.77	6.39	0.78
Diving	0.01	0.01	0.06	0.03	0.00	0.07
Domestic	0.00	0.00	0.01	0.00	0.00	0.01
Pha	0.00	0.00	0.01	0.00	0.00	0.00
Gull	0.01	0.00	0.01	0.01	0.01	0.01
DHP4	0.00	0.01	0.00	0.01	0.00	0.60
DHP5	0.12	0.01	0.02	0.00	0.04	0.02
Other Water Birds	0.00	0.00	0.00	0.00	0.00	0.00
Other Waterfowl	0.02	0.05	0.05	0.02	0.01	0.06
DHP6	0.01	0.01	0.06	0.03	0.00	0.05
Shorebird	0.12	0.02	0.03	0.02	0.02	0.04
DHP7	0.00	0.01	0.83	0.00	0.01	0.00

**NP**	DHP1	DHP2	DHP3	DHP4	DHP5	DHP6	DHP7
DHP1	0.00	0.87	0.07	0.30	2.91	0.02	0.81
DHP2	0.54	0.00	0.02	0.00	0.04	0.00	0.00
Dabbling	17.26	2.63	0.06	3.34	0.63	5.17	1.50
Diving	0.02	0.01	0.10	0.00	0.01	0.00	0.00
Domestic	0.00	0.01	0.02	0.00	0.00	0.00	0.00
DHP3	0.00	0.00	0.00	0.00	0.00	0.00	0.00
Pha	0.00	0.00	0.01	0.00	0.00	0.00	0.00
Gull	0.00	0.00	0.00	0.01	0.00	0.00	0.00
DHP4	0.42	0.00	0.02	0.00	1.79	0.00	0.00
DHP5	0.03	0.24	0.01	0.36	0.00	0.00	0.00
Other Water Birds	0.00	0.00	0.01	0.00	0.00	0.00	0.00
Other Waterfowl	0.01	0.04	0.05	0.03	0.05	0.00	0.00
DHP6	0.01	0.00	0.03	0.00	0.00	0.00	0.07
Shorebird	0.37	0.01	0.04	0.43	0.17	0.00	0.01
DHP7	0.01	0.00	0.03	0.00	0.00	0.02	0.00

**MP**	DHP1	DHP2	DHP3	DHP4	DHP5	DHP6	DHP7
DHP1	0.00	1.90	0.30	0.42	1.54	0.70	1.77
DHP2	0.71	0.00	0.01	0.00	0.02	0.03	0.00
Dabbling	17.66	2.20	0.02	2.69	1.89	4.52	0.21
Diving	0.01	0.00	0.01	0.00	0.00	0.00	0.01
Domestic	0.14	0.01	0.01	0.01	0.01	0.00	0.00
DHP3	0.00	0.00	0.00	0.00	0.00	0.00	0.00
Gal	0.01	0.00	0.00	0.00	0.00	0.00	0.00
Gull	0.00	0.00	0.00	0.02	0.01	0.00	0.00
DHP4	0.08	0.00	0.01	0.00	0.87	0.00	0.00
DHP5	0.02	0.02	0.01	0.73	0.00	0.00	0.00
Other Water Birds	0.02	0.00	0.00	0.00	0.00	0.00	0.00
Other Waterfowl	0.02	0.00	0.01	0.00	0.00	0.00	0.00
DHP6	0.02	0.16	0.01	0.00	0.00	0.00	0.01
Shorebird	0.02	0.00	0.01	0.99	1.44	0.00	0.11
DHP7	0.00	0.00	0.02	0.00	0.00	0.00	0.00

Supplementary Table 5. Percentage of Markov jump counts for the Taxonomy trait

**PB2**	DHP1	DHP2	DHP3	DHP4	DHP5	DHP6	DHP7
Anatidae	21.05	5.41	0.16	5.08	3.20	6.28	3.03
DHP2	0.28	0.00	0.04	0.00	0.01	0.01	0.03
DHP1	0.00	0.64	0.06	0.06	1.21	0.96	0.03
DHP3	0.00	0.00	0.00	0.00	0.00	0.00	0.00
Phasianidae	0.00	0.00	0.01	0.00	0.00	0.00	0.00
DHP4	0.40	0.00	0.02	0.00	2.77	0.00	0.00
DHP5	0.02	0.00	0.03	0.40	0.00	0.01	0.00
Neoaves	0.11	0.01	0.21	0.29	0.10	0.17	0.01
DHP6	0.02	0.00	0.06	0.00	0.00	0.00	0.00
DHP7	0.01	0.12	0.03	0.00	0.00	0.00	0.00

**PB1**	DHP1	DHP2	DHP3	DHP4	DHP5	DHP6	DHP7
Anatidae	21.05	3.39	0.26	4.87	2.57	7.07	2.50
DHP2	0.47	0.00	0.02	0.00	0.17	0.00	0.08
DHP1	0.00	2.01	0.09	0.01	3.00	0.08	0.96
DHP3	0.00	0.00	0.00	0.00	0.00	0.00	0.00
Phasianidae	0.00	0.00	0.03	0.00	0.00	0.00	0.00
DHP4	0.00	0.00	0.03	0.00	2.89	0.00	0.00
DHP5	0.02	0.17	0.03	0.58	0.00	0.00	0.00
Neoaves	0.02	0.01	0.08	0.57	0.27	0.02	0.00
DHP6	0.01	0.01	0.08	0.00	0.00	0.00	0.00
DHP7	0.32	0.02	0.03	0.00	0.02	0.00	0.00

**PA EA**	DHP1	DHP2	DHP3	DHP4	DHP5	DHP6	DHP7
Anatidae	23.07	1.82	0.45	4.75	0.33	11.46	2.04
DHP2	0.27	0.00	0.03	0.00	0.01	0.00	0.04
DHP1	0.00	6.01	0.12	0.36	6.11	0.01	1.80
DHP3	0.00	0.00	0.00	0.00	0.00	0.00	0.00
Phasianidae	0.00	0.00	0.02	0.01	0.00	0.00	0.00
DHP4	0.04	0.00	0.06	0.00	0.17	0.00	0.02
DHP5	0.01	0.00	0.01	3.74	0.00	0.00	0.09
Neoaves	0.04	0.01	0.09	0.06	1.36	0.03	0.03
DHP6	0.01	0.01	0.17	0.01	0.02	0.00	0.03
DHP7	0.00	0.00	0.05	0.00	0.02	0.00	0.00

**PA NA**	DHP1	DHP2	DHP4	DHP5	DHP7
Anatidae	23.00	1.73	3.76	4.45	0.20
DHP2	0.01	0.00	0.00	0.25	0.01
DHP1	0.00	0.06	0.04	0.44	0.63
Phasianidae	0.02	0.01	0.00	0.01	0.01
DHP4	0.01	0.02	0.00	0.02	0.02
DHP5	0.02	1.76	0.01	0.00	0.03
Neoaves	1.46	0.17	0.01	0.74	0.99
DHP7	0.05	0.00	0.00	0.01	0.00

**NS A**	DHP1	DHP2	DHP3	DHP4	DHP5	DHP6	DHP7
Anatidae	21.15	4.23	0.26	4.64	4.29	4.77	3.19
DHP2	1.05	0.00	0.04	0.08	0.04	0.00	0.00
DHP1	0.00	0.54	0.12	0.01	1.42	0.02	0.37
DHP3	0.00	0.00	0.00	0.00	0.00	0.00	0.00
Phasianidae	0.00	0.02	0.15	0.00	0.17	0.00	0.01
DHP4	0.01	0.52	0.05	0.00	2.39	0.01	0.02
DHP5	0.02	0.33	0.04	1.58	0.00	0.00	0.09
Neoaves	1.64	0.01	0.10	1.39	1.38	0.01	0.01
DHP6	0.01	0.01	0.09	0.00	0.00	0.00	0.01
DHP7	0.02	0.02	0.07	0.01	0.04	0.00	0.00

**NS B**	DHP1	DHP2	DHP4	DHP5	DHP6	DHP7
Anatidae	18.87	3.41	4.75	5.05	10.27	1.58
DHP2	1.37	0.00	0.01	0.02	0.08	0.02
DHP1	0.00	5.58	0.06	1.25	1.73	0.07
Phasianidae	0.00	0.00	0.01	0.00	0.00	0.01
DHP4	0.00	0.01	0.00	0.01	0.01	0.71
DHP5	0.22	0.02	0.03	0.00	0.04	0.04
Neoaves	0.24	0.03	0.06	0.04	0.02	0.07
DHP6	0.02	0.02	0.04	0.04	0.00	0.07
DHP7	0.00	0.00	1.41	0.01	0.01	0.00

**NP**	DHP1	DHP2	DHP3	DHP4	DHP5	DHP6	DHP7
Anatidae	22.88	4.67	0.21	4.78	2.38	7.49	3.22
DHP2	1.01	0.00	0.04	0.00	0.08	0.00	0.00
DHP1	0.00	0.39	0.11	0.29	2.95	0.01	0.26
DHP3	0.00	0.00	0.00	0.00	0.00	0.00	0.00
Phasianidae	0.00	0.00	0.02	0.00	0.00	0.00	0.00
DHP4	0.57	0.00	0.03	0.00	2.28	0.00	0.00
DHP5	0.04	0.33	0.02	0.67	0.00	0.01	0.00
Neoaves	0.67	0.01	0.08	0.64	0.27	0.00	0.02
DHP6	0.00	0.00	0.07	0.00	0.01	0.00	0.04
DHP7	0.02	0.00	0.07	0.00	0.00	0.03	0.00

**MP**	DHP1	DHP2	DHP3	DHP4	DHP5	DHP6	DHP7
Anatidae	25.70	5.66	0.06	4.34	4.72	7.74	1.02
DHP2	1.49	0.00	0.01	0.00	0.04	0.03	0.00
DHP1	0.00	0.13	0.51	0.32	0.64	0.09	1.92
DHP3	0.00	0.00	0.00	0.00	0.00	0.00	0.00
Phasianidae	0.00	0.00	0.00	0.00	0.00	0.00	0.00
DHP4	0.11	0.00	0.01	0.00	1.17	0.01	0.01
DHP5	0.04	0.04	0.01	0.96	0.00	0.01	0.01
Neoaves	0.02	0.00	0.01	1.54	2.01	0.00	0.22
DHP6	0.02	0.24	0.01	0.00	0.00	0.00	0.01
DHP7	0.01	0.00	0.02	0.00	0.01	0.00	0.00

Supplementary Table 6. Percentage of Markov jump counts for the trait regarding the number of introductions per year

**PB2**	2003	2004	2005	2006	2007	2008	2009	2010	2011	2012	2013
2003	0.00	0.00	0.00	0.00	0.00	0.00	0.00	0.00	0.00	0.00	0.00
2004	0.00	0.00	0.00	0.00	0.00	0.00	0.00	0.00	0.00	0.00	0.00
2005	0.01	0.02	0.00	0.04	0.00	0.00	0.00	0.00	0.00	0.00	0.00
2006	0.00	0.00	0.02	0.00	1.29	0.00	0.00	0.00	0.01	0.00	0.00
2007	0.00	0.00	0.00	0.01	0.00	0.00	0.33	0.00	0.00	0.00	0.00
2008	0.00	0.00	0.00	0.00	0.00	0.00	0.18	0.00	0.00	0.00	0.00
2009	0.00	0.00	0.00	0.00	0.02	0.14	0.00	0.27	0.00	0.00	0.01
2010	0.01	0.00	0.00	0.00	0.00	0.00	0.05	0.00	1.15	0.16	0.00
2011	0.00	0.00	0.00	0.00	0.00	0.00	0.00	0.02	0.00	0.15	0.00
2012	0.00	0.00	0.00	0.00	0.00	0.00	0.00	0.05	0.08	0.00	0.00
2013	0.00	0.00	0.00	0.00	0.00	0.00	0.00	0.00	0.00	0.00	0.00
USA	4.69	2.52	15.99	15.28	8.64	6.27	12.21	7.30	10.30	6.10	5.10

**PB1**	2003	2004	2005	2006	2007	2008	2009	2010	2011	2012	2013
2003	0.00	0.00	0.01	0.11	0.00	0.00	0.00	0.00	0.00	0.00	0.00
2004	0.00	0.00	0.00	0.00	0.00	0.00	0.00	0.00	0.00	0.00	0.00
2005	0.01	0.01	0.00	0.02	0.41	0.00	0.00	0.00	0.00	0.01	0.00
2006	0.01	0.05	0.02	0.00	1.11	0.00	0.00	0.00	0.00	0.01	0.01
2007	0.00	0.00	0.14	0.01	0.00	0.00	0.00	0.00	0.00	0.00	0.00
2008	0.00	0.00	0.00	0.00	0.00	0.00	1.42	0.00	0.00	0.00	0.00
2009	0.01	0.00	0.00	0.00	0.01	0.01	0.00	0.00	0.00	0.01	0.01
2010	0.00	0.00	0.00	0.00	0.00	0.00	0.00	0.00	1.74	0.00	0.00
2011	0.00	0.00	0.00	0.00	0.00	0.00	0.00	0.08	0.00	0.01	0.00
2012	0.00	0.00	0.00	0.00	0.00	0.00	0.00	0.00	0.00	0.00	0.00
2013	0.01	0.00	0.00	0.00	0.00	0.00	0.00	0.00	0.00	0.00	0.00
USA	4.06	2.63	16.07	15.53	6.44	6.76	13.46	8.11	8.09	5.39	5.39

**PA EA**	2003	2004	2005	2006	2007	2008	2009	2010	2011	2012	2013
2003	0.00	0.01	1.34	0.01	0.00	0.00	0.00	0.00	0.00	0.01	0.00
2004	0.01	0.00	0.07	0.03	0.00	0.00	0.01	0.00	0.00	0.02	0.00
2005	1.48	0.15	0.00	2.40	0.00	0.00	0.00	0.00	0.00	0.02	0.01
2006	0.05	0.20	2.44	0.00	0.00	0.01	0.01	0.01	0.00	0.04	0.01
2007	0.02	0.01	0.01	0.00	0.00	0.00	0.39	0.01	0.00	0.01	0.00
2008	0.03	0.01	0.00	0.00	0.00	0.00	0.00	0.00	0.00	0.01	0.01
2009	0.05	0.01	0.03	0.01	0.10	0.00	0.00	0.78	0.00	0.04	0.01
2010	0.02	0.02	0.01	0.01	0.00	0.00	0.01	0.00	2.94	0.03	0.01
2011	0.04	0.02	0.02	0.00	0.00	0.00	0.00	0.06	0.00	0.04	0.01
2012	0.01	0.01	0.01	0.00	0.00	0.00	0.00	0.00	0.00	0.00	0.00
2013	0.03	0.00	0.01	0.00	0.00	0.00	0.00	0.00	0.00	0.01	0.00
USA	4.08	3.48	8.79	10.90	5.10	5.21	11.88	9.67	9.14	3.25	5.14

**PA NA**	2005	2006	2007	2008	2009	2011	2012	2013
2005	0.00	0.01	0.01	0.02	0.00	0.07	0.00	0.02
2006	0.00	0.00	0.03	0.75	0.01	0.11	0.00	0.03
2007	0.01	0.05	0.00	0.02	0.00	0.11	0.01	0.04
2008	0.00	0.09	0.00	0.00	0.00	0.07	0.01	0.02
2009	0.00	0.01	0.01	0.02	0.00	0.08	0.00	0.02
2011	0.00	0.01	0.00	0.01	0.00	0.00	0.00	0.01
2012	0.00	0.01	0.01	0.02	0.01	0.12	0.00	1.14
2013	0.00	0.01	0.00	0.02	0.00	0.07	0.16	0.00
USA	9.21	20.57	17.45	8.38	9.22	5.04	15.57	7.93

**NS A**	2003	2004	2005	2006	2007	2008	2009	2010	2011	2012	2013
2003	0.00	0.03	0.40	0.02	0.01	0.00	0.01	0.00	0.01	0.01	0.01
2004	0.00	0.00	0.01	0.01	0.00	0.00	0.00	0.00	0.00	0.00	0.00
2005	0.08	0.08	0.00	0.14	1.59	0.00	0.02	0.00	0.01	0.02	0.01
2006	0.02	0.13	0.11	0.00	0.28	0.01	0.50	0.01	0.01	0.02	0.01
2007	0.01	0.03	0.01	0.04	0.00	0.00	0.02	0.00	0.01	0.01	0.01
2008	0.00	0.03	0.00	0.01	0.00	0.00	0.10	0.00	0.01	0.01	0.00
2009	0.01	0.06	0.01	0.02	0.01	0.03	0.00	0.00	0.02	0.03	0.01
2010	0.00	0.02	0.00	0.00	0.01	0.00	0.01	0.00	1.85	0.01	0.01
2011	0.00	0.02	0.00	0.00	0.01	0.00	0.00	0.08	0.00	0.01	0.00
2012	0.00	0.02	0.01	0.00	0.00	0.00	0.00	0.00	0.01	0.00	0.07
2013	0.00	0.03	0.01	0.00	0.01	0.00	0.01	0.00	0.01	0.10	0.00
USA	6.68	1.33	16.06	16.77	8.58	5.61	16.48	6.05	3.50	3.17	4.99

**NS B**	2005	2006	2007	2008	2009	2010	2011	2012	2013
2005	0.00	0.02	0.01	0.00	0.01	0.00	0.01	0.01	0.08
2006	0.08	0.00	0.01	0.00	0.02	0.01	0.01	0.01	0.14
2007	0.09	0.04	0.00	0.00	0.02	0.00	0.01	0.01	0.17
2008	0.08	0.03	0.01	0.00	2.98	0.04	0.02	0.01	0.11
2009	0.09	0.03	0.01	0.10	0.00	0.49	0.74	0.01	0.16
2010	0.09	0.03	0.01	0.01	0.95	0.00	0.58	0.01	0.15
2011	0.14	0.05	0.02	0.01	0.16	0.11	0.00	0.14	0.25
2012	0.12	0.03	0.01	0.00	0.01	0.01	0.03	0.00	0.20
2013	0.04	0.01	0.01	0.00	0.02	0.00	0.01	0.00	0.00
USA	3.59	8.35	12.76	9.02	11.77	9.20	19.94	12.70	3.01

**NP**	2003	2004	2005	2006	2007	2008	2009	2010	2011	2012	2013
2003	0.00	0.00	0.00	0.01	0.00	0.00	0.00	0.00	0.00	0.00	0.00
2004	0.00	0.00	0.04	0.00	0.00	0.00	0.00	0.00	0.00	0.00	0.00
2005	0.00	0.11	0.00	0.03	0.00	0.00	0.00	0.00	0.01	0.01	0.00
2006	0.02	0.01	0.03	0.00	0.02	0.01	0.02	0.00	0.01	0.01	0.01
2007	0.01	0.00	0.00	0.04	0.00	0.01	0.00	0.00	0.00	0.01	0.00
2008	0.00	0.00	0.01	0.01	0.00	0.00	0.00	0.00	0.00	0.00	0.00
2009	0.00	0.00	0.00	0.01	0.00	0.00	0.00	0.00	0.00	0.01	0.00
2010	0.00	0.00	0.00	0.00	0.00	0.00	0.00	0.00	1.87	0.01	0.00
2011	0.01	0.00	0.00	0.00	0.00	0.00	0.00	0.36	0.00	0.00	0.02
2012	0.00	0.00	0.00	0.00	0.00	0.00	0.00	0.00	0.00	0.00	0.00
2013	0.00	0.00	0.00	0.01	0.00	0.00	0.00	0.00	0.01	0.00	0.00
USA	5.29	2.51	14.70	15.17	9.13	6.27	11.82	7.64	9.76	3.90	6.54

**MP**	2003	2004	2005	2006	2007	2008	2009	2010	2011	2012	2013
2003	0.00	0.00	0.00	0.00	0.00	0.00	0.00	0.00	0.00	0.00	0.00
2004	0.00	0.00	0.00	0.01	0.05	0.00	0.00	0.00	0.00	0.00	0.00
2005	0.01	0.00	0.00	0.02	0.00	0.00	0.00	0.00	0.00	0.00	0.00
2006	0.01	0.02	0.01	0.00	0.48	0.00	0.01	0.00	0.01	0.00	0.00
2007	0.00	0.05	0.00	0.42	0.00	0.01	0.03	0.02	0.02	0.00	0.01
2008	0.00	0.00	0.00	0.00	0.00	0.00	0.00	0.00	0.00	0.00	0.00
2009	0.01	0.00	0.00	0.00	0.01	0.00	0.00	0.17	0.01	0.00	0.01
2010	0.00	0.00	0.00	0.00	0.00	0.00	0.02	0.00	0.63	0.00	0.01
2011	0.00	0.00	0.00	0.00	0.00	0.00	0.00	0.02	0.00	0.00	0.05
2012	0.00	0.00	0.00	0.00	0.00	0.00	0.00	0.00	0.00	0.00	0.02
2013	0.00	0.00	0.00	0.00	0.00	0.00	0.00	0.00	0.03	0.01	0.00
USA	2.33	2.23	12.54	15.27	10.20	5.91	16.39	8.05	10.56	5.71	5.64
